# Photo-Catalyzed Synthesis of Indanones from Aromatic Aldehydes and Terminal Alkynes

**DOI:** 10.1021/acs.joc.5c01749

**Published:** 2025-09-22

**Authors:** Florence Babawale, Indrajit Ghosh, Burkhard König

**Affiliations:** Fakultät für Chemie und Pharmazie, 48278 Universität Regensburg, Regensburg 93040, Germany

## Abstract

Indanones are key structural motifs in pharmaceuticals and bioactive natural products, making their efficient synthesis a subject of continued interest. Conventional methods typically rely on transition-metal catalysis, prefunctionalized substrates, and multiple redox steps. Herein, we report a photochemical C–H annulation strategy for the direct synthesis of indanones from simple, unmodified aromatic aldehydes and terminal alkynes. The reaction proceeds under 365 nm light using tetrabutylphosphonium decatungstate ([Bu_4_P]_4_W_10_O_32_, TBPDT) as a hydrogen atom transfer (HAT) photocatalyst. This prefunctionalization-free protocol tolerates a broad range of substrates, eliminates the need for additional redox steps, and expands the toolbox for sustainable indanone synthesis.

## Graphical abstract

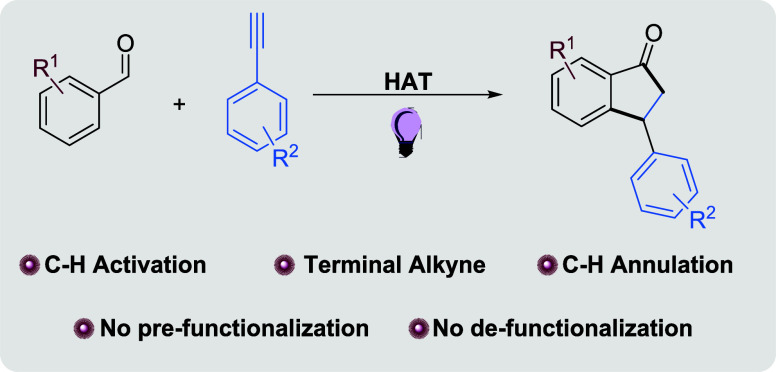

## Introduction

Indanones are an important class of organic compounds commonly found in pharmaceuticals, agrochemicals, and bioactive natural products. Their structural motifs appear in several biologically active molecules, such as fredericamycin A (an antitumor antibiotic),^1^ exiguaquinol (an inhibitor of the *Helicobacter pylori* *MurI* enzyme),^2^ pauciflorol F (a polyphenolic compound with diverse biological activities),^3^ and others such as meroindenon and coneuplectin.^4,5^ Given their broad utility and pharmacological significance, the development of efficient, selective, and sustainable synthetic routes to indanones remains a central goal in organic synthesis.

Traditionally, indanones are synthesized via transition-metal-catalyzed annulation of carbonyl compounds with alkynes, often requiring prefunctionalized substrates such as *ortho*-halogenated carbonyl derivatives (Scheme 1B). These strategies typically involve additional oxidation or reduction steps to convert intermediates such as indenols or indenones into the desired indanone scaffold, increasing synthetic complexity and limiting scalability.^6–19^ Although rhodium-catalyzed methods can bypass the need for prefunctionalization, they still necessitate subsequent reduction steps to obtain the final indanone products (Scheme 1B).^20–25^ Thus, there is a continuing demand for more direct and step-economical approaches that enable C–H annulation of simple carbonyl compounds with alkynes.

**Scheme 1:**
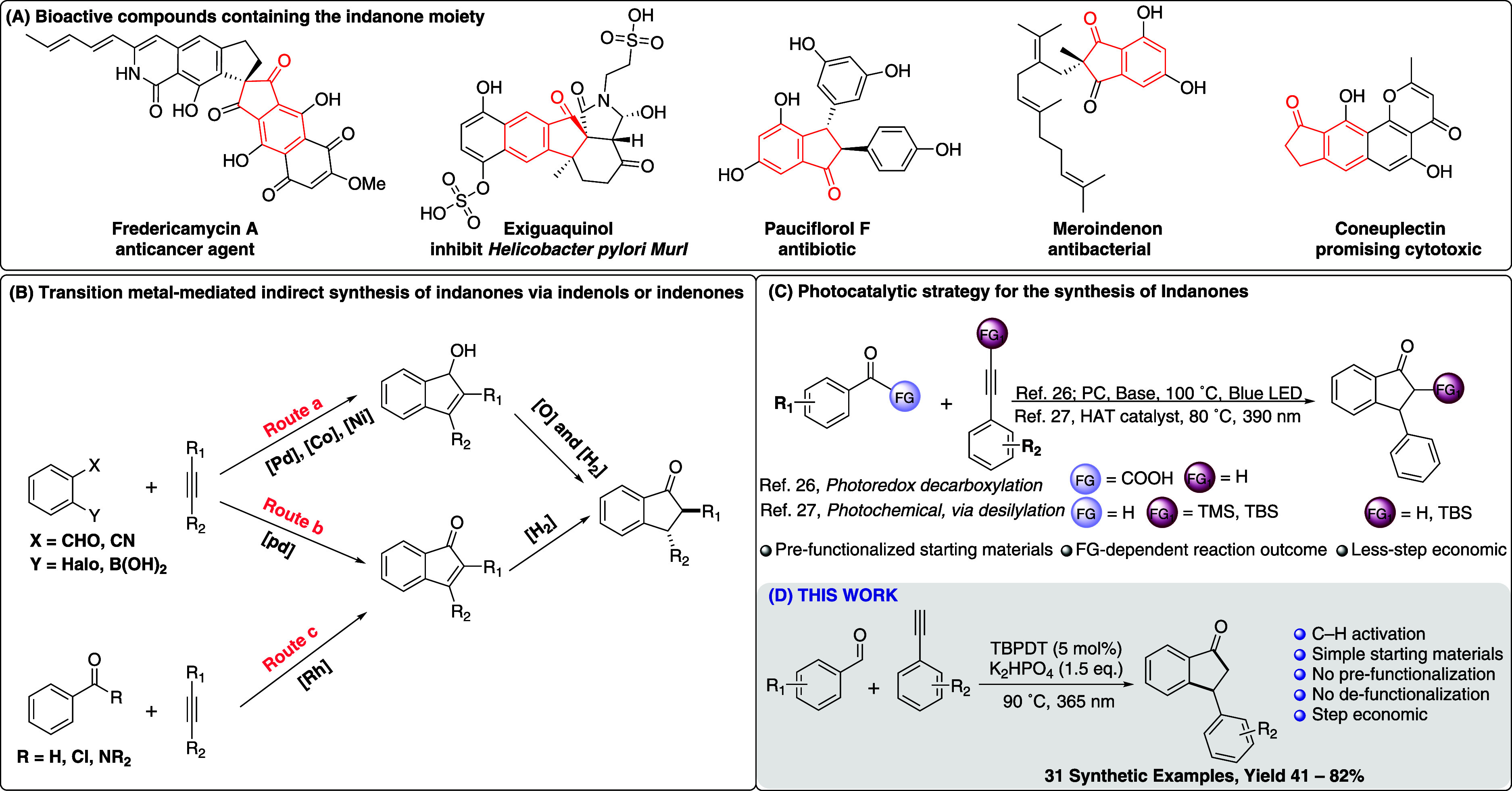
Overview of Strategies for the Synthesis of Indanone Motifs and This Work

In recent years, photochemical methods have gained prominence as powerful tools in organic synthesis, offering mild reaction conditions and the ability to generate reactive radical intermediates under environmentally benign conditions. Several photochemical approaches to indanone synthesis have been developed. For example, Lan, Zhu, and coworkers reported a decarboxylative annulation of carbonyl compounds with alkynes using an iridium-based photoredox catalyst, achieving efficient and selective cyclization (Scheme 1C).^26^ Similarly, Wang and coworkers demonstrated a radical-mediated photochemical cyclization using silyl-protected alkynes.^27^

While these methods represent significant progress, they still rely on prefunctionalized substrates, and the reaction outcomes are often highly sensitive to the nature of the functional group. For instance, Wang and coworkers reported that when trimethyl­(phenylethynyl)­silane (TMS) was used, *in situ* desilylation led to the formation of functionalized indanones. In contrast, the use of *tert*-butyldimethyl­(phenylethynyl)­silane (TBS) retained the silyl group, highlighting the challenges in selecting appropriate functional groups and the need for additional functionalization or defunctionalization steps to access the desired indanone products (Scheme 1C).^27^

Herein, we report a direct C–H activation, radical-mediated annulation for the synthesis of functionalized indanones from simple, unmodified aromatic aldehydes and terminal alkynes using tetrabutylphosphonium decatungstate ([Bu_4_P]_4_W_10_O_32_, TBPDT) as a hydrogen atom transfer (HAT) photocatalyst. Photoinduced HAT catalysis has recently emerged as an effective strategy for generating acyl radicals directly via C–H abstraction from aldehydes.^28–31^ Upon photoexcitation, HAT catalysts such as diaryl ketones,^28^ Eosin Y,^29^ and decatungstate salts^30^ can abstract hydrogen atoms from aldehydes to form nucleophilic acyl radicals. We hypothesized that these radicals would undergo addition to electron-deficient alkynes, generating vinyl radical intermediates that subsequently engage in intramolecular [3 + 2] cyclization to furnish the indanone core.

This direct, radical-mediated annulation strategy provides a general, practical, and sustainable alternative to conventional methods. It proceeds without the need for prefunctionalized substrates, external oxidants or reductants, or protecting group manipulations, allowing efficient access to a diverse array of indanone derivatives.

## Results and Discussion

We began our synthetic investigation using benzaldehyde as the precursor of the acyl radical, generated via 365 nm light excitation of TBPDT, and phenylacetylene as the radical acceptor. Initial reactions performed at 60 °C resulted in modest yields of the desired indanone product and significant formation of side products, particularly 3-phenyl-2,3-dihydro-1H-inden-1-ol (see Table S1). Increasing the temperature improved the product yield while minimizing the formation of byproducts. Notably, although water was not essential for obtaining the desired product, the addition of 100 μL improved the solubility of both TBPDT and K_2_HPO_4_, thereby enhancing the product yield. The beneficial effect of trace amounts of water on photochemical reactions involving decatungstate catalysts has been previously reported.^32^ The reactions did not proceed in the absence of light, confirming the essential role of light in forming the desired product. Similarly, the photochemical reaction was less effective without both TBPDT and K_2_HPO_4_. While the absence of either TBPDT or K_2_HPO_4_ still resulted in product formation, the presence of both consistently led to higher yields of the desired product.

With the optimized reaction conditions in hand (Table 1, entry 1), we next evaluated the generality of the protocol using a range of substituted arylacetylenes, including both electron-rich and electron-deficient variants, as well as positional isomers (Figure 1). To our delight, the method exhibited broad substrate scope, and a variety of functional groups were well tolerated, including methoxy (**3b**–**3d**), chloro (**3f**, **3g**), fluoro (**3h**, **3i**), dihalides (**3k**), trifluoromethyl and bis-trifluoromethyl (**3j**, **3l**), and ester (**3m**), affording the corresponding indanones in isolated yields ranging from 41% to 73%. Notably, alkynes bearing a boronic ester moiety (**3n**) also gave the desired product in 69% isolated yield. Furthermore, heteroaryl alkynes, such as 3-ethynylpyridine, proved to be suitable reaction partners, furnishing the desired product **3o** in 77% isolated yield.

**Table 1:**
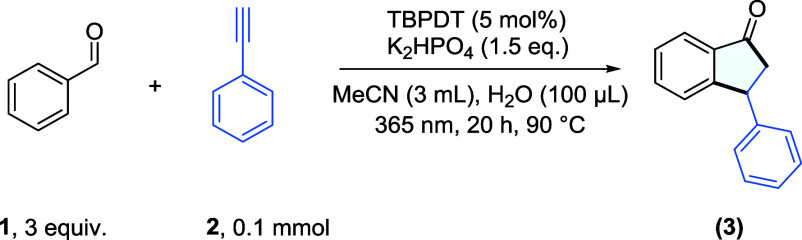
Optimization of Reaction Conditions and Results of the Control Experiments

Entry	**Deviation from the standard reaction condition**	**3 (% yield)** ^a^
1	None	72
Control experiments		
2	No light	0
3	No TBPDT, No K_2_HPO_4_	17
4	No K_2_HPO_4_	44
5	No TBPDT	51
6	No water	54
Effect of other catalysts		
7	TBADT	65
8	10 mol % Benzophenone	56
Effect of other bases		
9	2,6 Lutidine	60
10	Na_2_CO_3_	59
Effect of temperature		
11	25 °C	30
12	60 °C	48
Others		
13	2 mL Solvent [0.05 M]	61
14	4 mL Solvent [0.025 M]	58
15^b^	No degassing	37

a Yields were determined (within analytical errors, ±5%) by GC-FID using 1,3,5-trimethoxybenzene as an internal standard. The reactions were run under nitrogen atmosphere unless noted otherwise. An oil bath was used as the heat source and to maintain the reaction temperature.

b The photochemical reaction was performed just by closing the reaction vial without degassing.

**Figure 1:**
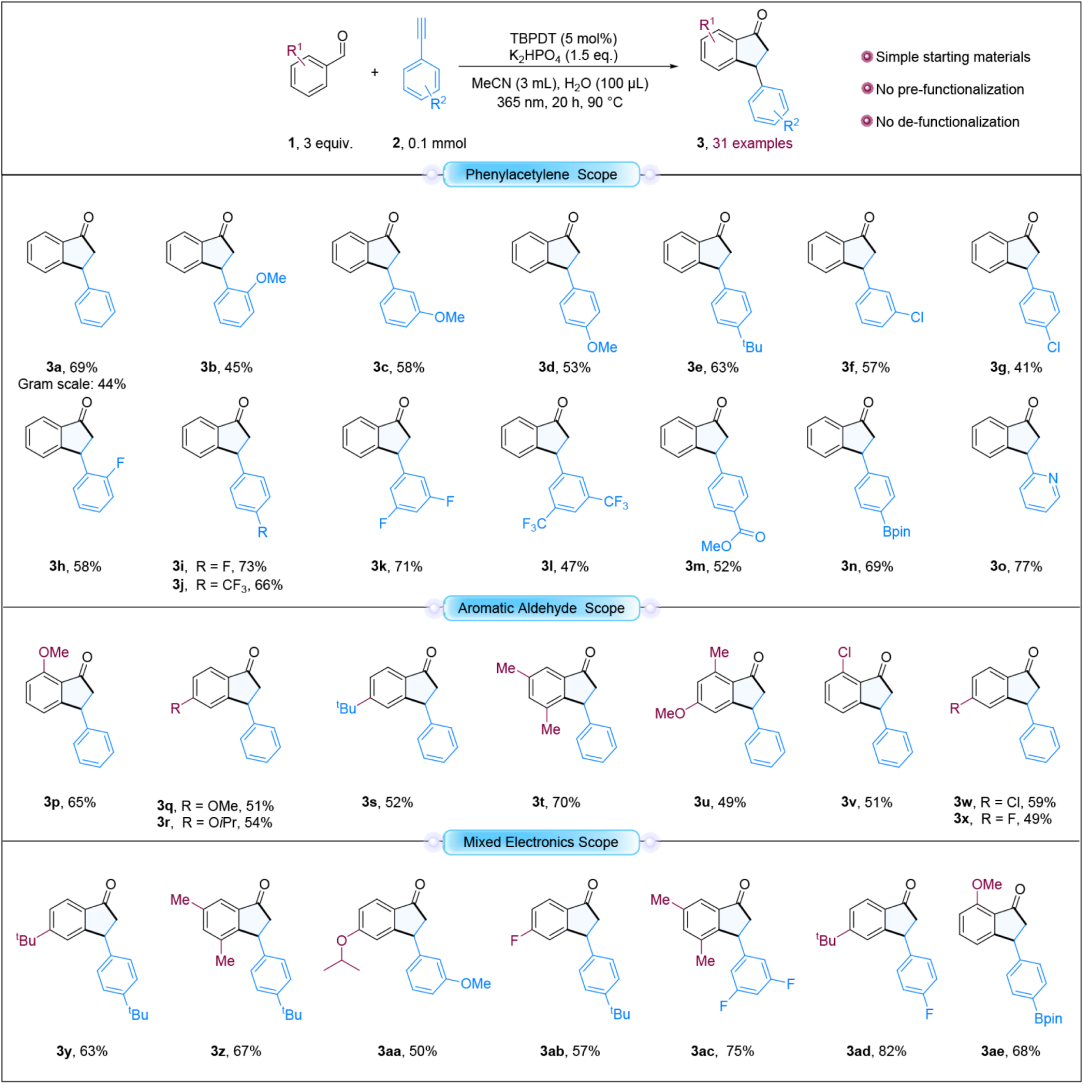
Synthetic examples of C–H annulation of simple carbonyl compounds with alkynes exploring both the alkyne and carbonyl scope. An oil bath was used as the heat source and to maintain the reaction temperature. Isolated yields are reported unless noted otherwise.

We next examined the effect of substituent position on reactivity. Substrates bearing ortho, meta, and para substituents were all competent, with meta-substituted arylacetylenes consistently affording higher yields than their ortho- and para-substituted analogues (cf. **3b**, **3c**, **3d** for the −OMe substituent). This trend was particularly evident in the case of chloro-substituted alkynes (cf. **3f**, **3g**).

The scope of aromatic aldehydes bearing different substituents at various positions was also investigated. To our delight, aldehydes with electron-donating groups such as −O^*i*^Pr (**3r**), −OMe (**3q**), −*tert*-butyl (**3s**), or −Me (**3t**), halogens such as −F (**3x**), as well as mildly electron-withdrawing groups such as −Cl (**3w**), provided the desired products in 49–70% isolated yields. Notably, a range of ortho-substituted aldehydes could also be used as effective coupling partners. For example, the presence of −OMe (**3p**), −Me (**3u**), and even −Cl (**3v**) at the ortho position was well tolerated, affording the desired products in 49–65% yields. However, benzaldehydes bearing strongly electron-withdrawing groups, such as −CF_3_, gave the desired products but only with limited efficiency (cf. Figure S7 for the current limitations of the synthetic scope).

Finally, we explored substrates bearing different functional groups at various positions on both the aldehyde and the alkyne. A diverse set of functionalized indanones could be synthesized using this protocol. For example, the presence of a *tert*-butyl substituent at the para position of both reaction partners afforded the desired product **3y** in 63% yield. Similarly, product **3aa**, bearing an electron-donating group para to the aldehyde and meta to the alkyne, was obtained in 50% isolated yield. In other examples, a dimethyl-substituted aldehyde, in which one of the methyl groups was located ortho to the new bond-forming position, reacted smoothly. For instance, when 1-(*tert*-butyl)-4-ethynylbenzene and 1-ethynyl-3,5-difluorobenzene were used as alkyne partners, the desired products **3z** and **3ac** were isolated in 67% and 75% yields, respectively. To our delight, compound **3ae**, featuring an −OMe group ortho to the aldehyde, successfully coupled with a Bpin-substituted alkyne, affording the corresponding product in 68% yield.

To gain insight into the reaction mechanism, a series of control and labeling experiments were conducted. First, when 2,2,6,6-tetramethylpiperidine 1-oxyl (TEMPO) was added as a radical scavenger, the desired indanone product was not observed. Instead, the formation of the acyl–TEMPO adduct, 2,2,6,6-tetramethylpiperidin-1-yl benzoate (compound **4**, in Figure 2A), was detected indicating that the reaction proceeds via a radical pathway (in this case, via the formation of the acyl radical).

**Figure 2:**
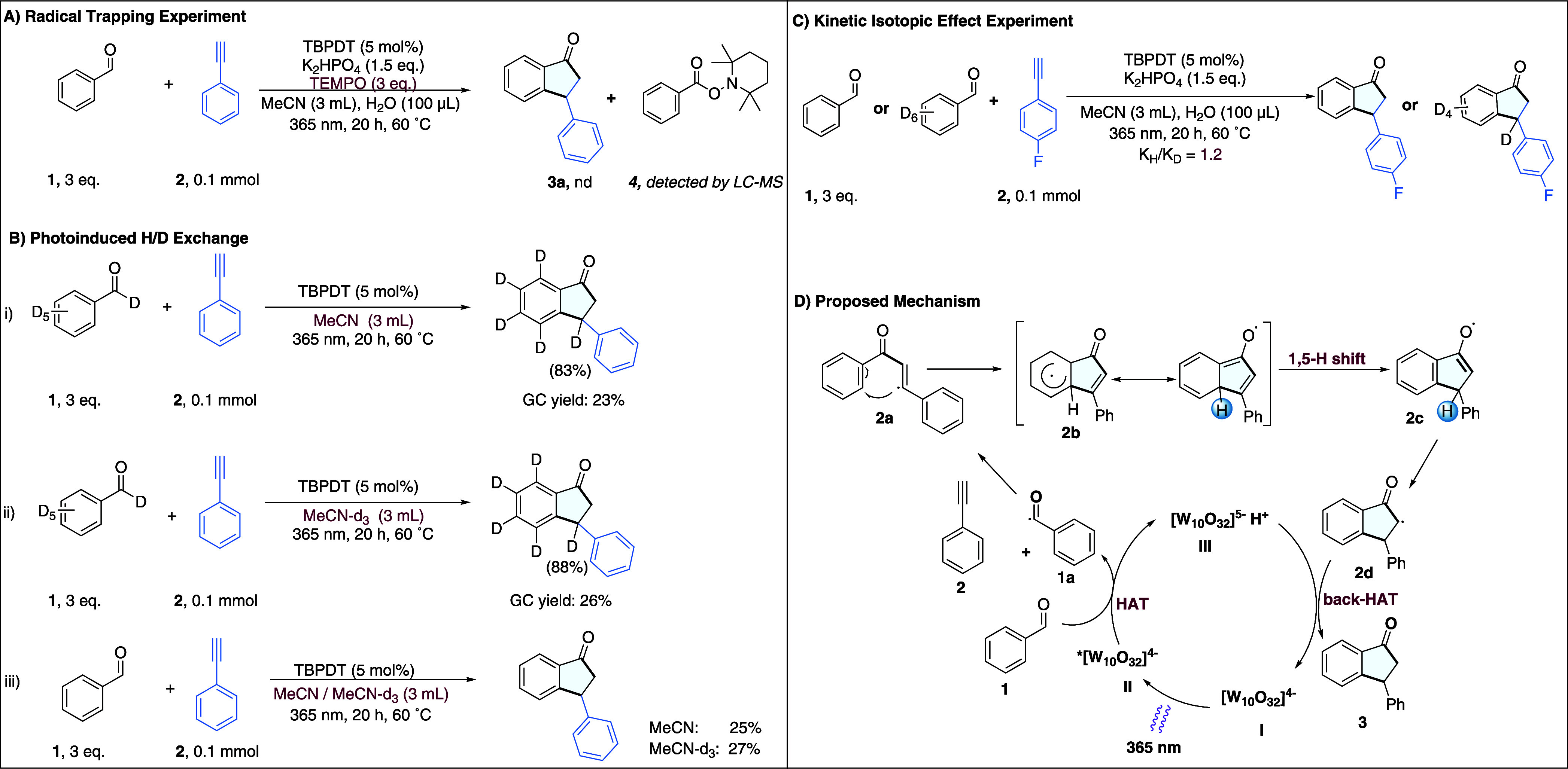
Mechanistic investigation a) Radical trapping experiment with TEMPO; b) Photoinduced H/D exchange; c) Kinetic isotopic effect; d) Proposed Mechanism.

Next, photochemical reactions were conducted using benzaldehyde-d_6_ and phenylacetylene in both MeCN and MeCN-*d*_3_ (Figure 2B, entries i and ii). Deuterium incorporation was observed in both cases at the benzylic position in the product. Since benzaldehyde-d_6_ was the sole deuterium source in nondeuterated MeCN, this observation supports the involvement of a 1,5-hydrogen (or in this case, deuterium) shift mechanism,^27^ along with a back-hydrogen atom transfer (back-HAT) from the decatungstate species (H^+^ [W_10_O_32_]^5–^) to regenerate the ground-state photocatalyst [W_10_O_32_]^4–^.^27,33,34^ As a control reaction (Figure 2B, entry iii) when the reaction was performed using benzaldehyde in MeCN and MeCN-*d*_3_, a comparable yield was obtained, suggesting that the solvent does not directly participate in the process.

Finally, a kinetic isotope effect (KIE) study was performed using benzaldehyde and benzaldehyde-d_6_. A K_H_/K_D_ value of 1.2 was observed (Figure 2C), suggesting that C–H (or C–D) bond cleavage at the aldehyde is not the rate-determining step.^35^ This implies that other steps, likely the radical cyclization, are slower and more kinetically significant.

We note that the desired product was obtained even when only K_2_HPO_4_ was used as a base/additive in the absence of the photocatalyst (cf. entry 5, Table 1), albeit in lower yield (see Scheme S2 for the proposed mechanism of the photocatalyst-free pathway). This suggests that the acyl radical formation can also occur under photocatalyst-free conditions, providing an additional alternative route for product formation. Although a comprehensive mechanistic pathway under these conditions remains unclear, benzaldehyde is known to undergo photoreaction at 365 nm and can therefore be directly excited.^36^ Upon excitation, species **II** (cf. see Scheme S2) can abstract a hydrogen atom from aldehyde **1**, generating the corresponding acyl radical,^36^ which subsequently reacts with phenylacetylene under the photochemical conditions to form **2a**. In the absence of both TBPDT and K_2_HPO_4_, only 17% of the desired product was obtained, highlighting the role of K_2_HPO_4_ in facilitating product formation, which is further enhanced by TBPDT when employed as the photocatalyst. Furthermore, UV–Vis analysis (Figure S4) shows changes in the aldehyde absorption spectrum, suggesting weak noncovalent interactions with the substrate. K_2_HPO_4_ likely modulates the electronic environment of the substrates, potentially enhancing the efficiency of benzaldehyde photoexcitationor stabilizing reaction intermediates, thereby enabling the formation of the desired product in improved yields.

Based on our experimental results and prior literature,^27,34^ the proposed mechanism for the photochemical reaction in the presence of TBPDT is depicted in Figure 2D. Upon irradiation, the decatungstate anion [W_10_O_32_]^4–^ (**I**) undergoes intersystem crossing to generate a long-lived triplet excited state, *­[W_10_O_32_]^4–^ (**II**). This excited photocatalyst abstracts a hydrogen atom from the aldehyde, forming an acyl radical (**1a**) and the reduced decatungstate species H^+^ [W_10_O_32_]^5–^ (**III**). The acyl radical then adds to phenylacetylene (**2**), affording a vinyl radical intermediate (**2a**), which undergoes intramolecular radical addition onto the aryl ring to yield a cyclized intermediate (**2b**). A subsequent 1,5-hydrogen shift restores aromaticity, producing intermediate **2c**. Catalyst turnover occurs via back-HAT from H^+^ [W_10_O_32_]^5–^ to **2d**, delivering the indanone product **3** and regenerating the ground-state catalyst [W_10_O_32_]^4–^ . This catalytic cycle underscores the crucial role of TBPDT in both the generation of radical species and the final reduction step required for product formation.

## Conclusions

In conclusion, we report here a photochemical C–H annulation strategy for the synthesis of diverse indanone derivatives from simple aromatic aldehydes and terminal alkynes, using tetrabutylphosphonium decatungstate (TBPDT) as a hydrogen atom transfer photocatalyst. This metal-free transformation proceeds under mild conditions, employs readily available and inexpensive substrates, and does not require decarboxylative pathways, prefunctionalization, or the use of protecting groups. The method demonstrates broad substrate tolerance and offers a sustainable, streamlined alternative for indanone synthesis. We believe this approach significantly advances the toolbox for direct indanone construction and opens new avenues for further exploration in radical-mediated annulation chemistry.

## Supplementary Material



## Data Availability

The data underlying this study are available in the published article and its online Supporting Information.

## Notes

General information, synthetic procedures, general experimental procedure, optimization of reaction conditions, mechanistic studies, scale-up reaction, characterization data, and NMR spectra (PDF)

## #

Nanotechnology Centre, Centre for Energy and Environmental Technologies, VŠB-Technical University of Ostrava, 708 00 Ostrava-Poruba, Czech Republic
