# Supplementary material for: Photo-Catalyzed Synthesis of Indanones from Aromatic Aldehydes and Terminal Alkynes
Source: J Org Chem. 2025 Sep 22;90(39):13885–90. doi: 10.1021/acs.joc.5c01749 (PMC12501931; doi:10.1021/acs.joc.5c01749)

Supporting Information

Photo-Catalyzed Synthesis of Indanones from Aromatic Aldehydes and Terminal Alkynes

Florence Babawale[1], Indrajit Ghosh[1,2]*, Burkhard König[1]*

[1]Fakultät für Chemie und Pharmazie, Universität Regensburg, 93040 Regensburg, Germany.

[2]Current affiliation: Nanotechnology Centre, Centre for Energy and Environmental Technologies, VŠB–Technical University of Ostrava, 708 00 Ostrava-Poruba, Czech Republic.

Future correspondence may be addressed to: Indrajit Ghosh (indrajit1.ghosh@ur.de) or Burkhard König (burkhard.koenig@ur.de)

Electronic Supplementary Information

Photo-Catalyzed Synthesis of Indanones from Aromatic Aldehydes and Terminal Alkynes

Florence Babawale[1], Indrajit Ghosh[1,2]*, Burkhard König[1]*

[1]Fakultät für Chemie und Pharmazie, Universität Regensburg, 93040 Regensburg, Germany.

[2]Current affiliation: Nanotechnology Centre, Centre for Energy and Environmental Technologies, VŠB–Technical University of Ostrava, 708 00 Ostrava-Poruba, Czech Republic.

Future correspondence may be addressed to: Indrajit Ghosh (indrajit1.ghosh@ur.de) or Burkhard König (burkhard.koenig@ur.de)

The primary research data is provided under URL: https://radar4chem.radar-service.eu/radar/en/dataset/7x882f235pmxprce?token=HijZPSydhqqFnVcxONjt

Table of Contents

General Information 4

Synthetic Procedures 5

Photochemical Reaction Set Up 6

Reaction optimization 7

Mechanistic studies 10

Limitations 14

Proposed Mechanism for Catalyst-free Pathway 15

Large scale reaction 16

Substrate scope characterization data 17

References 31

NMR Spectra 32

General Information

All required fine chemicals were purchased from commercial suppliers (abcr, Acros, Alfa Aesar, Fluka, Fluorochem, Merck, Sigma Aldrich, TCI) and were used without purification unless stated otherwise. Benzaldehyde and phenylacetylene were purchased from Sigma-Aldrich and used without further purification unless otherwise noted. All air and moisture-sensitive reactions were carried out under a nitrogen atmosphere using the standard Schlenk manifold technique. Extra dry anhydrous acetonitrile was purchased from Acros organics. CeliteR 512 *medium* was used for filtration. Percentage yields refer to the isolated product after purification, unless stated otherwise. Anhydrous $MgSO_4$ was used as the drying agent unless otherwise specified.

Thin Layer Chromatography

Reactions were monitored by thin layer chromatography (TLC) analysis. This was done with silica gel pre-coated aluminum sheets (ALUGRAM Xtra SIL G UV254 Ref. 818333) and visualized using UV light (254 nm).

Automated Column Chromatography

Silica gel 60 M (0.040-0.063 mm, 230-440 mesh) was used on a Biotage Isolera Spektra. The mobile phase used for all the reactions were ethyl acetate (EtOAc) and petroleum ether (PE) (EtOAc / petroleum ether 0-25% gradient) unless stated otherwise. Solvents were distilled before use.

NMR analysis

Products were characterized by ^{1}H-NMR, ^{13}C-NMR and ^{19}F-NMR using Bruker Avance 300 or 400 MHz in $CDCl_3$ or DMSO-d_6 at room temperature. For 400 MHz = (^{1}H = 400 MHz, ^{13}C = 101 MHz, and ^{19}F = 377 MHz). All chemical shifts are quoted as parts per million [ppm] relative to residual solvent peaks. The residual solvent peaks of $CDCl_3$ were referenced to 7.26 ppm for ^{1}H-NMR, and 77.16 ppm for ^{13}C-NMR, respectively. ^{1}H-NMR signal multiplicity description of peaks are as follows: s=singlet, d=doublet, t = triplet, and m = multiplet.

GC-FID and GC-MS

GC-FID analytics were performed on a 7890B Gas Chromatography (GC) System from Agilent Technologies, with an oven temperature of 40 ^{o}C. GC-MS measurements were performed on a 7890A GC system from Agilent Technologies with an Agilent 5975 MSD Detector.

HR-MS

All compounds were also characterised and confirmed by HR-MS, which was measured at the Central Analytical Laboratory of the University of Regensburg. The spectra were measured on a Jeol AccuTOF GCX, Agilent Q-TOF 6540 UHD, Finnigan MAT SSQ 710 A, or a ThermoQuest Finnigan TSQ 7000 instrument.

Synthetic Procedures

Synthesis of decatungstate catalysts

Preparation of tetrabutylphosphonium decatungstate (TBPDT)

TBPDT was synthesized using a method known from the literature.[1] Sodium tungstate dihydrate (12.8 g, 38.8 mmol, 2.5 equiv.) and 80 mL deionized water were added to 250 mL conical flask with stir bar. This solution was heated to 75 °C, and acidified with 26.8 mL of 3 M aqueous HCl. The acidified solution was then heated at 90 °C for 10 minutes. Afterwards, a solution of tetrabutylphosphonium bromide (5.26 g, 15.5 mmol, 0.4 equiv.) in 8 mL deionized water was added in one portion. A cream precipitate was formed immediately upon addition of the phosphonium bromide. The solution was stirred for an additional 5 minutes. The reaction mixture was cooled to room temperature and the white solid was filtered off on a Buchner funnel. The resulting solid was rinsed with water, frozen, and then dried via lyophilization to remove residual moisture overnight. The resulting solids were then recrystallized from minimal hot acetone, TBPDT was obtained as a white solid (5.34 g, 1.58 mmol, 41% yield). Purity was determined by UV-Vis spectroscopy. Electroanalytical characterisation is consistent with literature data.[1]

Preparation of tetra-*n*-butylammonium decatungstate (TBADT)

TBADT was synthesized using a known method from the literature.[2] Tetrabutylammonium bromide (2.4 g, 7.40 mmol, 1.0 equiv.) and sodium tungstate dihydrate $Na_2WO_4 \cdot 2H_2O$ (5.0 g, 15.2 mmol, 2.0 equiv.) were given in separate Erlenmeyer flasks. Deionised H_2O (150 mL) was added to each flask. Both solutions were rapidly stirred and heated to 90 °C. When both solutions reached 90 °C (checked by thermometers), concentrated HCl was added to each solution until their pH was stabilised at 2. The solutions were then combined and stirred at 90 °C for 30 min. A white suspension of TBADT is formed. The reaction mixture was cooled to room temperature and the white solid was filtered off on a Buchner funnel. After washing with water, the resulting solid was frozen, and then dried via lyophilization to remove residual moisture for 16 h. The solid was then suspended in dichloromethane (90 mL) and stirred at room temperature for 4 h. After filtration through a Buchner funnel, TBADT was obtained as a white solid (4.2 g, 1.27 mmol, 83%). Electroanalytical characterisation is consistent with literature data.[2]

Photochemical Reaction Set Up

Small scale reaction at 60 °C

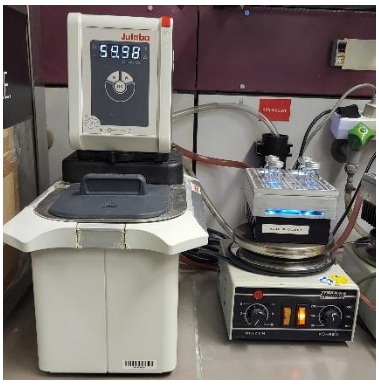

Figure S1. Photochemical reactions were conducted in sealed 10 mL glass vials and irradiated with 6 x LED Engine LZ4-40UB00-00U5. λ = 365 nm (3 W, 700 mA). The irradiation setup was connected to a thermostat.

Small scale reaction at 90 °C

Photochemical reactions were conducted in sealed 10 mL schlenk tube and irradiated with 1 x LED Engine LZ4-40UB00-00U5. λ = 365 nm (3 W, 700 mA).

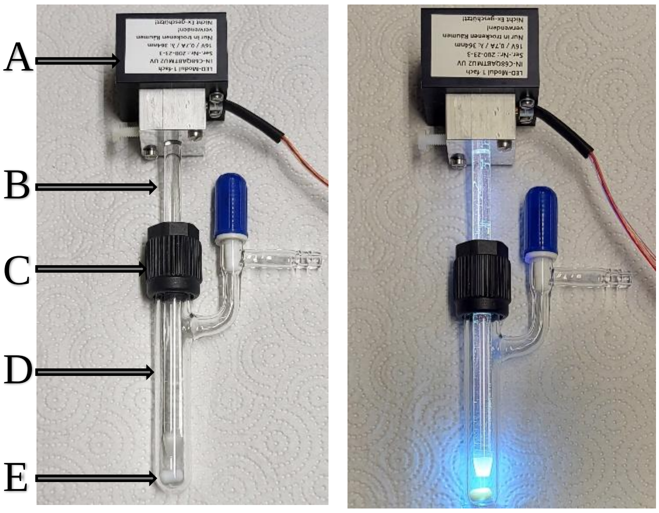

Figure S2. Irradiation setup for photochemical reactions: A) LED, B) glass rod, C) Teflon adapter, D) Schlenk tube, and E) PTFE-coated magnetic stirring bar.

Reaction optimization

General Procedure A: Photochemical reaction at 60 °C

To a 10 mL crimp vial with a stirring bar, C-H nucleophile (0.3 mmol, 3 equiv.), phenylacetylene derivatives (0.1 mmol, 1 equiv.), dipotassium phosphate (26.13 mg, 0.15 mmol, 1.5 equiv.), tetrabutylphosphonium decatungstate (16.9 mg, 0.005 mmol, 5 mol%), anhydrous CH_3CN (3 mL), and water (0.1 mL) were added. The reaction vial cap was crimped, and the reacting mixture degassed three times using the freeze-pump-thaw method and then back-filled with nitrogen gas. The reaction vial was then wrapped with parafilm, stirred, and irradiated with 365 nm (3 W) LEDs for 20 h at 60 ºC (Figure S1). The reaction progress was monitored by GC-FID and GC-MS.

General Procedure B: Photochemical reaction at 90 °C

To a 10 mL schlenk tube with a stirring bar, Aldehyde derviates as the C-H nucleophile (0.3 mmol, 3 equiv.), phenylacetylene derivatives as the micheal acceptor (0.1 mmol, 1 equiv.), dipotassium phosphate (26.13 mg, 0.15 mmol, 1.5 equiv.), tetrabutylphosphonium decatungstate (16.9 mg, 0.005 mmol, 5 mol%), anhydrous CH_3CN (3 mL), and water (0.1 mL) were added. A glass rod with the Teflon adaptor is placed inside the schlenk tube and was tightly sealed. The reacting mixture was degassed three times using the freeze-pump-thaw method and then back-filled with nitrogen gas. The single 365 nm (3 W) LED was attached to the glass rod and the reaction schlenk tube was irradiated in an oil bath for 20 h at 90 ºC (Figure S2 and S3).The reaction progress was monitored by GC-FID and GC-MS. For isolation, the reaction mixture was extracted with ethylacetate, washed with brine, dried and concentrated in *vacuo*. The residue was purified by column chromatography using the solvent mixtures indicated in the General Information section. The purity was assessed by ^{1}H, ^{13}C and ^{19}F NMR in $CDCl_3$.

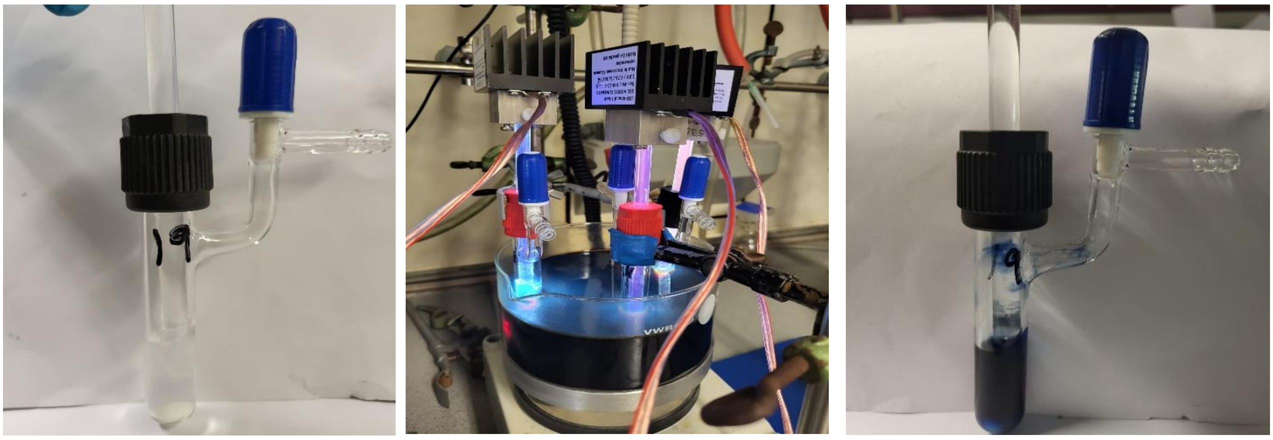

Figure S3. Photochemical reactions setup. a) Before irradiation; b) irradiation in a sealed 10 mL schlenk tube in an oil bath; c) after irradiation.

Table S1. Screening of parameters at 60 °C

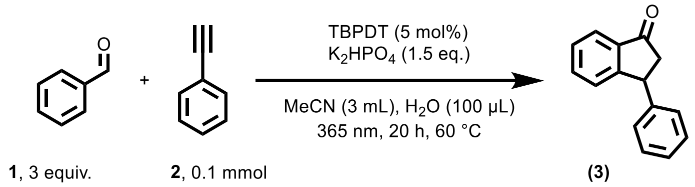

Entry	Deviation from the standard reaction condition	3 (% yield)[a]
1	None	48
**Control experiments**		
2[b]	No water	25
3	No K_2HPO_4	30
4	No TBPDT	26
5	No TBPDT, No K_2HPO_4	9
6	No light	0
**Catalysts**		
7	TBADT	44
8	10 mol % Benzophenone	33
9	10 mol % Xanthone	34
10[c]	Neutral Eosin Y (10 mol%)	n.d
11[c]	Eosin Y disodium salt (10 mol%)	n.d
12[c]	4CzIPN (5 mol%), Quinuclidine (20 mol%)	n.d
**Bases**		
13	2,6 Lutidine	40
14	Na_2CO_3	30
15	DABCO	5
16	Cs_2CO_3	13
**Equivalence of Base**		
17	1 equiv. of K_2HPO_4	43
18	3 equiv. of K_2HPO_4	46
**Equivalence of Aldehyde**		
19	2 equiv. of aldehyde	36
20	5 equiv. of aldehyde	39
**Temperature**		
21	25 °C	30
**Concentration**		
22	2 mL	23
23	4 mL	29
**Solvents**		
24	Acetone	50
25	Ethyl acetate	42
26	Trifluorotoluene	31
27	Toluene	11
28	DCM	20
**LED**		
29	395 nm	20

[a]Yields were determined by GC-FID using 1,3,5-trimethoxybenzene as an internal standard. The reactions were run under nitrogen atmosphere unless stated otherwise. [b]Without base. [c]450 nm. n.d = not detected.

Table S2. Investigating reactivity in a catalyst free condition at 60 °C**.**

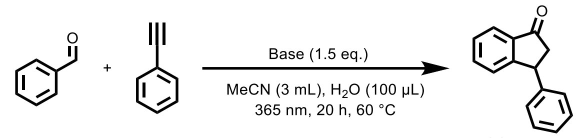

Entry	Base	3 (% yield)[a]
1	K_2HPO_4	26
2[b]	K_2HPO_4	18
3	K_2HPO_4 (w/o H_2O)	20
4	K_2HPO_4 (3 eq.)	30
5	Na_2CO_3	32
6	Na_2CO_3 (3 eq.)	28
7	K_2CO_3	23
8	K_2CO_3 (3 eq.)	27
9	2,6 Lutidine	n.d
10	KOtBu	n.d
11	Et_3N	n.d
12	DBU	n.d
13	DABCO	n.d
14	Cs_2CO_3	n.d

[a]Yields were determined by GC-FID using 1,3,5-trimethoxybenzene as an internal standard. The reactions were run under nitrogen atmosphere unless stated otherwise. [b]Room temperature. n.d = not detected.

Table S3. Screening of parameters at 90 °C

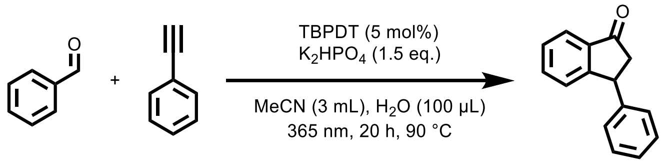

Entry	Deviation from the standard reaction condition	3 (% yield)[a]
1	10 mol % Xanthone	60
2	1 equiv. of K_2HPO_4	66
3	3 equiv. of K_2HPO_4	71
4	2 equiv. of aldehyde	60
5	5 equiv. of aldehyde	65

[a]Yields were determined by GC-FID using 1,3,5-trimethoxybenzene as an internal standard. The reactions were run under nitrogen atmosphere unless stated otherwise. An oil bath was used as the heat source.

Mechanistic studies

Photoinduced H/D exchange

To a 10 mL crimp vial with a stirring bar, benzaldehyde (30.6 µL, 0.3 mmol, 3 equiv.) or benzaldehyde-d_6 (30.5 µL, 0.3 mmol, 3 equiv.), phenylacetylene derivatives (10.9 µL, 0.1 mmol, 1 equiv.), tetrabutylphosphonium decatungstate (16.9 mg, 0.005 mmol, 5 mol%), anhydrous CH_3CN (3 mL) or deuterated CH_3CN (3 mL) were added. The reaction vial cap was crimped, and the reacting mixture was degassed three times using the freeze-pump-thaw method and then back-filled with nitrogen gas. The reaction vial was stirred, and irradiated with 365 nm (3 W) LEDs for 20 h at 60 °C. The crude mixture was analysed with both NMR and GC-FID using 1,3,5-trimethoxybenzene as an internal standard. The NMR yield was determined using the benzylic proton. For Entry 3 and 4, the actual yield of the compound was determined by GC-FID.

Table S4: Mechanistic investigation Photoinduced H/D exchange

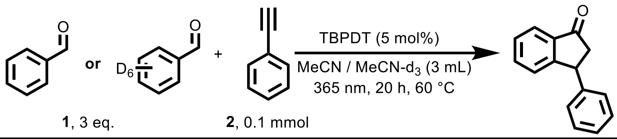

Entry	Deviation from the standard condition	3 (% yield)[a]
1	Benzaldehyde, MeCN	25
2	Benzaldehyde, MeCN-d_3	27
3	Benzaldehyde-d_6, MeCN	4
4	Benzaldehyde-d_6, MeCN-d_3	3

[a]Yields were determined by ^{1}H-NMR using 1,3,5-trimethoxybenzene as an internal standard. The NMR yield was determined using the benzylic proton.

UV-Vis analysis Measurement

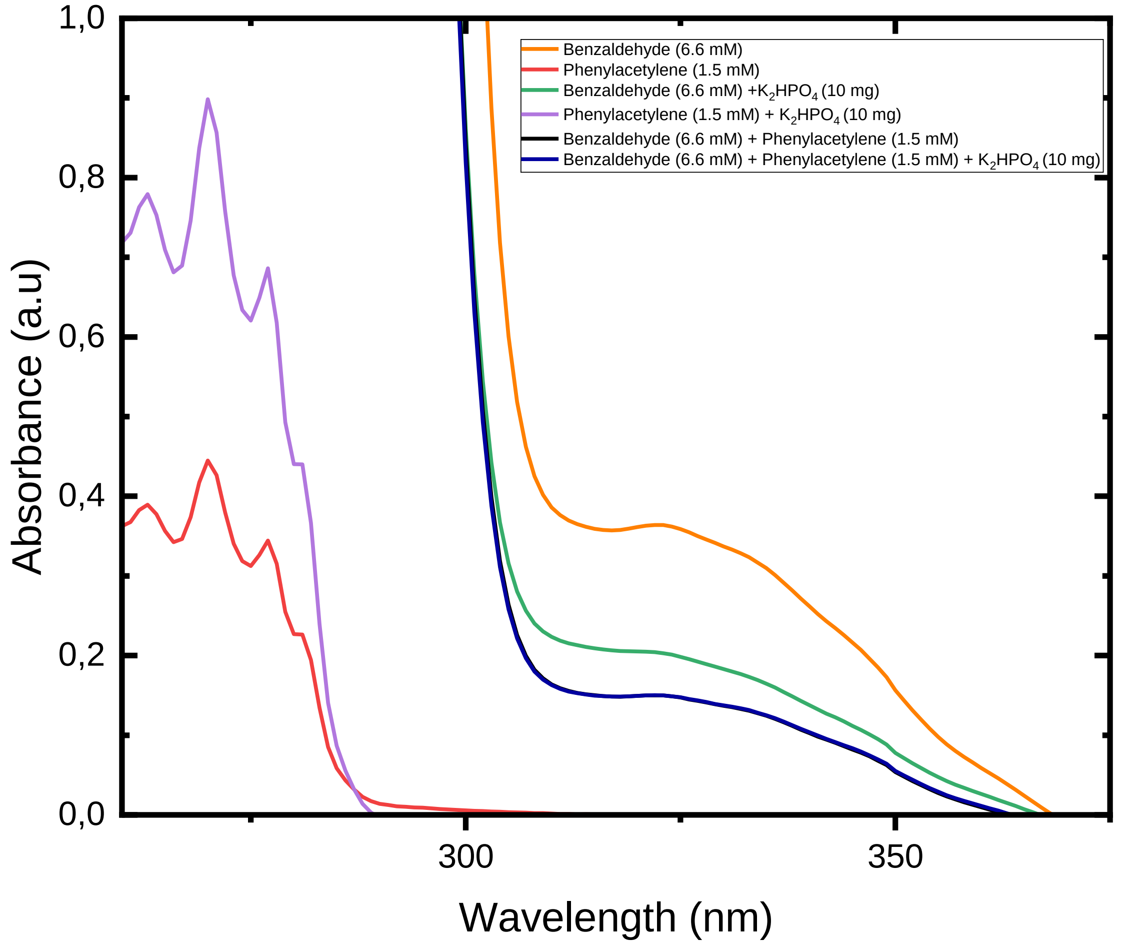

Figure S4. The analysis was carried out using a solvent mixture consisting of 3 mL of acetonitrile and 0.1 mL of water. To this mixture, 2 µL of benzaldehyde and 0.5 µL of phenylacetylene were added.

The UV-Vis studies revealed that the addition of K_2HPO_4 induces a slight hypochromic effect in benzaldehyde, while phenylacetylene exhibits a significant hyperchromic response. This suggests that K_2HPO_4 likely modulates the electronic environments of the substrates through weak non-covalent interactions. Consequently, such base-induced electronic tuning may influence the substrates reactivity under 365 nm irradiation.

Reaction with TEMPO

To a 10 mL crimp vial with a stirring bar, benzaldehyde (30.6 µL, 0.3 mmol, 3 equiv.), phenylacetylene derivatives (10.9 µL, 0.1 mmol, 1 equiv.), dipotassium phosphate (26.13 mg, 0.15 mmol, 1.5 equiv.), tetrabutylphosphonium decatungstate (16.9 mg, 0.005 mmol, 5 mol%), TEMPO (46.9 mg, 0.3 mmol, 3 equiv.), anhydrous CH_3CN (3 mL), and water (0.1 mL) were added. The reaction vial cap was crimped, and the reacting mixture was degassed three times using the freeze-pump-thaw method and then back-filled with nitrogen gas. The reaction vial was stirred, and irradiated with 365 nm (3 W) LEDs for 20 h at 60 °C. The mixture was analysed using LC-MS. Complete inhibition of **3a** was observed thereby indicating a radical pathway.

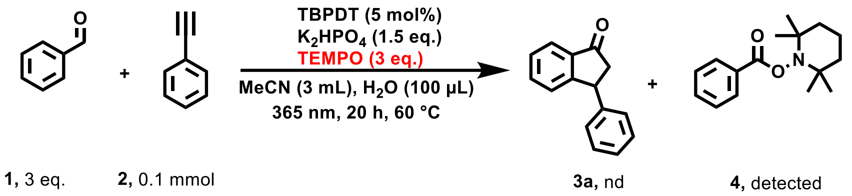

Scheme S1. Radical trapping with TEMPO

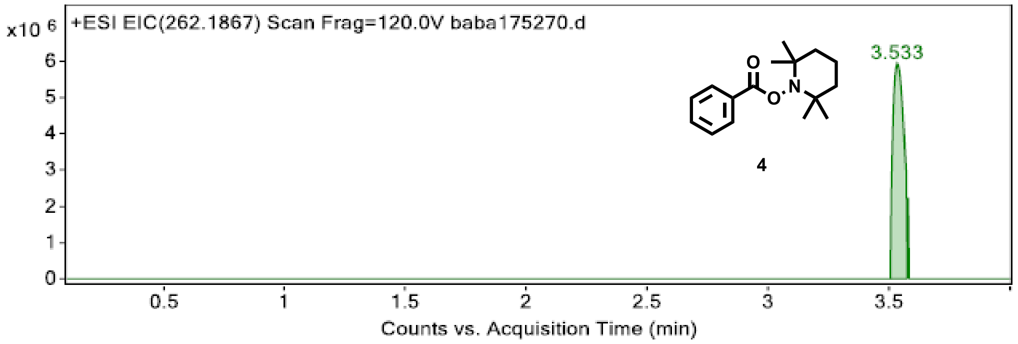

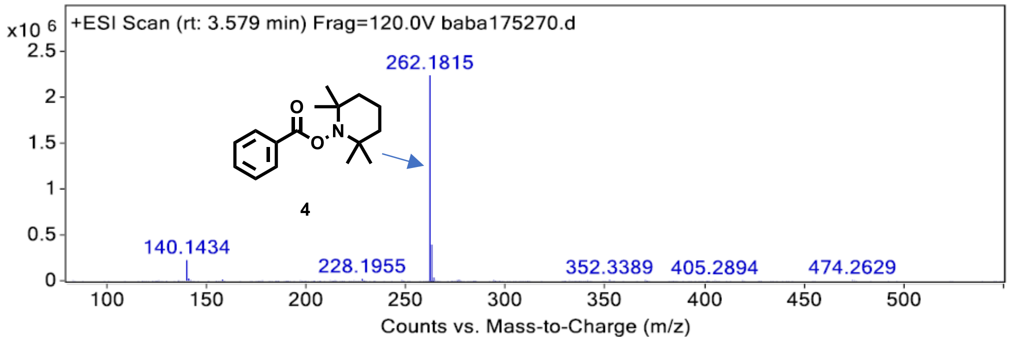

Figure S5. Ion chromatograph of crude mixture showing ion mass of **4**.

Kinetic isotopic effect (KIE) measurement

To a 10 mL crimp vial with a stirring bar, benzaldehyde (30.6 µL, 0.3 mmol, 3 equiv.) or benzaldehyde-d_6 (30.6 µL, 0.3 mmol, 3 equiv.), phenylacetylene derivatives (10.9 µL, 0.1 mmol, 1 equiv.), dipotassium phosphate (26.13 mg, 0.15 mmol, 1.5 equiv.), tetrabutylphosphonium decatungstate (16.9 mg, 0.005 mmol, 5 mol%), anhydrous CH_3CN (3 mL), and water (0.1 mL) were added. The reaction vial cap was crimped, and the reacting mixture was degassed three times using the freeze-pump-thaw method and then back-filled with nitrogen gas. The reaction vial was stirred, and irradiated with 365 nm (3 W) LEDs for 20 h at 60 ºC. Six independent runs were conducted for both benzaldehyde and benzaldehyde-d_6. The mixture was analysed using ^{19}F-NMR using trifluorotoluene as internal standard and the values were used to calculate the KIE.

Table S6. Kinetic isotopic effect (KIE) measurement

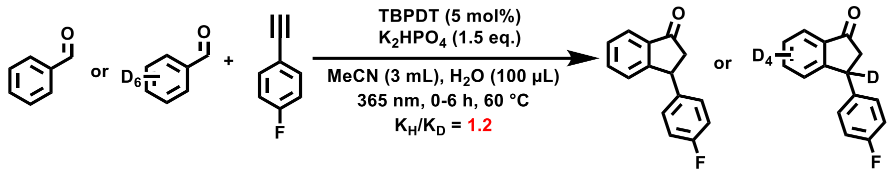

Entry	Time (h)	Yield of 3i (%)	Yield of 5 (%)
**1**	0	0	0
**2**	1	7	5
**3**	2	14	9
**4**	4	22	17
**5**	5	29	19
**6**	6	34	29

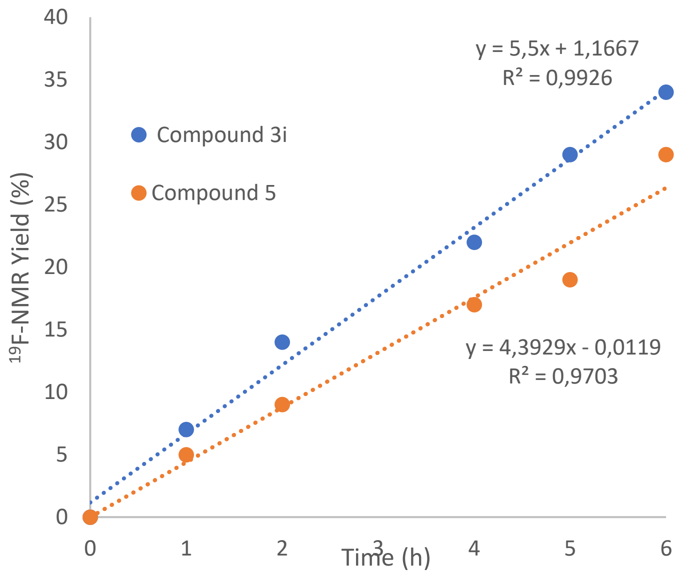

Figure S6. Kinetic isotopic effect measurement with **3i** and **5**.

Limitations

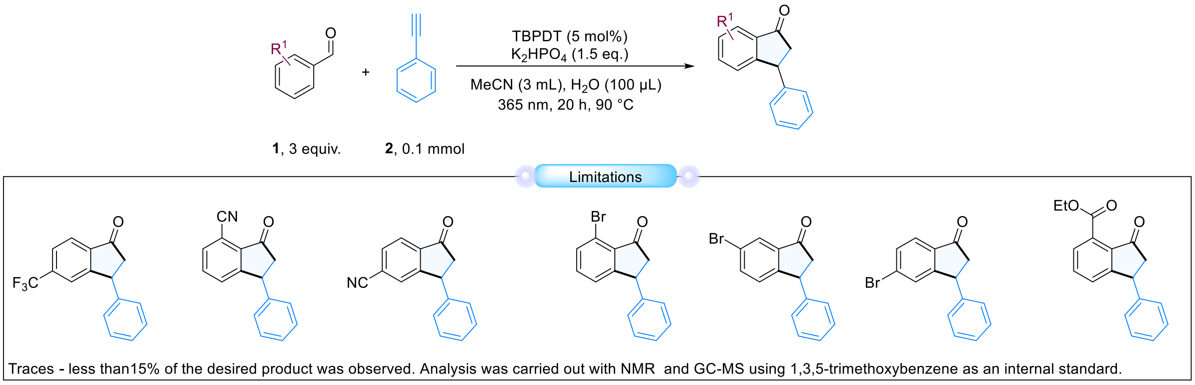

Figure S7. Examples highlighting the limitations encountered with benzaldehyde substrates under the reported reaction conditions.

Proposed Mechanism for Catalyst-free Pathway

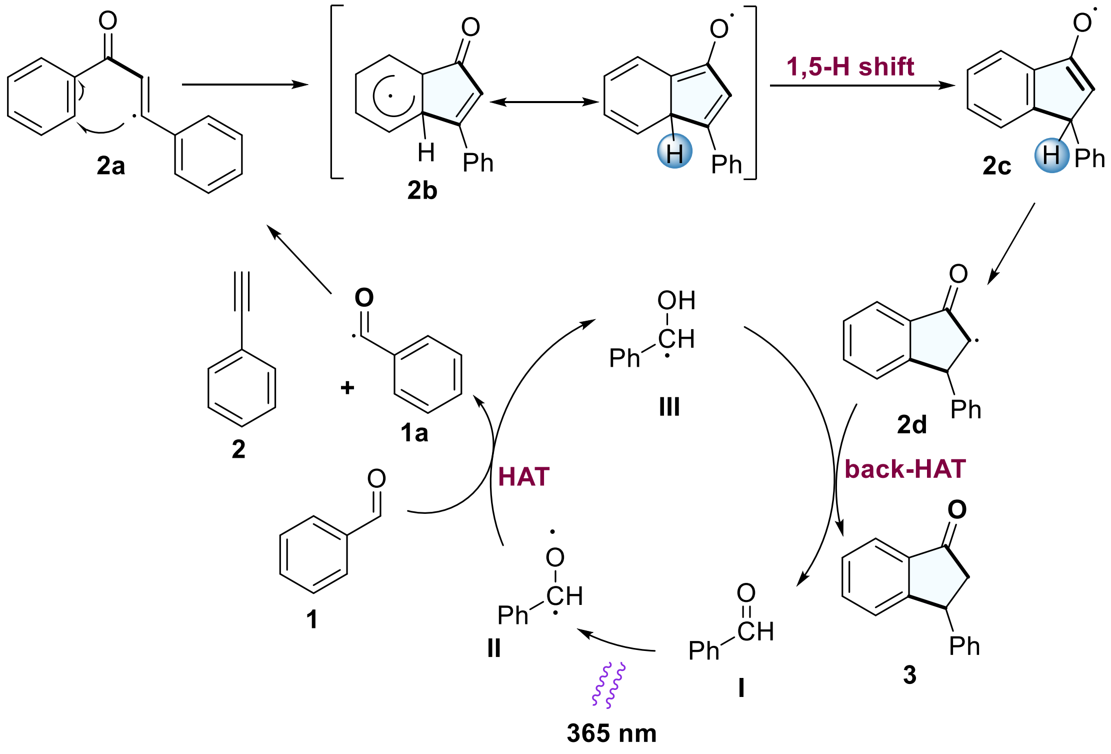

Scheme S2. Proposed mechanism for catalyst-free pathway

Benzaldehyde is known to undergo photoreaction at 365 nm and can therefore be directly excited. Upon excitation, species **II** can abstract a hydrogen from aldehyde **1**, generating the corresponding acyl radical, which reacts with phenylacetylene to form **2a**. This intermediate undergoes intramolecular cyclization to yield **2b**, followed by a 1,5-hydrogen shift to form **2c**. Back-HAT from species **III** then delivers the desired product.

Large scale reaction

A large scale reaction at 90 °C for compound 3a.

To a 500 mL three-neck round-bottom flask equipped with a magnetic stirring bar were added benzaldehyde (3.0 mL, 30.0 mmol, 3.0 equiv), phenylacetylene (1.10 mL, 10.0 mmol, 1.0 equiv), dipotassium phosphate (3.48 g, 20.0 mmol, 2.0 equiv), tetrabutylphosphonium decatungstate (1.69 g, 0.50 mmol, 5 mol%), anhydrous CH_3CN (200 mL), and water (5 mL). The flask was fitted with a condenser, degassed three times and back-filled with nitrogen gas. A nitrogen balloon was attached to the top of the condenser to maintain the inert atmosphere throughout the reaction.

The reaction mixture was heated to 90 °C in an oil bath and irradiated with three 370 nm, 40 W PR160L Kessil lamp at maximum intensity (Figure S8). The reaction progress was monitored by TLC and GC–FID. After 24 h, an additional portion of benzaldehyde (3.0 mL, 30.0 mmol, 3 equiv), was added to facilitate consumption of the limiting reagent, phenylacetylene. The total reaction time was 48 h.

For isolation, the reaction mixture was extracted with ethylacetate, washed with brine, dried, and concentrated in *vacuo*. The crude residue was purified by column chromatography using the solvent systems described in the General Information section. The purity of the final product was confirmed by ^{1}H and ^{13}C NMR spectroscopy ($CDCl_3$).

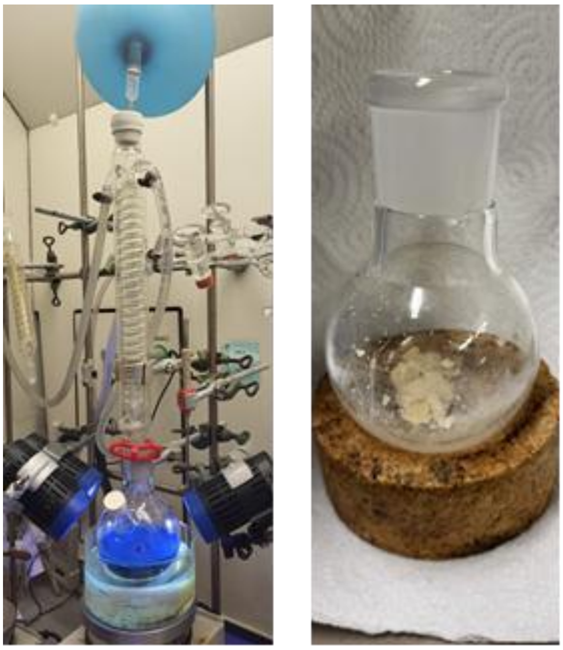

Figure S8. Pictures showing the photochemical reactions setup of the gram scale synthesis. a) irradiation setup; b) Compound **3a** after purification.

Substrate scope characterization data

3-phenyl-2,3-dihydro-1H-inden-1-one (3a)[3]

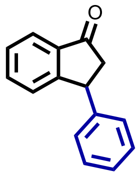

Compound **3a** was synthesized using general procedure B from benzaldehyde (30.6 µL, 0.3 mmol, 3 equiv.) and phenylacetylene (10.9 µL, 0.1 mmol, 1 equiv.) The reaction was performed three times, combined and isolated. Purification via column chromatography using petroleum ether/ethyl acetate solvent mixture as an eluent yielded **3a** as a cream solid in 69% yield (43.3 mg).

Gram scale reaction: The compound was prepared using benzaldehyde (6.0 mL, 60.0 mmol, 6 equiv), phenylacetylene (1.10 mL, 10.0 mmol, 1 equiv), dipotassium phosphate (3.48 g, 20.0 mmol, 2.0 equiv), tetrabutylphosphonium decatungstate (1.69 g, 0.50 mmol, 5 mol %), anhydrous CH_3CN (200 mL), and water (5 mL). The reaction mixture was irradiated under N_2 at 90 °C for 48 h using three 370 nm, 40 W PR160L Kessil lamp. The product was purified following the general procedure via column chromatography on silica gel using petroleum ether/ethyl acetate solvent mixture as an eluent and obtained as a cream soild in 44 % yield (0.93 g).

^{1}H NMR (400 MHz, CDCl$_3$) δ 7.83 (d, *J* = 7.7 Hz, 1H), 7.58 (td, *J* = 7.5, 1.3 Hz, 1H), 7.45 – 7.41 (m, 1H), 7.35 – 7.24 (m, 4H), 7.15 – 7.13 (m, 2H), 4.59 (dd, *J* = 8.1, 3.9 Hz, 1H), 3.25 (dd, *J* = 19.2, 8.1 Hz, 1H), 2.71 (dd, *J* = 19.2, 3.9 Hz, 1H). **^{13}C{^{1}H} NMR (101 MHz, CDCl$_3$)** δ 206.1, 158.0, 143.8, 136.8, 135.2, 129.0, 128.0, 127.7, 127.1, 127.0, 123.5, 46.9, 44.5. **HRMS (EI)** (m/z): $[M]^+$ calcd for $C_{15}H_{12}O$ 208.0882; found 208.0886.

3-(2-methoxyphenyl)-2,3-dihydro-1H-inden-1-one (3b)[4]

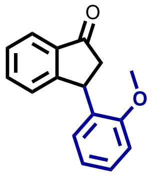

Compound **3b** was synthesized using general procedure B from benzaldehyde (30.6 µL, 0.3 mmol, 3 equiv.) and 1-ethynyl-2-methoxybenzene (12.9 µL, 0.1 mmol, 1 equiv.) The reaction was performed three times, combined and isolated. Purification via column chromatography using petroleum ether/ethyl acetate solvent mixture as an eluent yielded **3b** as a yellow oil in 45% yield (32.1 mg). **^{1}H NMR (400 MHz, CDCl$_3$)** δ 7.81 – 7.79 (m, 1H), 7.55 (td, *J* = 7.5, 1.3 Hz, 1H), 7.41 – 7.37 (m, 1H), 7.30 (dd, *J* = 7.7, 1.0 Hz, 1H), 7.24 – 7.21 (m, 1H), 6.97 – 6.95 (m, 1H), 6.90 – 6.85 (m, 2H), 4.89 (dd, *J* = 8.2, 3.6 Hz, 1H), 3.75 (s, 3H), 3.17 (dd, *J* = 19.1, 8.1 Hz, 1H), 2.69 (dd, *J* = 19.1, 3.6 Hz, 1H). **^{13}C{^{1}H} NMR (101 MHz, CDCl$_3$)** δ 206.8, 158.0, 157.4, 137.2, 134.8, 131.8, 128.5, 128.2, 127.6,

126.7, 123.4, 120.8, 111.0, 55.4, 45.3, 39.2. **HRMS (EI)** (m/z): $[M]^+$ calcd for $C_{16}H_{14}O_2$ 238.0988; found 238.0989.

3-(3-methoxyphenyl)-2,3-dihydro-1H-inden-1-one (3c) [4]

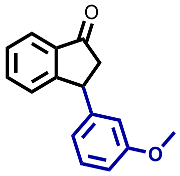

Compound **3c** was synthesized using general procedure B from benzaldehyde (30.6 µL, 0.3 mmol, 3 equiv.) and 1-ethynyl-3-methoxybenzene (12.7 µL, 0.1 mmol, 1 equiv.) The reaction was performed three times, combined and isolated. Purification via column chromatography using petroleum ether/ethyl acetate solvent mixture as an eluent yielded **3c** as a colorless oil in 58% yield (41.7 mg). **^{1}H NMR (400 MHz, CDCl$_3$)** δ 7.82 – 7.80 (m, 1H), 7.57 (td, *J* = 7.5, 1.3 Hz, 1H), 7.44 – 7.40 (m, 1H), 7.30 (dd, *J* = 7.7, 0.9 Hz, 1H), 7.23 (t, *J* = 7.9 Hz, 1H), 6.79 (ddd, *J* = 8.3, 2.6, 0.9 Hz, 1H), 6.73 – 6.70 (m, 1H), 6.66 – 6.65 (m, 1H), 4.55 (dd, *J* = 8.1, 3.8 Hz, 1H), 3.76 (s, 3H), 3.22 (dd, *J* = 19.2, 8.1 Hz, 1H), 2.70 (dd, *J* = 19.2, 3.9 Hz, 1H). **^{13}C{^{1}H} NMR (101 MHz, CDCl$_3$)** δ 206.0, 160.1, 157.8, 145.4, 136.8, 135.2, 130.0, 128.0, 127.0, 123.5, 120.1, 113.7, 112.1, 55.3, 46.8, 44.5. **HRMS (EI)** (m/z): $[M]^+$ calcd for $C_{16}H_{14}O_2$ 238.0988; found 238.0991.

3-(4-methoxyphenyl)-2,3-dihydro-1H-inden-1-one (3d) [4]

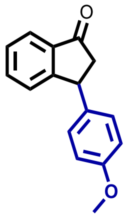

Compound **3d** was synthesized using general procedure B from benzaldehyde (30.6 µL, 0.3 mmol, 3 equiv.) and 1-ethynyl-4-methoxybenzene (13.2 mg, 0.1 mmol, 1 equiv.) The reaction was performed three times, combined and isolated. Purification via column chromatography using petroleum ether/ethyl acetate solvent mixture as an eluent yielded **3d** as a yellow oil in 53% yield (37.7 mg). **^{1}H NMR (400 MHz, CDCl$_3$)** δ 7.81 – 7.79 (m, 1H), 7.57 (td, *J* = 7.5, 1.2 Hz, 1H), 7.43 – 7.39 (m, 1H), 7.28 (d, *J* = 1.0 Hz, 1H), 7.05 – 7.03 (m, 2H), 6.86 – 6.84 (m, 2H), 4.54 (dd, *J* = 8.0, 3.9 Hz, 1H), 3.79 (s, 3H), 3.22 (dd, *J* = 19.2, 8.0 Hz, 1H), 2.65 (dd, *J* = 19.2, 3.9 Hz, 1H). **^{13}C{^{1}H} NMR (101 MHz, CDCl$_3$)** δ 206.3, 158.7, 158.4, 136.8, 135.8, 135.2, 128.7, 127.9, 126.9, 123.4, 114.4, 55.4, 47.1, 43.8. **HRMS (EI)** (m/z): $[M]^+$ calcd for $C_{16}H_{14}O_2$ 238.0988; found 238.0992.

3-(4-(tert-butyl)phenyl)-2,3-dihydro-1H-inden-1-one (3e) [4]

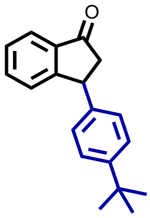

Compound **3e** was synthesized using general procedure B from benzaldehyde (30.6 µL, 0.3 mmol, 3 equiv.) and 1-(tert-butyl)-4-ethynylbenzene (18.0 µL, 0.1 mmol, 1 equiv.) The reaction was performed three times, combined and isolated. Purification via column chromatography using petroleum ether/ethyl acetate solvent mixture as an eluent yielded **3e** as a yellow oil in 63% yield (50.0 mg). **^{1}H NMR (400 MHz, $CDCl_3$)**) δ 7.82 – 7.80 (m, 1H), 7.57 (td, *J* = 7.5, 1.3 Hz, 1H), 7.43 – 7.39 (m, 1H), 7.34 – 7.29 (m, 3H), 7.07 – 7.05 (m, 2H), 4.56 (dd, *J* = 8.0, 3.9 Hz, 1H), 3.22 (dd, *J* = 19.2, 8.0 Hz, 1H), 2.70 (dd, *J* = 19.2, 3.9 Hz, 1H), 1.31 (s, 9H). **^{13}C{^{1}H} NMR (101 MHz, $CDCl_3$)** δ 206.3, 158.2, 149.9, 140.6, 135.1, 127.9, 127.3, 127.0, 125.9, 123.4, 46.9, 44.1, 34.5, 31.4. **HRMS (EI)** (m/z): $[M]^+$ calcd for $C_{19}H_{20}O$ 264.1508; found 264.1506.

3-(3-chlorophenyl)-2,3-dihydro-1H-inden-1-one (3f) [4]

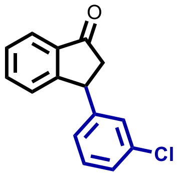

Compound **3f** was synthesized using general procedure B from benzaldehyde (30.6 µL, 0.3 mmol, 3 equiv.) and 1-chloro-3-ethynylbenzene (12.3 µL, 0.1 mmol, 1 equiv.) The reaction was performed three times, combined and isolated. Purification via column chromatography using petroleum ether/ethyl acetate solvent mixture as an eluent yielded **3f** as a colorless oil in 57% yield (41.8 mg). **^{1}H NMR (400 MHz, $CDCl_3$)** δ 7.83 – 7.81 (m, 1H), 7.60 (td, *J* = 7.5, 1.2 Hz, 1H), 7.46 – 7.42 (m, 1H), 7.29 – 7.26 (m, 1H), 7.25 – 7.23 (m, 2H), 7.12 – 7.11 (m, 1H), 7.02 – 6.99 (m, 1H), 4.56 (dd, *J* = 8.1, 3.9 Hz, 1H), 3.23 (dd, *J* = 19.2, 8.1 Hz, 1H), 2.66 (dd, *J* = 19.2, 3.9 Hz, 1H). **^{13}C{^{1}H} NMR (101 MHz, $CDCl_3$)** δ 205.4, 157.1, 145.8, 136.9, 135.4, 134.8, 130.3, 128.3, 127.9, 127.3, 126.9, 125.9, 123.7, 46.7, 44.2. **HRMS (EI)** (m/z): $[M]^+$ calcd for $C_{15}H_{11}OCl$ 242.0492; found 242.0489.

3-(4-chlorophenyl)-2,3-dihydro-1H-inden-1-one (3g) [4]

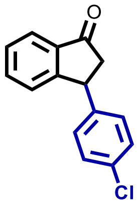

Compound **3g** was synthesized using general procedure B from benzaldehyde (30.6 µL, 0.3 mmol, 3 equiv.) and 1-chloro-4-ethynylbenzene (13.7 mg, 0.1 mmol, 1 equiv.) The reaction was performed three times, combined and isolated. Purification via column chromatography using

petroleum ether/ethyl acetate solvent mixture as an eluent yielded **3g** as a colorless oil in 41% yield (29.6 mg). **^{1}H NMR (400 MHz, CDCl$_3$)** δ 7.83 – 7.80 (m, 1H), 7.58 (td, *J* = 7.4, 1.3 Hz, 1H), 7.45 – 7.41 (m, 1H), 7.30 – 7.27 (m, 2H), 7.26 – 7.23 (m, 1H), 7.06 (d, *J* = 8.4 Hz, 2H), 4.56 (dd, *J* = 8.1, 3.9 Hz, 1H), 3.23 (dd, *J* = 19.2, 8.1 Hz, 1H), 2.63 (dd, *J* = 19.2, 3.9 Hz, 1H). **^{13}C{^{1}H} NMR (101 MHz, CDCl$_3$)** δ 205.6, 157.4, 142.3, 136.8, 135.3, 132.9, 129.2, 129.1, 128.2, 126.8, 123.6, 46.8, 43.9. **HRMS (EI)** (m/z): [M]$^+$ calcd for $C_{15}H_{11}OCl$ 242.0492; found 242.0497.

3-(2-fluorophenyl)-2,3-dihydro-1H-inden-1-one (3h)

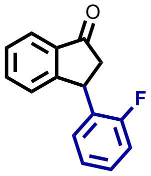

Compound **3h** was synthesized using general procedure B from benzaldehyde (30.6 µL, 0.3 mmol, 3 equiv.) and 1-ethynyl-2-fluorobenzene (11.3 µL, 0.1 mmol, 1 equiv.) The reaction was performed three times, combined and isolated. Purification via column chromatography using petroleum ether/ethyl acetate solvent mixture as an eluent yielded **3h** as a yellow oil in 58% yield (39.3 mg). **^{1}H NMR (400 MHz, CDCl$_3$)** δ 7.84 – 7.81 (m, 1H), 7.59 (td, *J* = 7.5, 1.3 Hz, 1H), 7.45 – 7.42 (m, 1H), 7.32 – 7.31 (m, 1H), 7.25 – 7.21 (m, 1H), 7.10 – 7.04 (m, 2H), 6.98 (td, *J* = 7.6, 1.7 Hz, 1H), 4.88 (dd, *J* = 8.2, 3.8 Hz, 1H), 3.24 (dd, *J* = 19.2, 8.2 Hz, 1H), 2.71 (dd, *J* = 19.1, 3.8 Hz, 1H) . **^{13}C{^{1}H} NMR (101 MHz, CDCl$_3$)** δ 205.6, 161.0 (d, *J* = 246.1 Hz), 156.7, 137.0, 135.2, 130.4 (d, *J* = 14.1 Hz), 128.9 (d, *J* = 4.1 Hz), 128.7 (d, *J* = 8.2 Hz), 128.1, 126.7, 124.6 (d, *J* = 3.7 Hz), 123.7, 115.8 (d, *J* = 22.0 Hz), 45.3, 37.7 (d, *J* = 3.0 Hz). **^{19}F NMR (377 MHz, CDCl$_3$)** δ −118.85. **HRMS (EI)** (m/z): [M]$^+$ calcd for $C_{15}H_{11}OF$ 226.0788; found 226.0788.

3-(4-fluorophenyl)-2,3-dihydro-1H-inden-1-one (3i) [3]

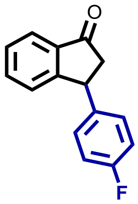

Compound **3i** was synthesized using general procedure B from benzaldehyde (30.6 µL, 0.3 mmol, 3 equiv.) and 1-ethynyl-4-fluorobenzene (12.0 mg, 0.1 mmol, 1 equiv.) The reaction was performed three times, combined and isolated. Purification via column chromatography using petroleum ether/ethyl acetate solvent mixture as an eluent yielded **3i** as a white solid in 73% yield (49.7 mg). **^{1}H NMR (400 MHz, CDCl$_3$)** δ 7.83 – 7.80 (m, 1H), 7.58 (td, *J* = 7.5, 1.3 Hz, 1H), 7.45 – 7.41 (m, 1H), 7.27 – 7.24 (m, 1H), 7.11– 7.06 (m, 2H), 7.03 – 6.97 (m, 2H), 4.57 (dd, *J* = 8.1, 3.9 Hz, 1H), 3.23 (dd, *J* = 19.2, 8.1 Hz, 1H), 2.64 (dd, *J* = 19.2, 3.9 Hz, 1H). **^{13}C{^{1}H} NMR (101 MHz, CDCl$_3$)** δ 205.8, 161.9 (d, *J* = 245.4 Hz), 157.7, 139.5 (d, *J* = 3.1 Hz), 136.8, 135.3, 129.2 (d, *J* = 8.1 Hz), 128.1, 126.9,

123.6, 115.9 (d, *J* = 21.6 Hz), 47.0, 43.8. **^{19}F NMR (377 MHz, $CDCl_3$)** δ −116.24. **HRMS (EI)** (m/z): $[M]^+$ calcd for $C_{15}H_{11}OF$ 226.0788; found 226.0781.

3-(4-(trifluoromethyl)phenyl)-2,3-dihydro-1H-inden-1-one (3j) [4]

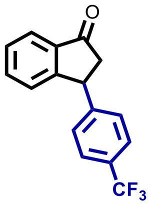

Compound **3j** was synthesized using general procedure B from benzaldehyde (30.6 µL, 0.3 mmol, 3 equiv.) and 1-ethynyl-4-(trifluoromethyl)benzene (16.3 µL, 0.1 mmol, 1 equiv.) The reaction was performed three times, combined and isolated. Purification via column chromatography using petroleum ether/ethyl acetate solvent mixture as an eluent yielded **3j** as a white solid in 66% yield (54.9 mg). **^{1}H NMR (400 MHz, $CDCl_3$)** δ 7.85 – 7.83 (m, 1H), 7.62 – 7.56 (m, 3H), 7.46 (t, *J* = 7.5 Hz, 1H), 7.26 – 7.24 (m, 3H), 4.65 (dd, *J* = 8.2, 3.9 Hz, 1H), 3.26 (dd, *J* = 19.2, 8.1 Hz, 1H), 2.66 (dd, *J* = 19.2, 3.9 Hz, 1H). **^{13}C{^{1}H} NMR (101 MHz, $CDCl_3$)** δ 205.2, 157.0, 147.9 (d, *J* = 1.4 Hz), 136.9, 135.4, 128.4, 128.1, 126.9, 126.0 (q, *J* = 3.7 Hz), 124.1 (d, *J* = 272.0 Hz), 123.8, 46.6, 44.3. **^{19}F NMR (377 MHz, $CDCl_3$)** δ −63.03. **HRMS (EI)** (m/z): $[M]^+$ calcd for $C_{16}H_{11}OF_3$ 276.0756; found 276.0758.

3-(3,5-difluorophenyl)-2,3-dihydro-1H-inden-1-one (3k) [5]

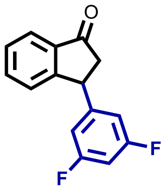

Compound **3k** was synthesized using general procedure B from benzaldehyde (30.6 µL, 0.3 mmol, 3 equiv.) and 1-ethynyl-3,5-difluorobenzene (11.9 µL, 0.1 mmol, 1 equiv.) The reaction was performed three times, combined and isolated. Purification via column chromatography using petroleum ether/ethyl acetate solvent mixture as an eluent yielded **3k** as a white solid in 71% yield (52.1 mg). **^{1}H NMR (400 MHz, $CDCl_3$)** δ 7.82 (d, *J* = 7.7 Hz, 1H), 7.61 (td, *J* = 7.5, 1.3 Hz, 1H), 7.48 – 7.44 (m, 1H), 7.29 (dd, *J* = 7.8, 0.9 Hz, 1H), 6.72 – 6.62 (m, 3H), 4.57 (dd, *J* = 8.2, 3.8 Hz, 1H), 3.23 (dd, *J* = 19.2, 8.2 Hz, 1H), 2.63 (dd, *J* = 19.2, 3.8 Hz, 1H). **^{13}C{^{1}H} NMR (101 MHz, $CDCl_3$)** δ 204.8, 163.5 (d, *J* = 249.5 Hz), 163.3 (d, *J* = 249.5 Hz), 156.3, 147.7 (t, *J* = 8.5 Hz), 136.9, 135.4, 128.5, 126.8, 123.8, 110.7 (d, *J* = 11.7 Hz), 110.7 (d, *J* = 25.5 Hz), 102.7 (t, *J* = 25.3 Hz), 46.4, 44.1 (t, *J* = 2.1 Hz). **^{19}F NMR (377 MHz, $CDCl_3$)** δ −109.45. **HRMS (EI)** (m/z): $[M]^+$ calcd for $C_{15}H_{10}OF_2$ 244.0694; found 244.0695.

3-(3,5-bis(trifluoromethyl)phenyl)-2,3-dihydro-1H-inden-1-one (3l) [5]

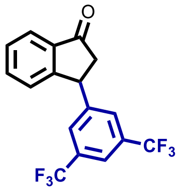

Compound **3l** was synthesized using general procedure B from benzaldehyde (30.6 µL, 0.3 mmol, 3 equiv.) and 1-ethynyl-3,5-bis(trifluoromethyl)benzene (17.7 µL, 0.1 mmol, 1 equiv.) The reaction was performed three times, combined and isolated. Purification via column chromatography using petroleum ether/ethyl acetate solvent mixture as an eluent yielded **3l** as a white solid in 47% yield (48.4 mg). **^{1}H NMR (400 MHz, CDCl$_3$)** δ 7.88 (d, *J* = 7.7 Hz, 1H), 7.80 – 7.79 (m, 1H), 7.64 (td, *J* = 7.5, 1.3 Hz, 1H), 7.58 – 7.57 (m, 2H), 7.53 – 7.49 (m, 1H), 7.26 – 7.23 (m, 1H), 4.74 (dd, *J* = 8.3, 4.0 Hz, 1H), 3.31 (dd, *J* = 19.2, 8.2 Hz, 1H), 2.65 (dd, *J* = 19.2, 4.0 Hz, 1H). **^{13}C{^{1}H} NMR (101 MHz, CDCl$_3$)** δ 204.2, 155.7, 146.4, 137.0, 135.7, 132.5 (q, *J* = 33.5 Hz), 128.9, 128.0, 126.6, 124.1, 123.2 (d, *J* = 272.8 Hz), 121.3 (d, *J* = 3.8 Hz), 46.5, 44.1. **^{19}F NMR (377 MHz, CDCl$_3$)** δ −63.37. **HRMS (EI)** (m/z): [M]$^+$ calcd for $C_{17}H_{10}OF_6$ 344.0630; found 344.0635.

Methyl 4-(3-oxo-2,3-dihydro-1H-inden-1-yl)benzoate (3m) [3]

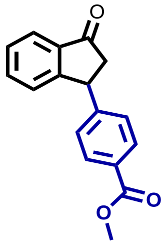

Compound **3m** was synthesized using general procedure B from benzaldehyde (30.6 µL, 0.3 mmol, 3 equiv.) and methyl 4-ethynylbenzoate (16.0 mg, 0.1 mmol, 1 equiv.) The reaction was performed three times, combined and isolated. Purification via column chromatography using petroleum ether/ethyl acetate solvent mixture as an eluent yielded **3m** as a yellow solid in 52% yield (41.7 mg). **^{1}H NMR (400 MHz, CDCl$_3$)** δ 7.98 (d, *J* = 8.4 Hz, 2H), 7.84 – 7.81 (m, 1H), 7.58 (td, *J* = 7.5, 1.3 Hz, 1H), 7.46 – 7.42 (m, 1H), 7.24 (dd, *J* = 7.7, 0.9 Hz, 1H), 7.19 (d, *J* = 8.3 Hz, 2H), 4.64 (dd, *J* = 8.2, 3.8 Hz, 1H), 3.90 (s, 3H), 3.25 (dd, *J* = 19.2, 8.1 Hz, 1H), 2.67 (dd, *J* = 19.2, 3.9 Hz, 1H). **^{13}C{^{1}H} NMR (101 MHz, CDCl$_3$)** δ 205.4, 166.8, 157.1, 149.0, 136.9, 135.3, 130.4, 129.1, 128.3, 127.8, 126.9, 123.7, 52.2, 46.6, 44.4. **HRMS (EI)** (m/z): [M]$^+$ calcd for $C_{17}H_{14}O_3$ 266.0937; found 266.0944.

3-(4-(4,4,5,5-tetramethyl-1,3,2-dioxaborolan-2-yl)phenyl)-2,3-dihydro-1H-inden-1-one (3n) [3]

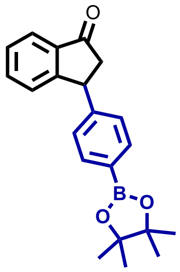

Compound **3n** was synthesized using general procedure B from benzaldehyde (30.6 µL, 0.3 mmol, 3 equiv.) and 2-(4-ethynylphenyl)-4,4,5,5-tetramethyl-1,3,2-dioxaborolane (22.8 mg, 0.1 mmol, 1 equiv.) The reaction was performed three times, combined and isolated. Purification via column chromatography using petroleum ether/ethyl acetate solvent mixture as an eluent yielded **3n** as a white solid in 69% yield (69.4 mg). **^{1}H NMR (400 MHz, $CDCl_3$)** δ 7.81 (d, *J* = 7.7 Hz, 1H), 7.76 (d, *J* = 8.1 Hz, 2H), 7.55 (td, *J* = 7.5, 1.3 Hz, 1H), 7.44 – 7.40 (m, 1H), 7.24 (dd, *J* = 7.7, 1.0 Hz, 1H), 7.14 (d, *J* = 8.1 Hz, 2H), 4.58 (dd, *J* = 8.1, 3.9 Hz, 1H), 3.23 (dd, *J* = 19.2, 8.1 Hz, 1H), 2.69 (dd, *J* = 19.2, 3.9 Hz, 1H), 1.33 (s, 12H). **^{13}C{^{1}H} NMR (101 MHz, $CDCl_3$)** δ 206.0, 157.8, 146.9, 136.8, 135.5, 135.2, 128.0, 127.2, 126.9, 123.5, 83.9, 46.7, 44.7, 25.0, 24.9. **^{11}B NMR (128 MHz, $CDCl_3$)** δ 31.39. **HRMS (EI)** (m/z): $[M]^+$ calcd for $C_{21}H_{23}BO_3$ 334.1734; found 334.1735.

3-(pyridin-2-yl)-2,3-dihydro-1H-inden-1-one (3o) [3]

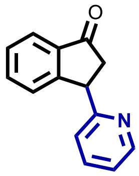

Compound **3o** was synthesized using general procedure B from benzaldehyde (30.6 µL, 0.3 mmol, 3 equiv.) and 2-ethynylpyridine (10.1 µL, 0.1 mmol, 1 equiv.) The reaction was performed three times, combined and isolated. Purification via column chromatography using petroleum ether/ethyl acetate solvent mixture as an eluent yielded **3o** as a white solid in 77% yield (48.5 mg). **^{1}H NMR (400 MHz, $CDCl_3$)** δ 8.55 – 8.53 (m, 1H), 7.80 (d, *J* = 7.7 Hz, 1H), 7.64 (td, *J* = 7.7, 1.8 Hz, 1H), 7.55 (td, *J* = 7.5, 1.3 Hz, 1H), 7.42 – 7.38 (m, 1H), 7.29 (dd, *J* = 7.7, 0.9 Hz, 1H), 7.18 – 7.14 (m, 2H), 4.76 (dd, *J* = 8.0, 3.9 Hz, 1H), 3.19 (dd, *J* = 19.0, 8.0 Hz, 1H), 2.99 (dd, *J* = 18.9, 3.9 Hz, 1H). **^{13}C{^{1}H} NMR (101 MHz, $CDCl_3$)** δ 205.8, 162.2, 156.6, 149.9, 137.0, 136.8, 135.0, 128.1, 126.6, 123.8, 122.2, 122.1, 46.5, 44.6. **HRMS (EI)** (m/z): $[M]^+$ calcd for $C_{14}H_{11}NO$ 209.0835; found 209.0837.

7-methoxy-3-phenyl-2,3-dihydro-1H-inden-1-one (3p)

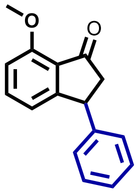

Compound **3p** was synthesized using general procedure B from 2-methoxybenzaldehyde (40.8 mg, 0.3 mmol, 3 equiv.) and phenylacetylene (10.9 μL, 0.1 mmol, 1 equiv.) The reaction was performed three times, combined and isolated. Purification via column chromatography using petroleum ether/ethyl acetate solvent mixture as an eluent yielded **3p** as a yellow oil in 65% yield (46.2 mg). **^{1}H NMR (400 MHz, CDCl$_3$)** δ 7.49 (dd, *J* = 8.2, 7.6 Hz, 1H), 7.32 – 7.21 (m, 3H), 7.14 – 7.11 (m, 2H), 6.83 – 6.77 (m, 2H), 4.49 (dd, *J* = 8.3, 4.0 Hz, 1H), 3.99 (s, 3H), 3.20 (dd, *J* = 18.9, 8.2 Hz, 1H), 2.68 (dd, *J* = 18.9, 4.0 Hz, 1H). **^{13}C{^{1}H} NMR (101 MHz, CDCl$_3$)** δ 203.7, 160.7, 157.8, 143.8, 136.9, 128.9, 127.7, 127.0, 125.0, 118.7, 109.3, 56.0, 47.3, 44.1. **HRMS (EI)** (m/z): $[M]^+$ calcd for $C_{16}H_{14}O_2$ 238.0988; found 238.0992.

5-methoxy-3-phenyl-2,3-dihydro-1H-inden-1-one (3q) [4]

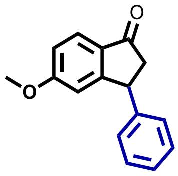

Compound **3q** was synthesized using general procedure B from 4-methoxybenzaldehyde (36.5 μL, 0.3 mmol, 3 equiv.) and phenylacetylene (10.9 μL, 0.1 mmol, 1 equiv.) The reaction was performed three times, combined and isolated. Purification via column chromatography using petroleum ether/ethyl acetate solvent mixture as an eluent yielded **3q** as a yellow solid in 51% yield (36.5 mg). **^{1}H NMR (400 MHz, CDCl$_3$)** δ 7.75 (d, *J* = 8.6 Hz, 1H), 7.34 – 7.23 (m, 3H), 7.14 – 7.12 (m, 2H), 6.94 (ddd, *J* = 8.5, 2.3, 0.7 Hz, 1H), 6.66 (dd, *J* = 2.3, 1.0 Hz, 1H), 4.50 (dd, *J* = 8.1, 3.8 Hz, 1H), 3.79 (s, 3H), 3.21 (dd, *J* = 19.0, 8.1 Hz, 1H), 2.66 (dd, *J* = 19.0, 3.8 Hz, 1H). **^{13}C{^{1}H} NMR (101 MHz, CDCl$_3$)** δ 204.2, 165.7, 161.0, 143.8, 130.3, 129.0, 127.7, 127.1, 125.2, 116.1, 109.9, 55.8, 47.2, 44.6. **HRMS (EI)** (m/z): $[M]^+$ calcd for $C_{16}H_{14}O_2$ 238.0988; found 238.0984.

5-isopropoxy-3-phenyl-2,3-dihydro-1H-inden-1-one (3r)

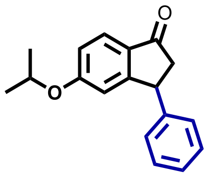

Compound **3r** was synthesized using general procedure B from 4-isopropoxybenzaldehyde (49.3 mg, 0.3 mmol, 3 equiv.) and phenylacetylene (10.9 μL, 0.1 mmol, 1 equiv.) The reaction was performed three times, combined and isolated. Purification via column chromatography using petroleum ether/ethyl acetate solvent mixture as an eluent yielded **3r** as a yellow

solid in 54% yield (42.9 mg). **^{1}H NMR (400 MHz, $CDCl_3$)** δ 7.73 (d, *J* = 8.5 Hz, 1H), 7.34 – 7.29 (m, 2H), 7.27 – 7.22 (m, 1H), 7.14 – 7.12 (m, 2H), 6.90 (dd, *J* = 8.6, 2.2 Hz, 1H), 6.63 (d, *J* = 1.9 Hz, 1H), 4.55 (p, *J* = 6.1 Hz, 1H), 4.49 (dd, *J* = 8.1, 3.8 Hz, 1H), 3.20 (dd, *J* = 19.0, 8.1 Hz, 1H), 2.66 (dd, *J* = 19.0, 3.8 Hz, 1H), 1.30 (dd, *J* = 13.8, 6.1 Hz, 6H). **$^{13}C\{^1H\}$ NMR (101 MHz, $CDCl_3$)** δ 204.2, 164.1, 160.9, 143.8, 129.9, 129.0, 127.7, 127.0, 125.3, 116.9, 111.5, 70.4, 47.2, 44.5, 21.9, 21.8. **HRMS (EI)** (m/z): $[M]^+$ calcd for $C_{18}H_{18}O_2$ 266.1301; found 266.1297.

5-(tert-butyl)-3-phenyl-2,3-dihydro-1H-inden-1-one (3s) [3]

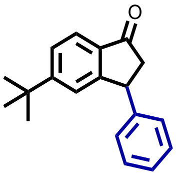

Compound **3s** was synthesized using general procedure B from 4-(tert-butyl)benzaldehyde (50.1 µL, 0.3 mmol, 3 equiv.) and phenylacetylene (10.9 µL, 0.1 mmol, 1 equiv.) The reaction was performed three times, combined and isolated. Purification via column chromatography using petroleum ether/ethyl acetate solvent mixture as an eluent yielded **3s** as a yellow solid in 52% yield (41.3 mg). **^{1}H NMR (400 MHz, $CDCl_3$)** δ 7.75 (d, *J* = 8.2 Hz, 1H), 7.48 (ddd, *J* = 8.2, 1.7, 0.7 Hz, 1H), 7.34 – 7.23 (m, 3H), 7.27 – 7.23 (m, 1H), 7.14 – 7.12 (m, 2H), 4.56 (dd, *J* = 8.1, 3.8 Hz, 1H), 3.22 (dd, *J* = 19.1, 8.1 Hz, 1H), 2.66 (dd, *J* = 19.1, 3.8 Hz, 1H), 1.29 (s, 9H). **$^{13}C\{^1H\}$ NMR (101 MHz, $CDCl_3$)** δ 205.7, 159.5, 158.2, 144.0, 134.7, 129.0, 127.8, 127.0, 125.8, 123.4, 123.1, 47.4, 44.7, 35.7, 31.3. **HRMS (EI)** (m/z): $[M]^+$ calcd for $C_{19}H_{20}O$ 264.1508; found 264.1502.

5,7-dimethyl-3-phenyl-2,3-dihydro-1H-inden-1-one (3t) [3]

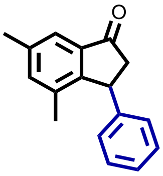

Compound **3t** was synthesized using general procedure B from 3,5-dimethylbenzaldehyde (40.3 µL, 0.3 mmol, 3 equiv.) and phenylacetylene (10.9 µL, 0.1 mmol, 1 equiv.) The reaction was performed three times, combined and isolated. Purification via column chromatography using petroleum ether/ethyl acetate solvent mixture as an eluent yielded **3t** as a yellow oil in 70% yield (49.5 mg). **^{1}H NMR (400 MHz, $CDCl_3$)** δ 7.48 (s, 1H), 7.28 – 7.18 (m, 4H), 7.03 – 7.01 (m, 2H), 4.53 (dd, *J* = 8.2, 2.6 Hz, 1H), 3.23 (dd, *J* = 19.2, 8.2 Hz, 1H), 2.59 (dd, *J* = 19.3, 2.6 Hz, 1H), 2.40 (s, 3H), 1.97 (s, 3H). **$^{13}C\{^1H\}$ NMR (101 MHz, $CDCl_3$)** δ 206.8, 153.2, 144.0, 138.5, 137.8, 137.5, 136.5, 128.9, 127.4, 126.7, 121.0, 48.0, 43.6, 21.1, 18.4. **HRMS (EI)** (m/z): $[M]^+$ calcd for $C_{17}H_{16}O$ 236.1195; found 236.1200.

5-methoxy-7-methyl-3-phenyl-2,3-dihydro-1H-inden-1-one (3u)

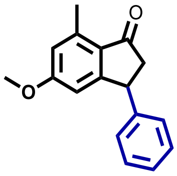

Compound **3u** was synthesized using general procedure B from 4-methoxy-2-methylbenzaldehyde (40.6 µL, 0.3 mmol, 3 equiv.) and phenylacetylene (10.9 µL, 0.1 mmol, 1 equiv.) The reaction was performed three times, combined and isolated. Purification via column chromatography using petroleum ether/ethyl acetate solvent mixture as an eluent yielded **3u** as a yellow solid in 49% yield (37.4 mg). **^{1}H NMR (400 MHz, $CDCl_3$)** δ 7.33 – 7.29 (m, 2H), 7.25 – 7.22 (m, 1H), 7.14 – 7.12 (m, 2H), 6.67 – 6.67 (m, 1H), 6.47 (d, *J* = 2.2 Hz, 1H), 4.43 (dd, *J* = 8.3, 3.9 Hz, 1H), 3.76 (s, 3H), 3.17 (dd, *J* = 18.8, 8.3 Hz, 1H), 2.67 – 2.62 (m, 4H). **^{13}C{^{1}H} NMR (101 MHz, $CDCl_3$)** δ 205.1, 164.8, 161.9, 144.1, 140.6, 128.9, 127.8, 126.9, 117.1, 107.7, 55.6, 47.7, 44.2, 18.7.**HRMS (EI)** (m/z): $[M]^+$ calcd for $C_{17}H_{16}O_2$ 252.1144; found 252.1149.

7-chloro-3-phenyl-2,3-dihydro-1H-inden-1-one (3v) [3]

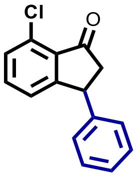

Compound **3v** was synthesized using general procedure B from 2-chlorobenzaldehyde (33.8 µL, 0.3 mmol, 3 equiv.) and phenylacetylene (10.9 µL, 0.1 mmol, 1 equiv.) The reaction was performed three times, combined and isolated. Purification via column chromatography using petroleum ether/ethyl acetate solvent mixture as an eluent yielded **3v** as a yellow oil in 51% yield (36.8 mg). **^{1}H NMR (400 MHz, $CDCl_3$)** δ 7.35 (t, *J* = 7.7 Hz, 1H), 7.25 – 7.21 (m, 3H), 7.18 – 7.14 (m, 1H), 7.06 – 7.01 (m, 3H), 4.42 (dd, *J* = 8.3, 4.1 Hz, 1H), 3.16 (dd, *J* = 19.2, 8.2 Hz, 1H), 2.65 (dd, *J* = 19.2, 4.1 Hz, 1H). **^{13}C{^{1}H} NMR (101 MHz, $CDCl_3$)** δ 202.7, 160.4, 143.2, 135.4, 132.6, 131.6, 129.6, 129.1, 127.7, 127.3, 125.5, 47.5, 43.7. **HRMS (EI)** (m/z): $[M]^+$ calcd for $C_{15}H_{11}OCl$ 242.0492; found 242.0492.

5-chloro-3-phenyl-2,3-dihydro-1H-inden-1-one (3w) [4]

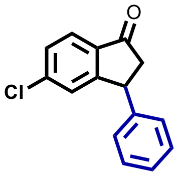

Compound **3w** was synthesized using general procedure B from 4-chlorobenzaldehyde (42.2 mg, 0.3 mmol, 3 equiv.) and phenylacetylene (10.9 µL, 0.1 mmol, 1 equiv.) The reaction was performed three times, combined and isolated. Purification via column chromatography using petroleum ether/ethyl acetate solvent mixture as an eluent yielded **3w** as a yellow solid in 59% yield (43.0 mg). **^{1}H NMR (400 MHz, $CDCl_3$)** δ 7.74 (d, *J* = 8.2 Hz, 1H), 7.39 (ddd, *J* = 8.2, 1.8, 0.8 Hz, 1H),

7.36 – 7.31 (m, 2H), 7.30 – 7.25 (m, 2H), 7.13 – 7.11 (m, 2H), 4.54 (dd, *J* = 8.1, 4.0 Hz, 1H), 3.24 (dd, *J* = 19.3, 8.1 Hz, 1H), 2.72 (dd, *J* = 19.3, 3.9 Hz, 1H). **^{13}C{^{1}H} NMR (101 MHz, CDCl$_3$)** δ 204.5, 159.5, 142.9, 141.7, 135.3, 129.2, 128.9, 127.7, 127.4, 127.1, 124.7, 46.9, 44.3, 29.8. **HRMS (EI)** (m/z): $[M]^+$ calcd for $C_{15}H_{11}OCl$ 242.0492; found 242.0491.

5-fluoro-3-phenyl-2,3-dihydro-1H-inden-1-one (3x)

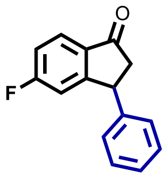

Compound **3x** was synthesized using general procedure B from 4-fluorobenzaldehyde (32.2 µL, 0.3 mmol, 3 equiv.) and phenylacetylene (10.9 µL, 0.1 mmol, 1 equiv.) The reaction was performed three times, combined and isolated. Purification via column chromatography using petroleum ether/ethyl acetate solvent mixture as an eluent yielded **3x** as a yellow solid in 49% yield (33.3 mg). **^{1}H NMR (400 MHz, CDCl$_3$)** δ 7.82 (dd, *J* = 8.5, 5.3 Hz, 1H), 7.35 – 7.31 (m, 2H), 7.29 – 7.27 (m, 1H), 7.13 – 7.11 (m, 3H), 6.91 (ddd, *J* = 8.5, 2.7, 0.9 Hz, 1H), 4.54 (dd, *J* = 8.2, 3.9 Hz, 1H), 3.25 (dd, *J* = 19.3, 8.1 Hz, 1H), 2.73 (dd, *J* = 19.2, 3.9 Hz, 1H). **^{13}C{^{1}H} NMR (101 MHz, CDCl$_3$)** δ 204.1, 167.5 (d, *J* = 256.9 Hz), 161.0, 143.0, 133.3, 129.2, 127.7, 127.4, 125.9 (d, *J* = 10.4 Hz), 116.4 (d, *J* = 23.9 Hz), 113.5 (d, *J* = 22.5 Hz), 47.0, 44.4 (d, *J* = 2.1 Hz), 31.0, 29.8.**^{19}F NMR (377 MHz, CDCl$_3$)** δ −102.43. **HRMS (EI)** (m/z): $[M]^+$ calcd for $C_{15}H_{11}OF$ 226.0788; found 226.0789.

5-(tert-butyl)-3-(4-(tert-butyl)phenyl)-2,3-dihydro-1H-inden-1-one (3y)

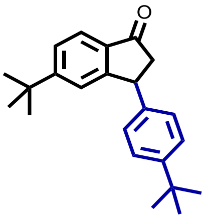

Compound **3y** was synthesized using general procedure B from 4-(tert-butyl)benzaldehyde (50.1 µL, 0.3 mmol, 3 equiv.) and 1-(tert-butyl)-4-ethynylbenzene (18.0 µL, 0.1 mmol, 1 equiv.). The reaction was performed three times, combined and isolated. Purification via column chromatography using petroleum ether/ethyl acetate solvent mixture as an eluent yielded **3y** as a yellow solid in 63% yield (60.6 mg). **^{1}H NMR (400 MHz, CDCl$_3$)** δ 7.74 (d, *J* = 8.1Hz, 1H), 7.47 (ddd, *J* = 8.2, 1.7, 0.7 Hz, 1H), 7.33 (d, *J* = 8.4 Hz, 2H), 7.30 – 7.29 (m, 1H), 7.05 (d, *J* = 8.3 Hz, 2H), 4.54 (dd, *J* = 8.1, 3.8 Hz, 1H), 3.19 (dd, *J* = 19.1, 8.0 Hz, 1H), 2.64 (dd, *J* = 19.1, 3.8 Hz, 1H), 1.32 (s, 9H), 1.30 (s, 9H). **^{13}C{^{1}H} NMR (101 MHz, CDCl$_3$)** δ 205.9, 159.4, 158.2, 149.8, 140.8, 134.7, 127.3, 125.8, 125.7, 123.5, 123.1, 47.5, 44.1, 35.7, 34.6, 31.4, 31.3. **HRMS (EI)** (m/z): $[M]^+$ calcd for $C_{23}H_{28}O$ 320.2134; found 320.2128.

3-(4-(tert-butyl)phenyl)-4,6-dimethyl-2,3-dihydro-1H-inden-1-one (3z)

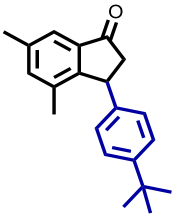

Compound **3z** was synthesized using general procedure B from 3,5-dimethylbenzaldehyde (40.3 µL, 0.3 mmol, 3 equiv.) and 1-(tert-butyl)-4-ethynylbenzene (18.0 µL, 0.1 mmol, 1 equiv.). The reaction was performed three times, combined and isolated. Purification via column chromatography using petroleum ether/ethyl acetate solvent mixture as an eluent yielded **3z** as a yellow oil in 67% yield (59.1 mg). **^{1}H NMR (400 MHz, $CDCl_3$)** δ 7.47 (s, 1H), 7.28 – 7.24 (m, 2H), 7.20 (s, 1H), 6.94 (d, *J* = 8.3 Hz, 2H), 4.50 (dd, *J* = 8.3, 2.5 Hz, 1H), 3.20 (dd, *J* = 19.2, 8.2 Hz, 1H), 2.60 (dd, *J* = 19.2, 2.5 Hz, 1H), 2.39 (s, 3H), 1.99 (s, 3H), 1.28 (s, 9H). **^{13}C{^{1}H} NMR (101 MHz, $CDCl_3$)** δ 207.1, 153.5, 149.5, 140.8, 138.3, 137.7, 137.4, 136.5, 127.0, 125.7, 121.0, 48.1, 43.1, 34.531.4, 21.1, 18.4. **HRMS (EI)** (m/z): $[M]^+$ calcd for $C_{21}H_{24}O$ 292.1821; found 292.1817.

5-isopropoxy-3-(3-methoxyphenyl)-2,3-dihydro-1H-inden-1-one (3aa)

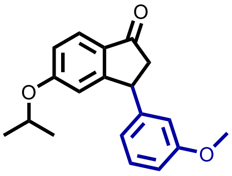

Compound **3aa** was synthesized using general procedure B from 4-isopropoxybenzaldehyde (49.3 mg, 0.3 mmol, 3 equiv.) and 1-ethynyl-3-methoxybenzene (12.7 µL, 0.1 mmol, 1 equiv.). The reaction was performed three times, combined and isolated. Purification via column chromatography using petroleum ether/ethyl acetate solvent mixture as an eluent yielded **3aa** as a colourless oil in 50% yield (44.7 mg). **^{1}H NMR (400 MHz, $CDCl_3$)** δ 7.72 (d, *J* = 8.6 Hz, 1H), 7.25 – 7.21 (m, 1H), 6.90 (ddd, *J* = 8.5, 2.3, 0.6 Hz, 1H), 6.78 (ddd, *J* = 8.2, 2.6, 0.9 Hz, 1H), 6.72 (d, *J* = 7.7 Hz, 1H), 6.66 (ddd, *J* = 5.4, 2.6, 1.2 Hz, 2H), 4.56 (p, *J* = 6.1 Hz, 1H), 4.46 (dd, *J* = 8.1, 3.8 Hz, 1H), 3.77 (s, 3H), 3.18 (dd, *J* = 19.0, 8.1 Hz, 1H), 2.66 (dd, *J* = 19.0, 3.8 Hz, 1H), 1.30 (dd, *J* = 11.4, 6.0 Hz, 6H). **^{13}C{^{1}H} NMR (101 MHz, $CDCl_3$)** δ 204.1, 164.1, 160.7, 160.1, 145.4, 130.0, 129.8, 125.3, 120.1, 117.0, 113.7, 112.1, 111.5, 70.4, 55.3, 47.0, 44.5, 21.9, 21.8. **HRMS (EI)** (m/z): $[M]^+$ calcd for $C_{19}H_{20}O_3$ 296.1407; found 296.1411.

3-(4-(tert-butyl)phenyl)-5-fluoro-2,3-dihydro-1H-inden-1-one (3ab)

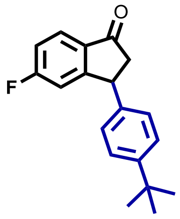

Compound **3ab** was synthesized using general procedure B from 4-fluorobenzaldehyde (32.2 µL, 0.3 mmol, 3 equiv.) and 1-(tert-butyl)-4-ethynylbenzene (18.0 µL, 0.1 mmol, 1 equiv.). The reaction was performed three times, combined and isolated. Purification via column chromatography using petroleum ether/ethyl acetate solvent mixture as an eluent yielded **3ab** as a white solid in 57% yield (48.0 mg). **^{1}H NMR (400 MHz, $CDCl_3$)** δ 7.81 (dd, *J* = 8.5, 5.3 Hz, 1H), 7.34 (d, *J* = 8.4 Hz, 2H), 7.10 (tdd, *J* = 8.6, 2.3, 0.8 Hz, 1H), 7.05 (d, *J* = 8.3 Hz, 2H), 6.93 (ddd, *J* = 8.4, 2.4, 0.9 Hz, 1H), 4.52 (dd, *J* = 8.1, 3.9 Hz, 1H), 3.23 (dd, *J* = 19.2, 8.1 Hz, 1H), 2.73 (dd, *J* = 19.2, 3.9 Hz, 1H), 1.31 (s, 9H). **^{13}C{^{1}H} NMR (101 MHz, $CDCl_3$)** δ 204.3, 161.2, 150.3, 145.5, 139.8, 127.3, 126.0, 125.8 (d, *J* = 10.4 Hz), 116.3 (d, *J* = 23.8 Hz), 113.6 (d, *J* = 22.3 Hz), 47.0, 44.00, 34.6, 31.4. **^{19}F NMR (377 MHz, $CDCl_3$)** δ −102.61. **HRMS (EI)** (m/z): $[M]^+$ calcd for $C_{19}H_{19}FO$ 282.1414; found 282.1417.

3-(3,5-difluorophenyl)-4,6-dimethyl-2,3-dihydro-1H-inden-1-one (3ac)

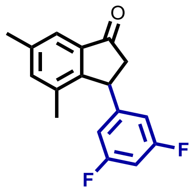

Compound **3ac** was synthesized using general procedure B from 3,5-dimethylbenzaldehyde (40.3 µL, 0.3 mmol, 3 equiv.) and 1-ethynyl-3,5-difluorobenzene (11.9 µL, 0.1 mmol, 1 equiv.) The reaction was performed three times, combined and isolated. Purification via column chromatography using petroleum ether/ethyl acetate solvent mixture as an eluent yielded **3ac** as a colorless oil in 75% yield (60.9 mg). **^{1}H NMR (400 MHz, $CDCl_3$)** δ 7.49 – 7.48 (m, 1H), 7.26 – 7.25 (m, 1H), 6.66 (tt, *J* = 8.9, 2.3 Hz, 1H), 6.57 – 6.51 (m, 2H), 4.52 (dd, *J* = 8.3, 2.6 Hz, 1H), 3.21 (dd, *J* = 19.2, 8.3 Hz, 1H), 2.53 (dd, *J* = 19.2, 2.6 Hz, 1H), 2.41 (s, 3H), 2.01 (s, 3H). **^{13}C{^{1}H} NMR (101 MHz, $CDCl_3$)** δ 205.5, 163.5 (d, *J* = 249.3 Hz), 163.4 (d, *J* = 249.4 Hz), 151.6, 148.23 (t, *J* = 8.5 Hz), 139.2, 138.1, 137.5, 136.3, 121.3, 110.3 (d, *J* = 25.2 Hz), 102.4 (t, *J* = 25.4 Hz), 47.4, 43.26 (t, *J* = 2.0 Hz), 21.2, 18.3. **^{19}F NMR (377 MHz, $CDCl_3$)** δ −109.57. **HRMS (EI)** (m/z): $[M]^+$ calcd for $C_{17}H_{14}OF_2$ 272.1007; found 272.1013.

5-(tert-butyl)-3-(4-fluorophenyl)-2,3-dihydro-1H-inden-1-one (3ad)

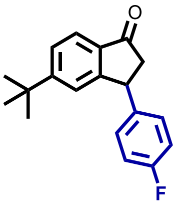

Compound **3ad** was synthesized using general procedure B from 4-(tert-butyl)benzaldehyde (50.1 µL, 0.3 mmol, 3 equiv.) and 1-ethynyl-4-fluorobenzene (12.0 mg, 0.1 mmol, 1 equiv.). The reaction was performed three times, combined and isolated. Purification via column chromatography using petroleum ether/ethyl acetate solvent mixture as an eluent yielded **3ad** as a yellow oil in 82% yield (69.3 mg). **^{1}H NMR (400 MHz, $CDCl_3$)** δ 7.74 (d, *J* = 8.2 Hz, 1H), 7.48 (ddd, *J* = 8.3, 1.7, 0.7 Hz, 1H), 7.23 – 7.22 (m, 1H), 7.11 – 7.06 (m, 2H), 7.02 – 6.98 (m, 2H), 4.54 (dd, *J* = 8.1, 3.8 Hz, 1H), 3.21 (dd, *J* = 19.1, 8.0 Hz, 1H), 2.60 (dd, *J* = 19.1, 3.8 Hz, 1H), 1.29 (s, 9H). **^{13}C{^{1}H} NMR (101 MHz, $CDCl_3$)** δ 205.3, 161.8 (d, *J* = 245.2 Hz), 159.7, 157.9, 139.8 (d, *J* = 3.2 Hz), 134.6, 129.2 (d, *J* = 7.9 Hz), 125.9, 123.2 (d, *J* = 8.8 Hz), 115.8 (d, *J* = 21.5 Hz), 47.4, 43.9, 35.7, 31.3. **^{19}F NMR (377 MHz, $CDCl_3$)** δ −116.45. **HRMS (EI)** (m/z): $[M]^+$ calcd for $C_{19}H_{19}OF$ 282.1414; found 282.1412.

7-methoxy-3-(4-(4,4,5,5-tetramethyl-1,3,2-dioxaborolan-2-yl)phenyl)-2,3-dihydro-1H-inden-1-one (3ae)

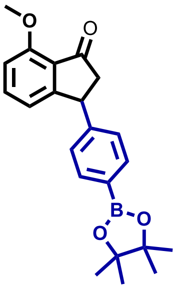

Compound **3ae** was synthesized using general procedure B from 2-methoxybenzaldehyde (40.8 mg, 0.3 mmol, 3 equiv.) and 2-(4-ethynylphenyl)-4,4,5,5-tetramethyl-1,3,2-dioxaborolane (22.8 mg, 0.1 mmol, 1 equiv.). The reaction was performed three times, combined and isolated. Purification via column chromatography using petroleum ether/ethyl acetate solvent mixture as an eluent yielded **3ae** as a yellow oil in 68% yield (74.9 mg). **^{1}H NMR (400 MHz, $CDCl_3$)** δ 7.75 (d, *J* = 8.0 Hz, 2H), 7.49 – 7.45 (m, 1H), 7.14 (d, *J* = 8.1 Hz, 2H), 6.79 (dd, *J* = 26.0, 7.9 Hz, 2H), 4.49 (dd, *J* = 8.2, 3.9 Hz, 1H), 3.99 (s, 3H), 3.19 (dd, *J* = 19.0, 8.2 Hz, 1H), 2.68 (dd, *J* = 19.0, 4.0 Hz, 1H), 1.33 (s, 12H). **^{13}C{^{1}H} NMR (101 MHz, $CDCl_3$)** δ 203.7, 160.5, 157.9, 147.0, 136.9, 135.5, 127.2, 118.7, 109.4, 83.9, 56.0, 47.2, 44.3, 25.0, 24.9. **^{11}B-NMR (128 MHz, $CDCl_3$)** δ 32.22. **HRMS (EI)** (m/z): $[M]^+$ calcd for $C_{22}H_{25}BO_4$ 364.1840; found 364.1833.

References

1. Oswood, C. J.; MacMillan, D. W. Selective Isomerization via Transient Thermodynamic Control: Dynamic Epimerization of Trans to Cis Diols. *J. Am. Chem. Soc.* **2021**, *144* (1), 93–98.
2. Babawale, F.; Murugesan, K.; Narobe, R.; König, B. Synthesis of Unnatural α-Amino Acid Derivatives via Photoredox Activation of Inert $C(sp^3)$–H Bonds. *Org. Lett.* **2022**, *24*, 4793–4797.
3. Yang, B.; Li, S. J.; Wang, Y.; Lan, Y.; Zhu, S. Hydrogen Radical-Shuttle (HRS)-Enabled Photoredox Synthesis of Indanones via Decarboxylative Annulation. *Nat. Commun*. **2021**, 12, 5257
4. Yan, J.; Nie, Y.; Gao, F.; Yuan, Q.; Xie, F.; Zhang, W. Ir-Catalyzed Asymmetric Hydrogenation of 3-Arylindenones for the Synthesis of Chiral 3-Arylindanones. *Tetrahedron* **2021**, *84*, 132003.
5. Tiwari, P. K.; Aidhen, I. S. A Weinreb Amide Based Building Block for Convenient Access to β,β-Diarylacroleins: Synthesis of 3-Arylindanones. *Eur. J. Org. Chem.* **2016**, *2016* (15), 2637–2646.

NMR Spectra

3-phenyl-2,3-dihydro-1H-inden-1-one (3a): ^{1}HNMR (400 MHz), $CDCl_3$.

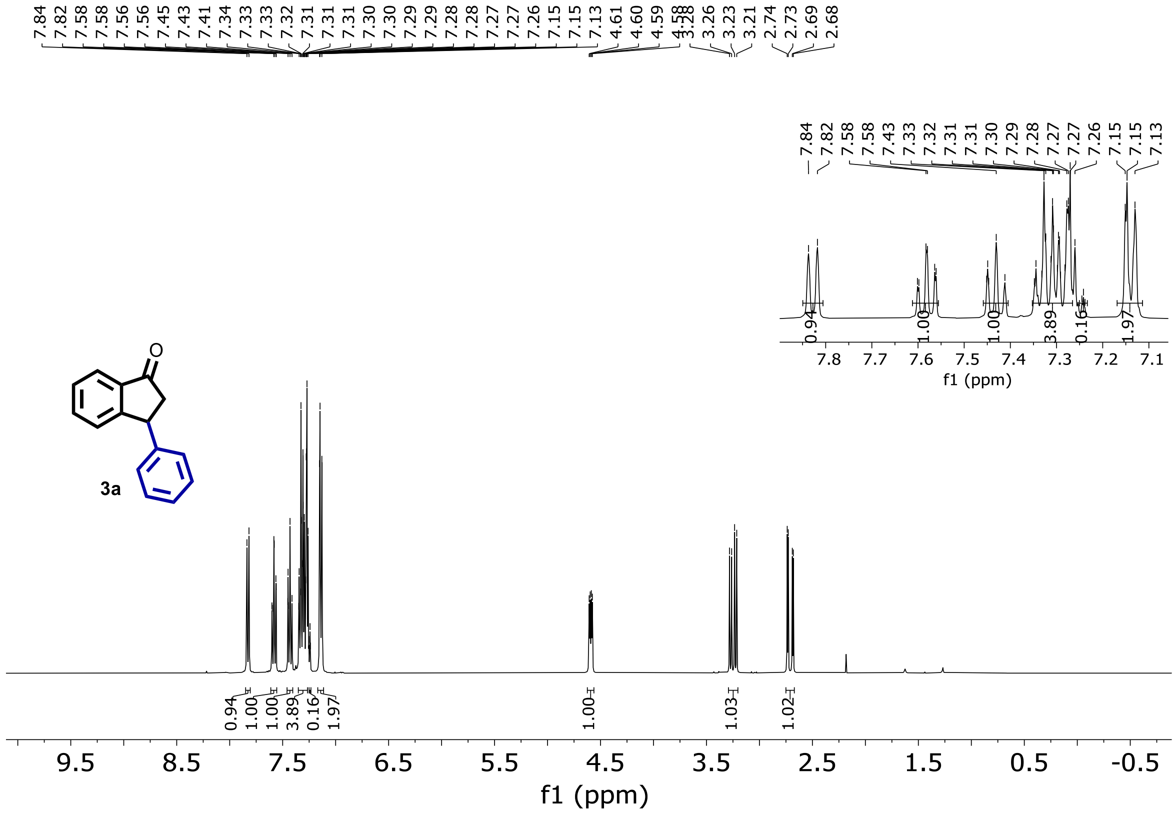

3-phenyl-2,3-dihydro-1H-inden-1-one (3a): $^{13}C\{^{1}H\}$ NMR (101 MHz), $CDCl_3$.

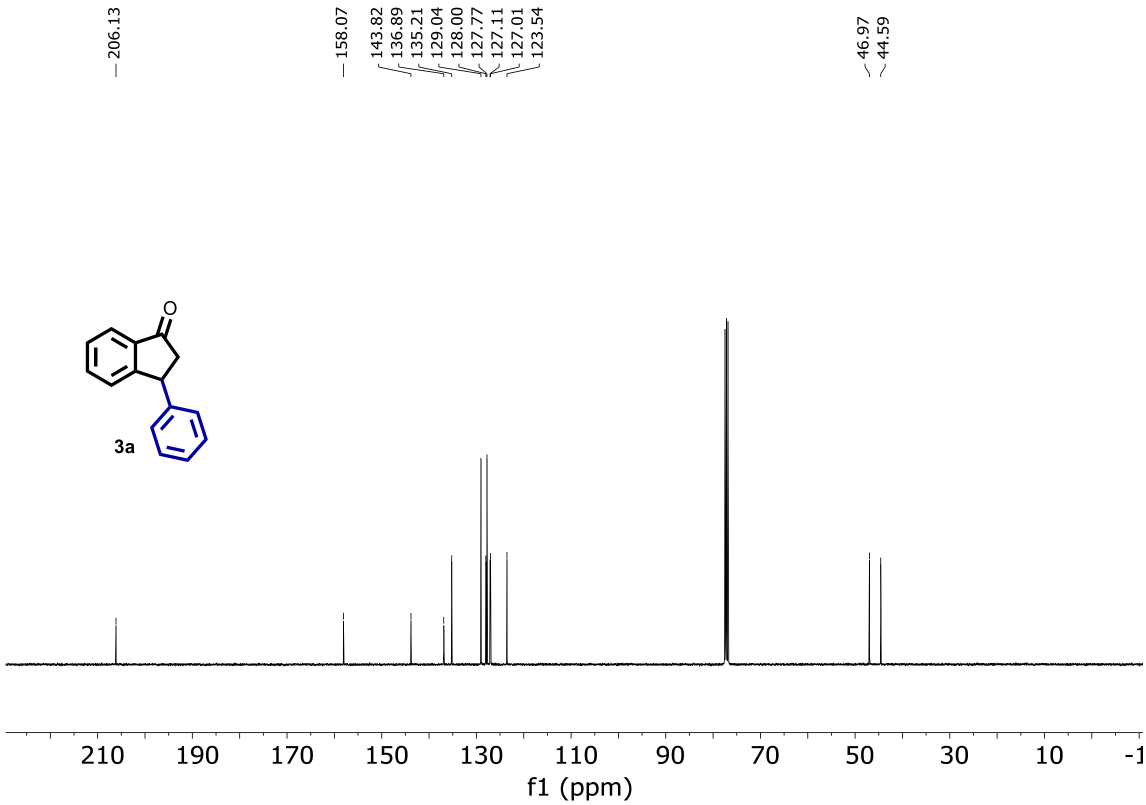

3-(2-methoxyphenyl)-2,3-dihydro-1H-inden-1-one (3b): ^{1}H NMR (400 MHz), CDCl$_3$.

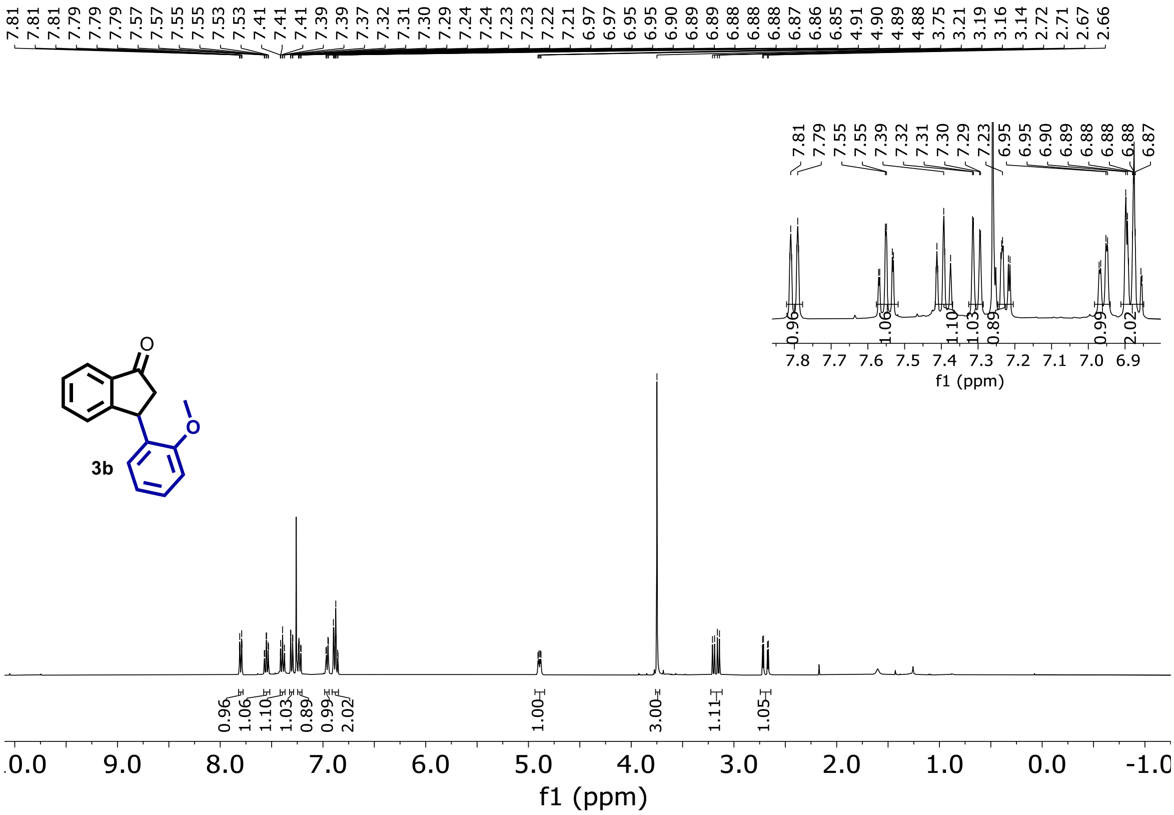

3-(2-methoxyphenyl)-2,3-dihydro-1H-inden-1-one (3b): ^{13}C{^{1}H} NMR (101 MHz), CDCl$_3$.

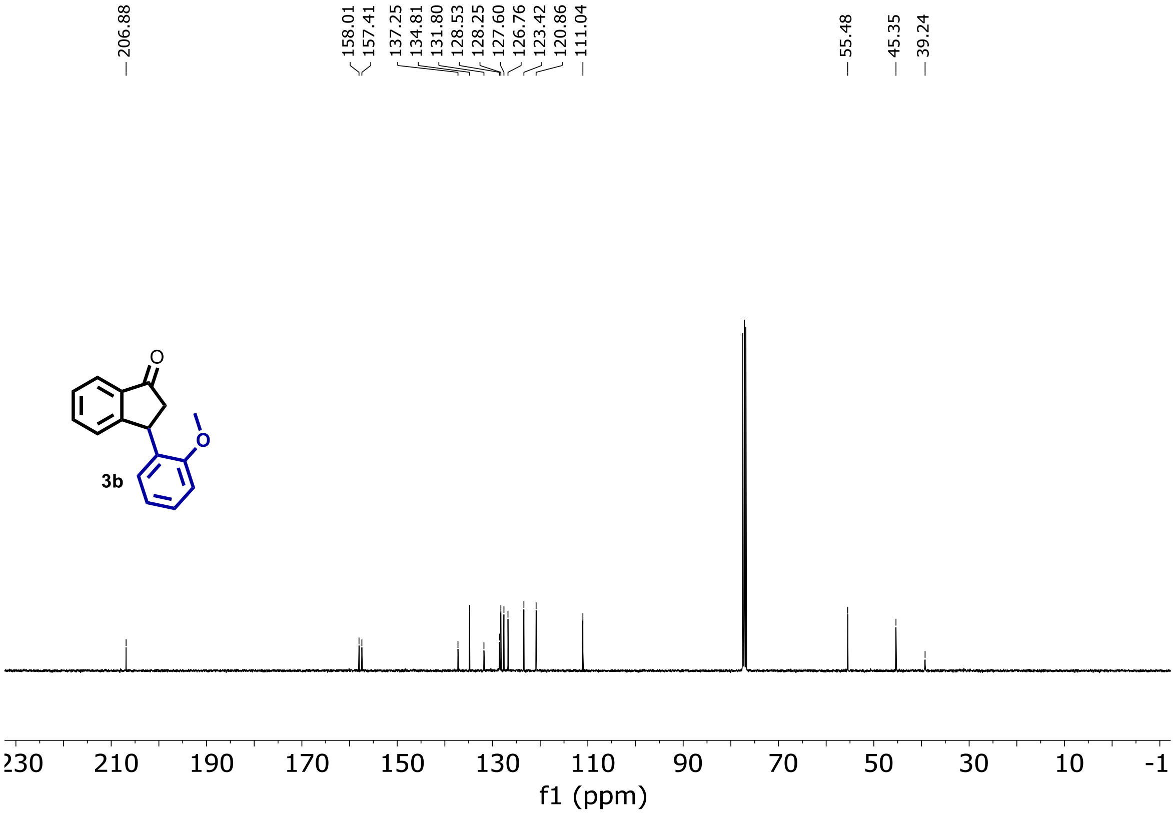

3-(3-methoxyphenyl)-2,3-dihydro-1H-inden-1-one (3c): ^{1}H NMR (400 MHz), $CDCl_3$.

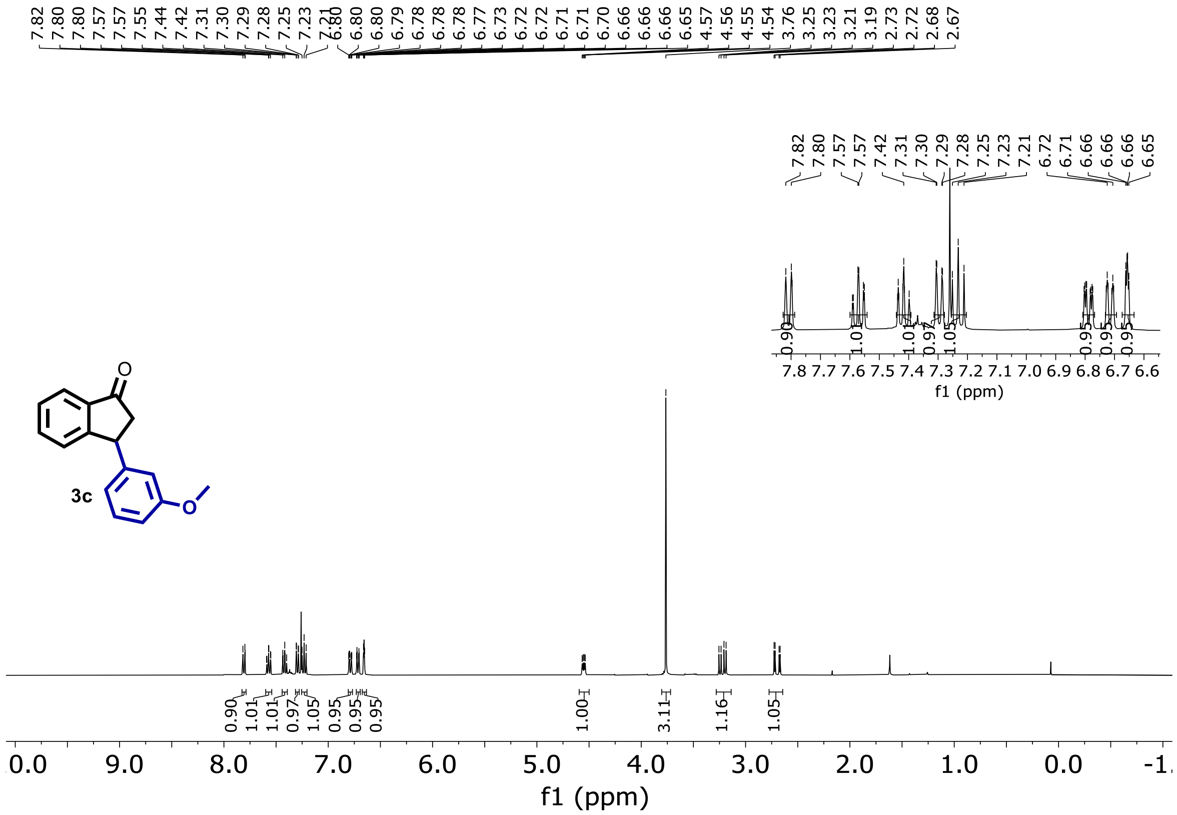

3-(3-methoxyphenyl)-2,3-dihydro-1H-inden-1-one (3c): $^{13}C\{^{1}H\}$ NMR (101 MHz), $CDCl_3$.

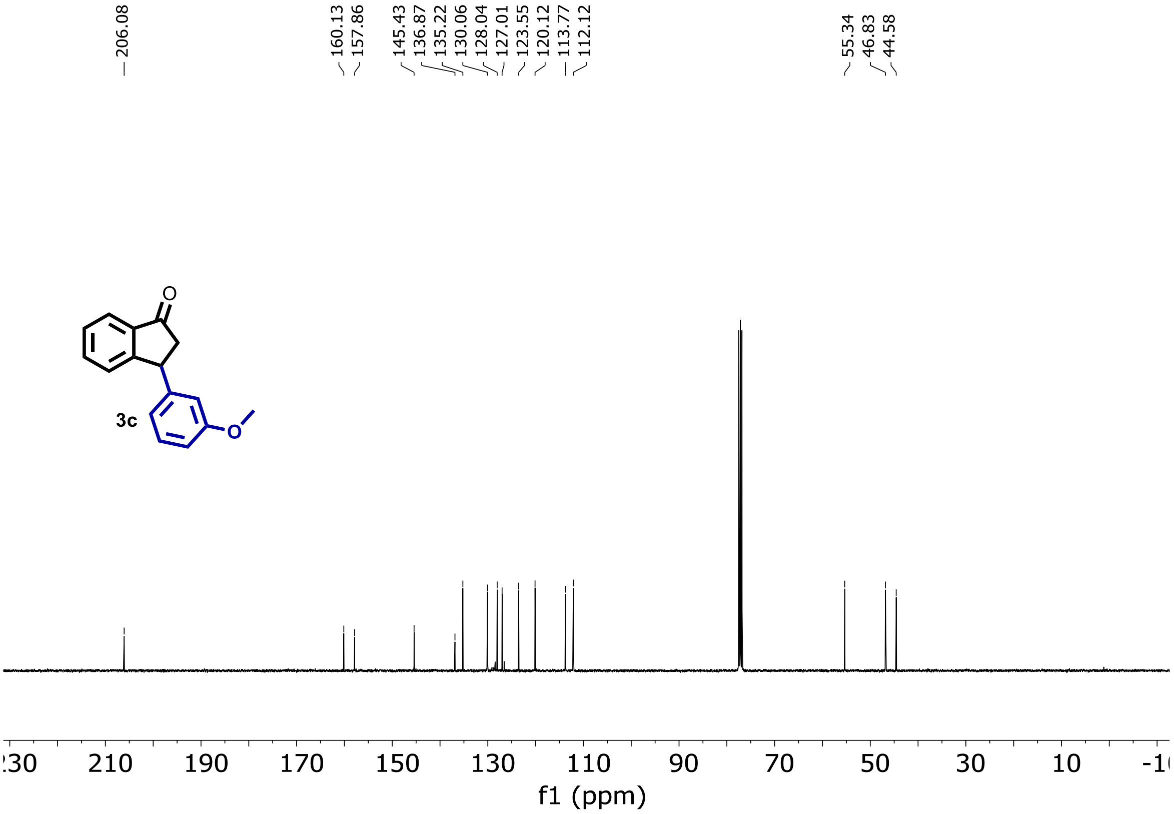

3-(4-methoxyphenyl)-2,3-dihydro-1H-inden-1-one (3d): ^{1}H NMR (400 MHz), CDCl$_3$.

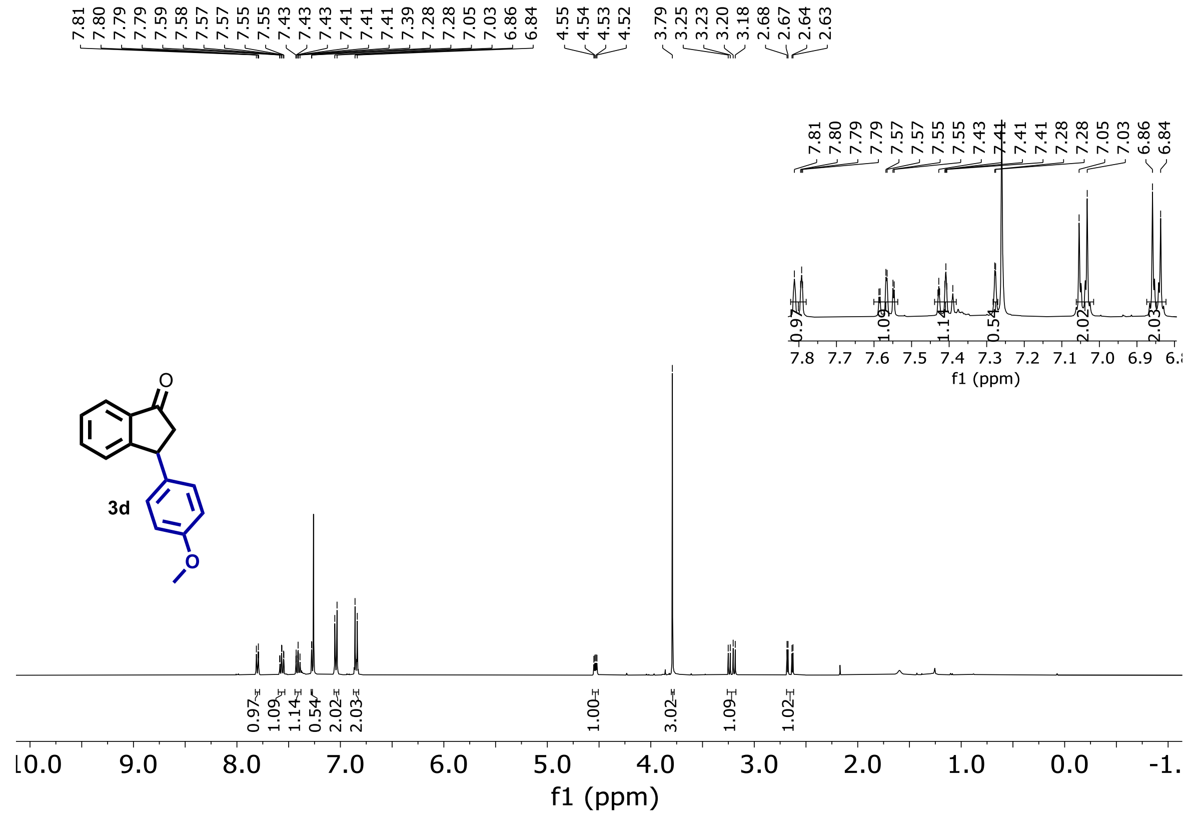

3-(4-methoxyphenyl)-2,3-dihydro-1H-inden-1-one (3d): ^{13}C{^{1}H} NMR (101 MHz), CDCl$_3$.

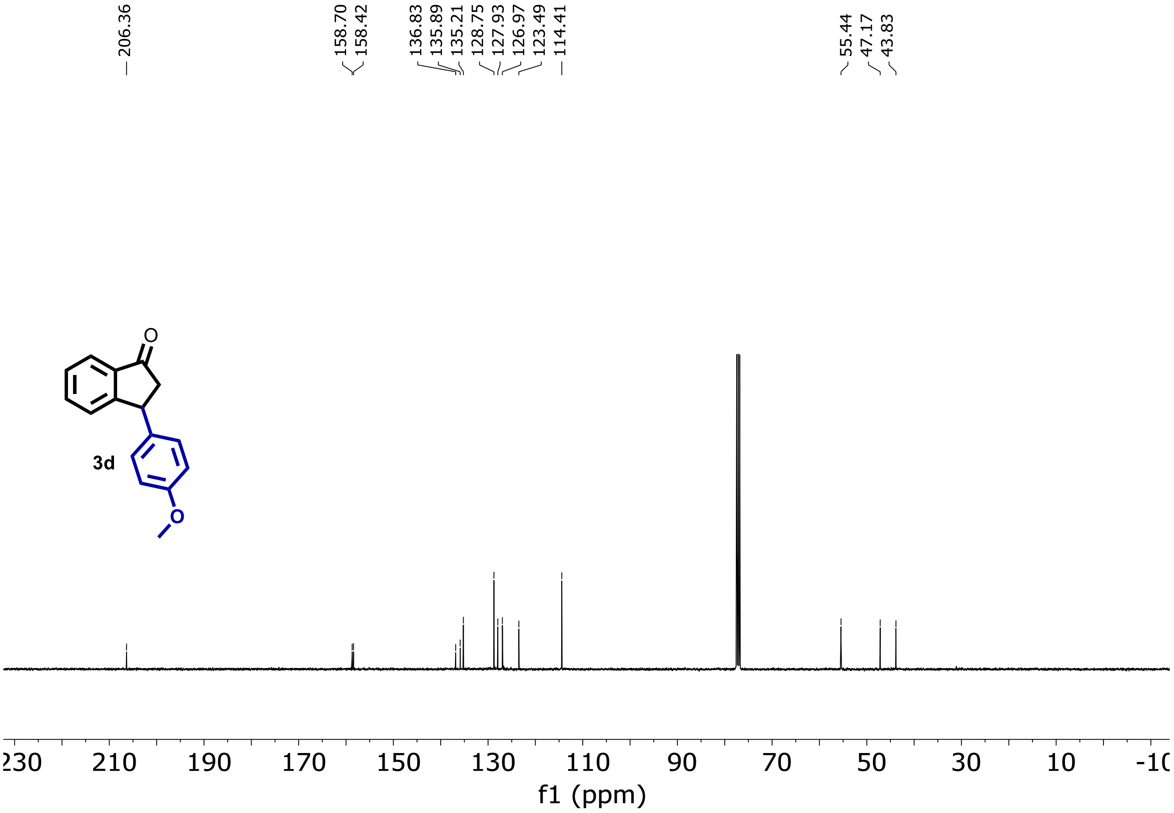

3-(4-(tert-butyl)phenyl)-2,3-dihydro-1H-inden-1-one (3e): ^{1}H NMR (400 MHz), $CDCl_3$.

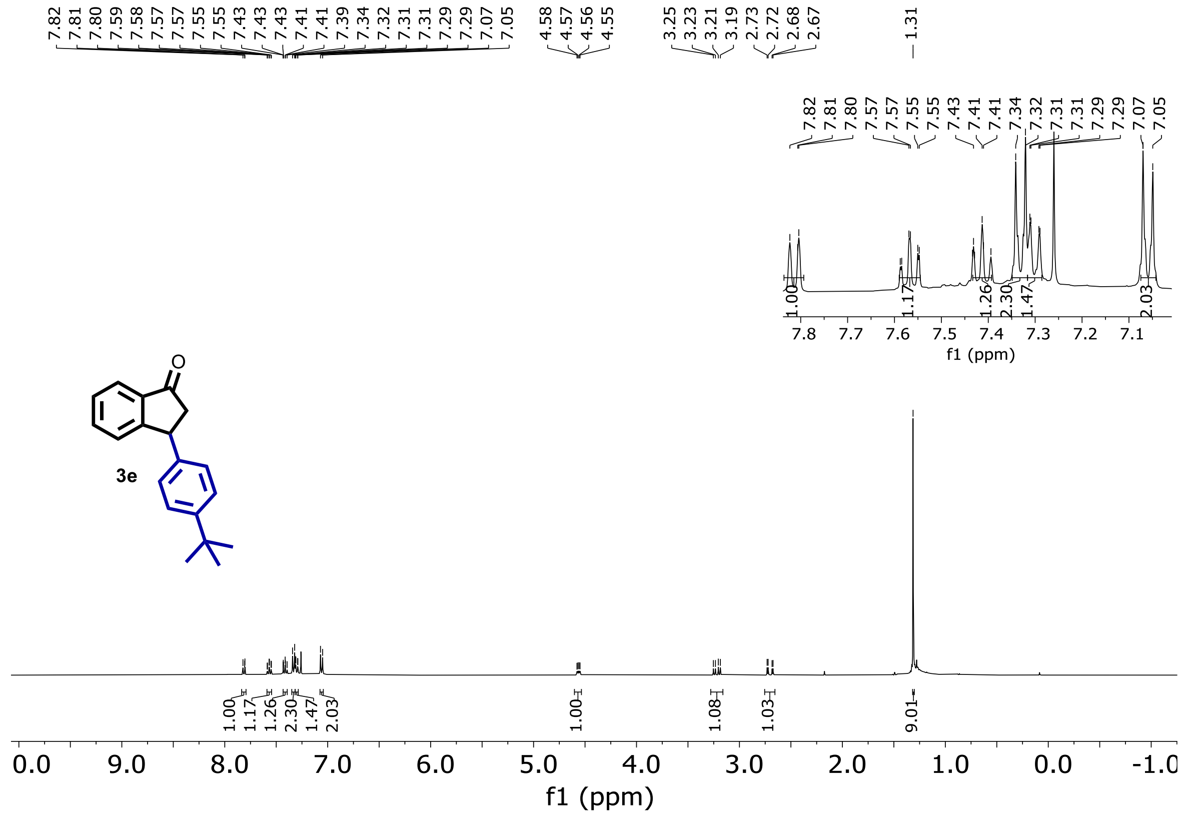

3-(4-(tert-butyl)phenyl)-2,3-dihydro-1H-inden-1-one (3e): $^{13}C\{^{1}H\}$ NMR (101 MHz), $CDCl_3$.

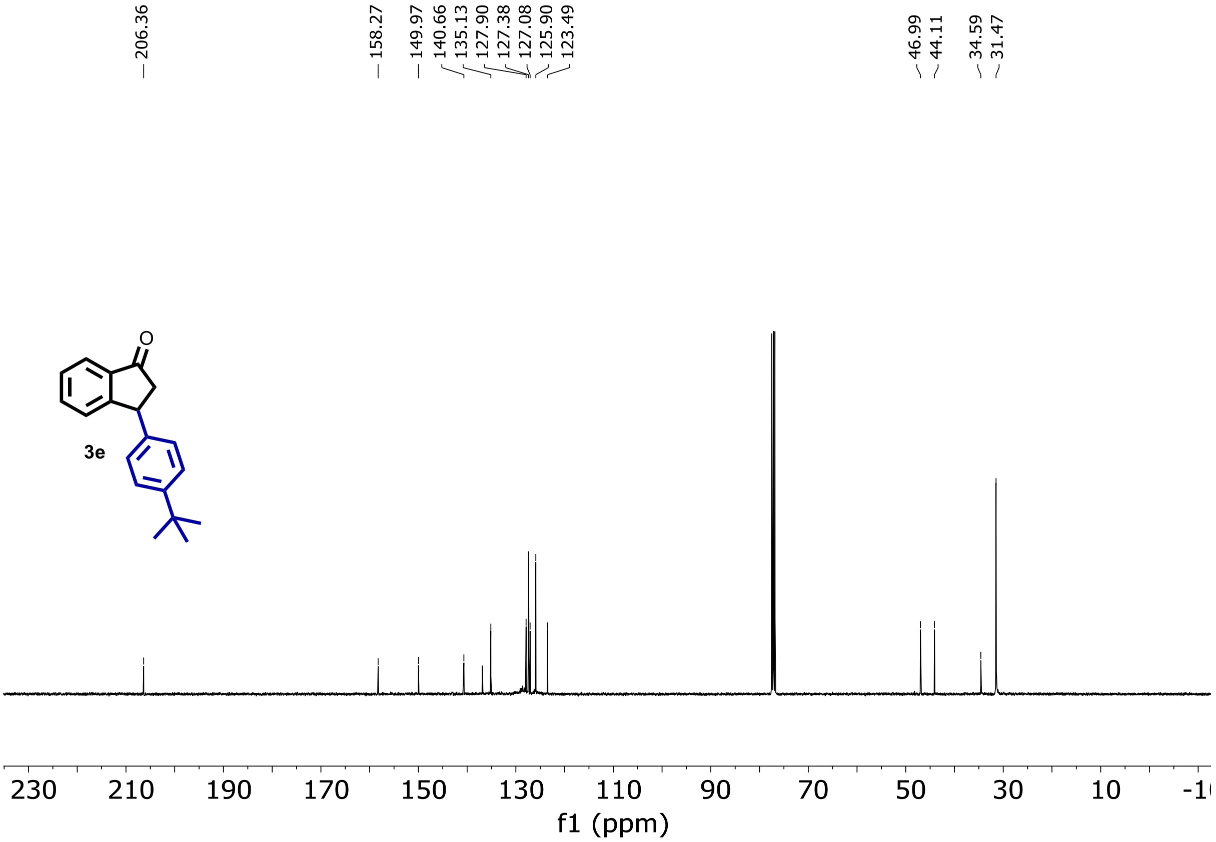

3-(3-chlorophenyl)-2,3-dihydro-1H-inden-1-one (3f): ^{1}H NMR (400 MHz), CDCl$_3$.

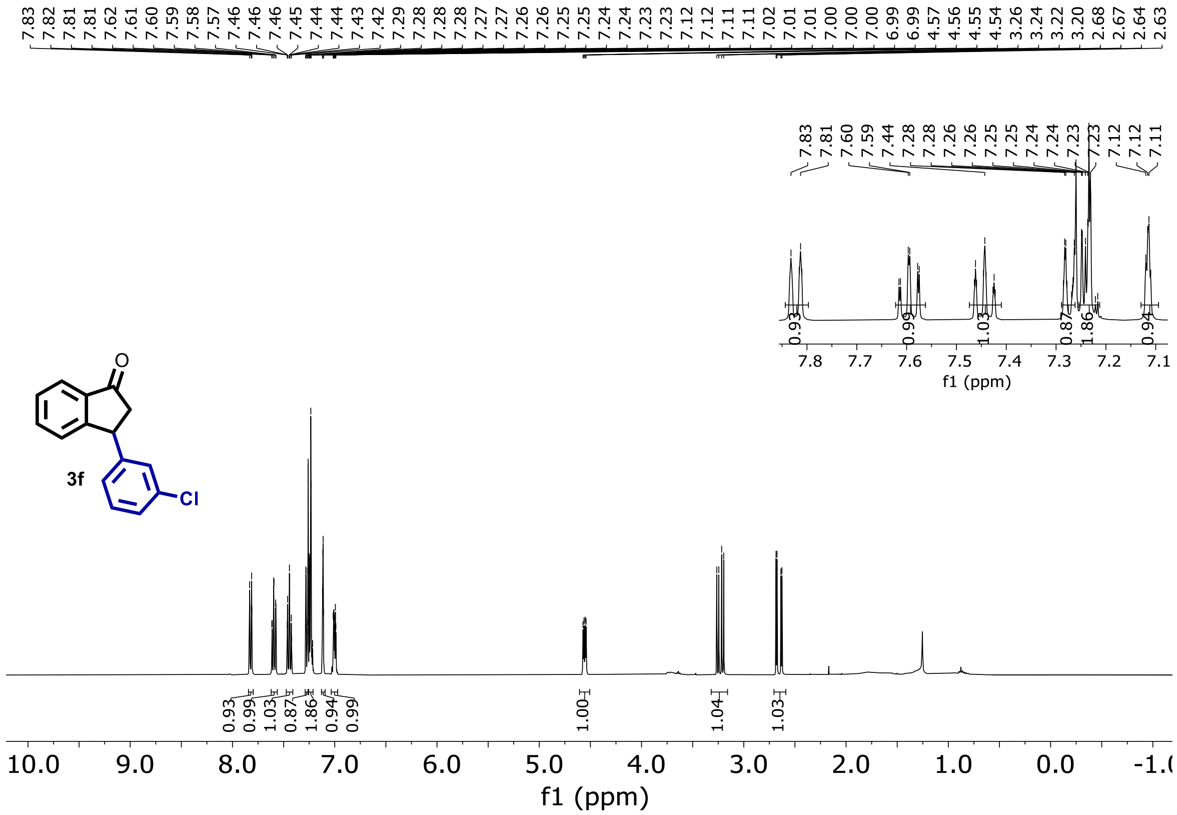

3-(3-chlorophenyl)-2,3-dihydro-1H-inden-1-one (3f): ^{13}C{^{1}H} NMR (101 MHz), CDCl$_3$.

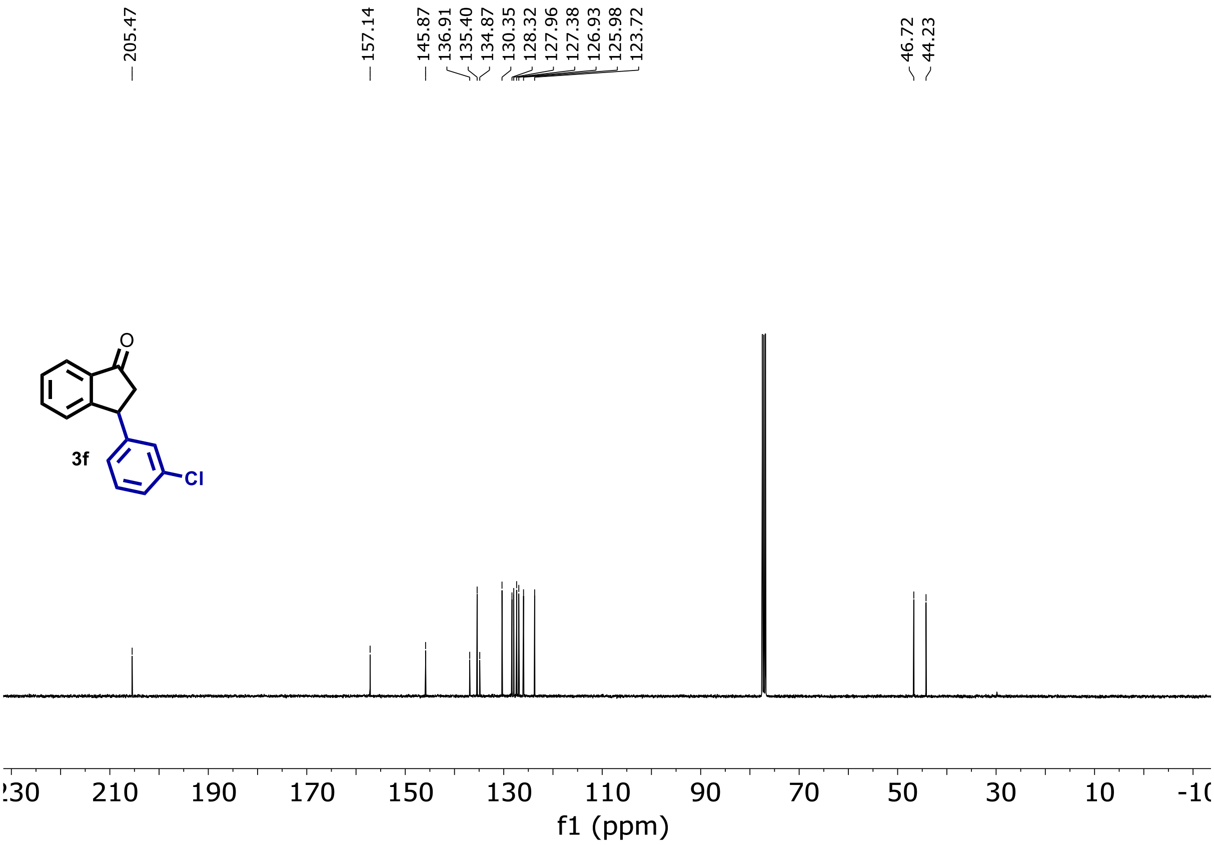

3-(4-chlorophenyl)-2,3-dihydro-1H-inden-1-one (3g): ^{1}H NMR (400 MHz), $CDCl_3$.

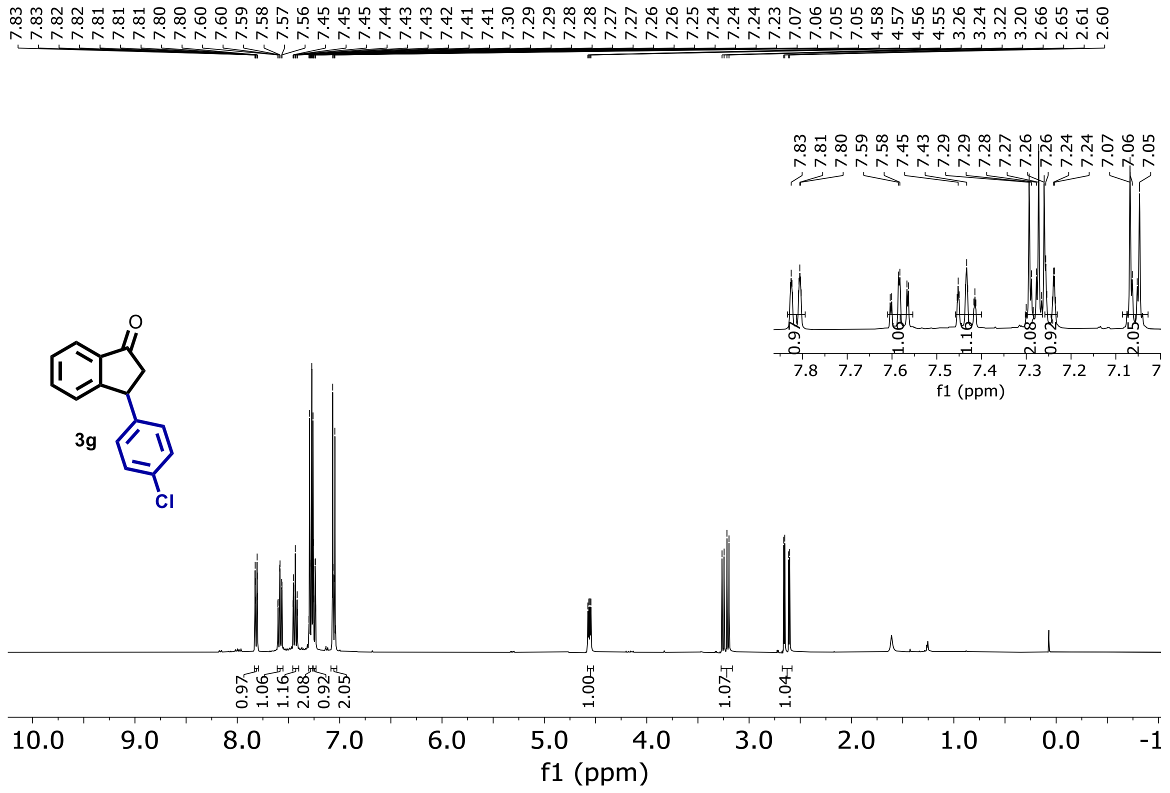

3-(4-chlorophenyl)-2,3-dihydro-1H-inden-1-one (3g): $^{13}C\{^1H\}$ NMR (101 MHz), $CDCl_3$.

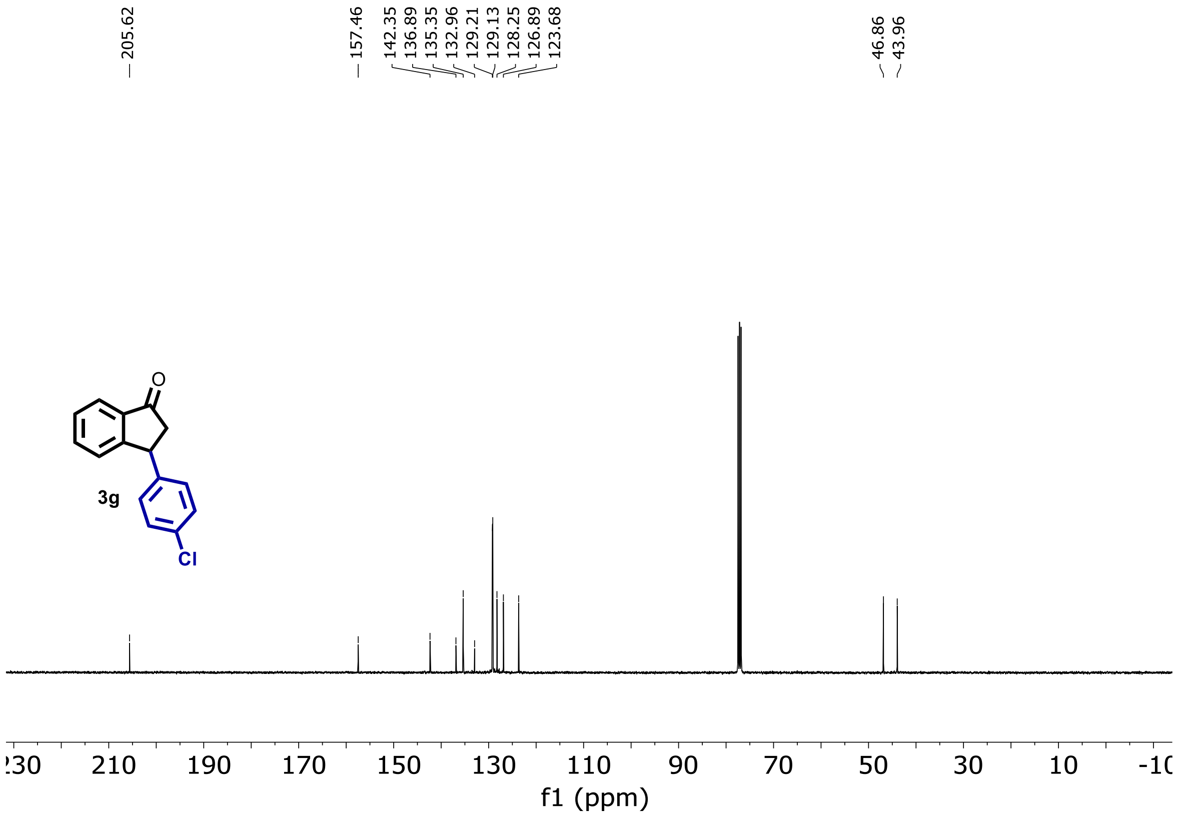

3-(2-fluorophenyl)-2,3-dihydro-1H-inden-1-one (3h): ^{1}H NMR (400 MHz), $CDCl_3$.

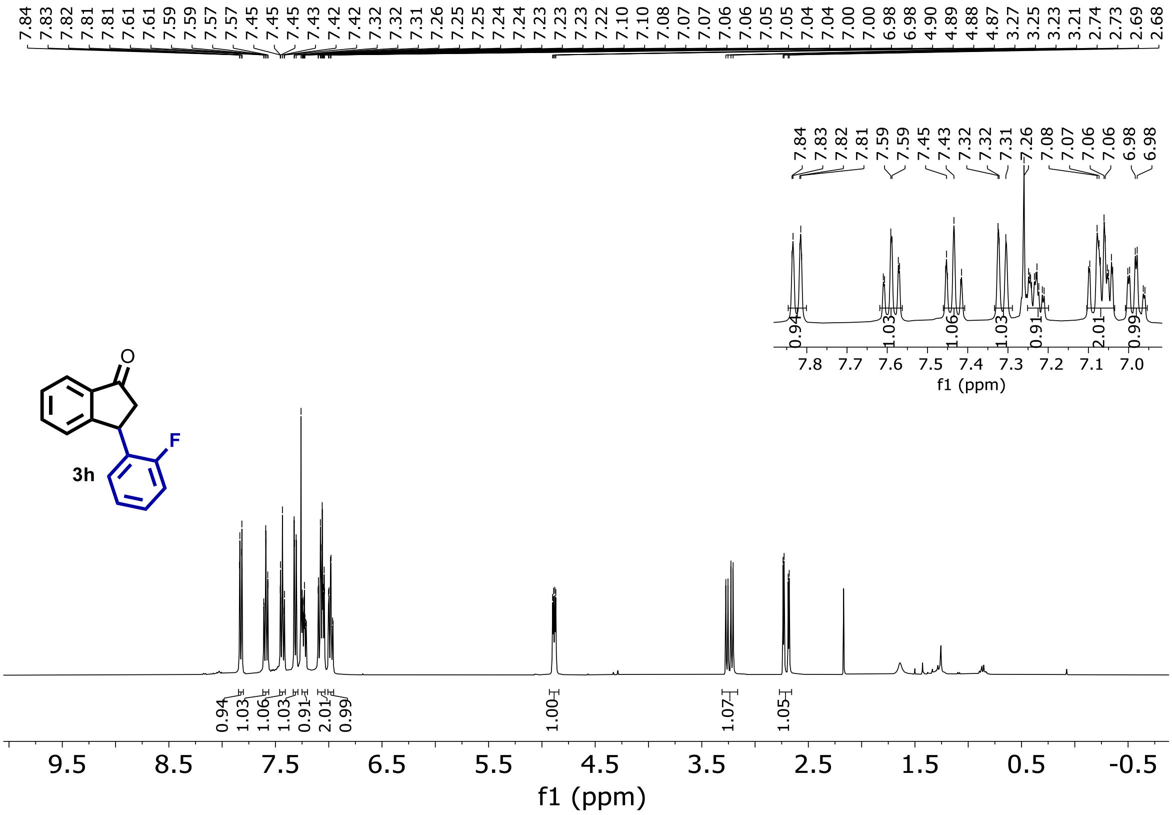

3-(2-fluorophenyl)-2,3-dihydro-1H-inden-1-one (3h): ^{19}F NMR (377 MHz), $CDCl_3$

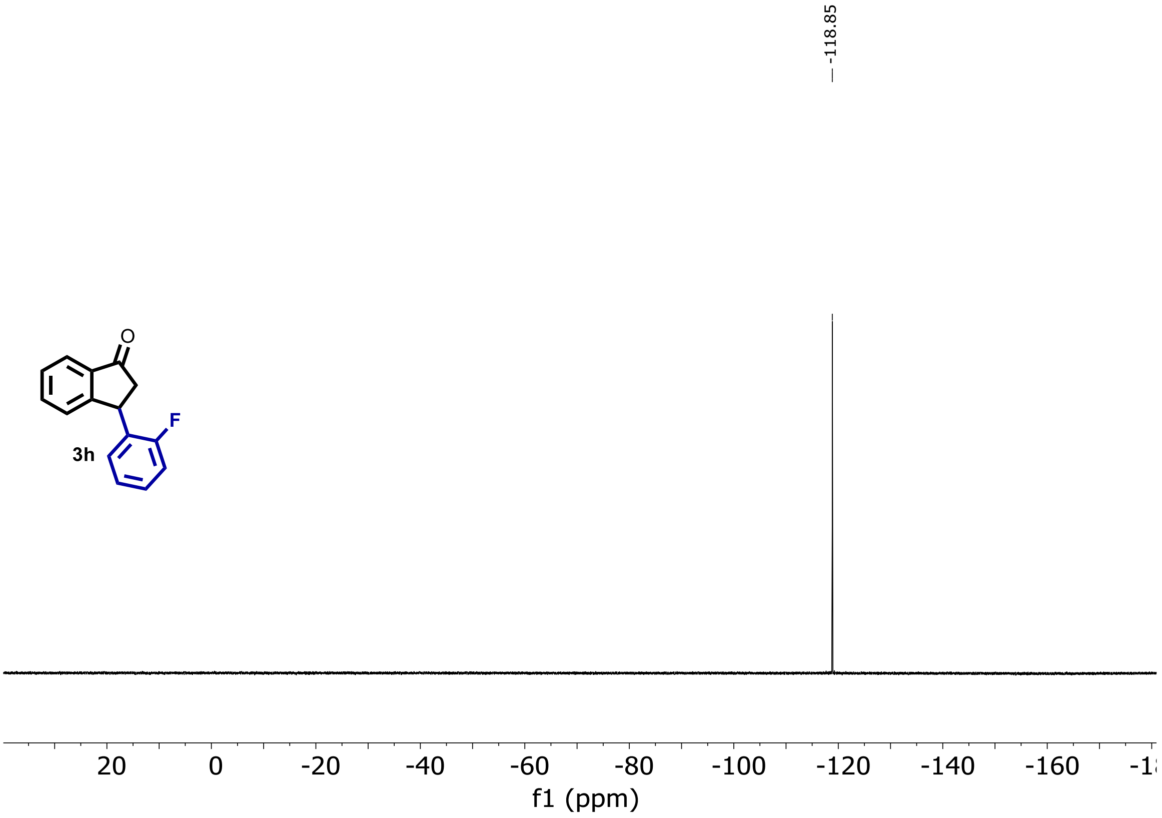

3-(2-fluorophenyl)-2,3-dihydro-1H-inden-1-one (3h): $^{13}C\{^1H\}$ NMR (101 MHz), $CDCl_3$.

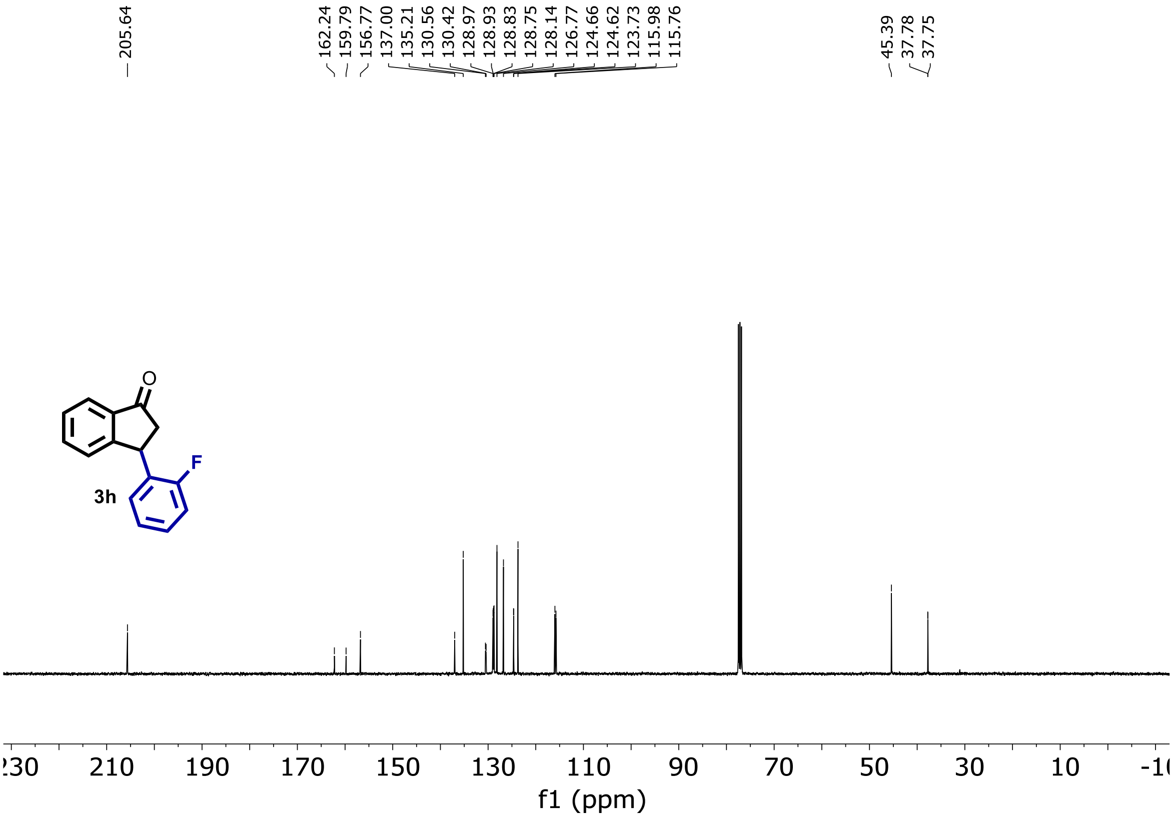

3-(4-fluorophenyl)-2,3-dihydro-1H-inden-1-one (3i): ^{1}H NMR (400 MHz), CDCl$_3$.

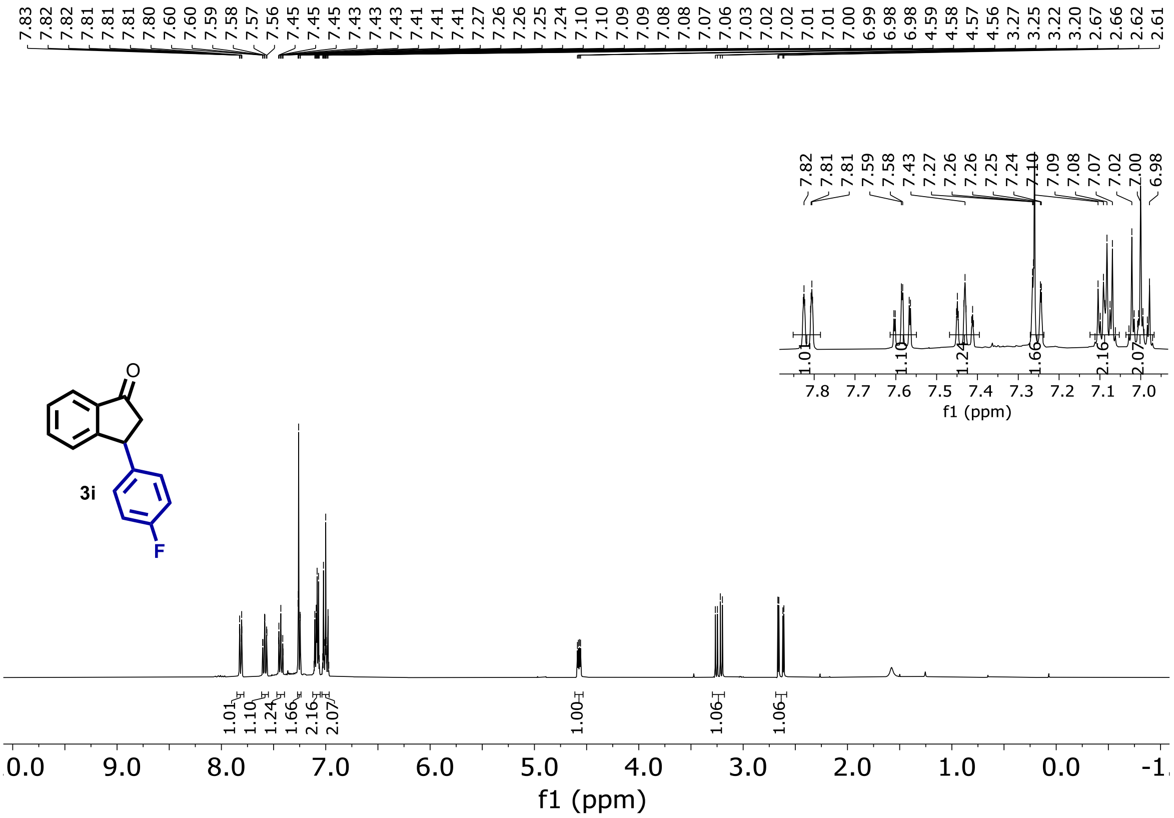

3-(4-fluorophenyl)-2,3-dihydro-1H-inden-1-one (3i): ^{19}F NMR (377 MHz), CDCl$_3$

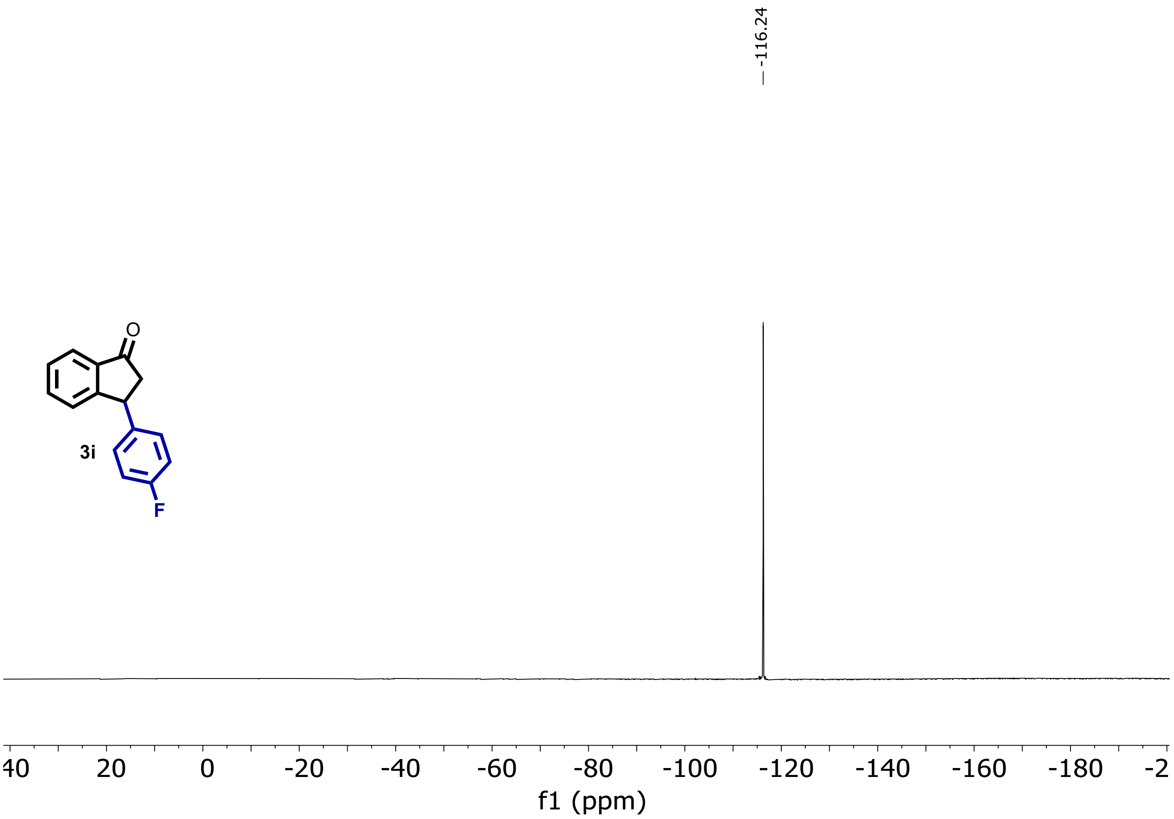

3-(4-fluorophenyl)-2,3-dihydro-1H-inden-1-one (3i): $^{13}C\{^{1}H\}$ NMR (101 MHz), $CDCl_3$.

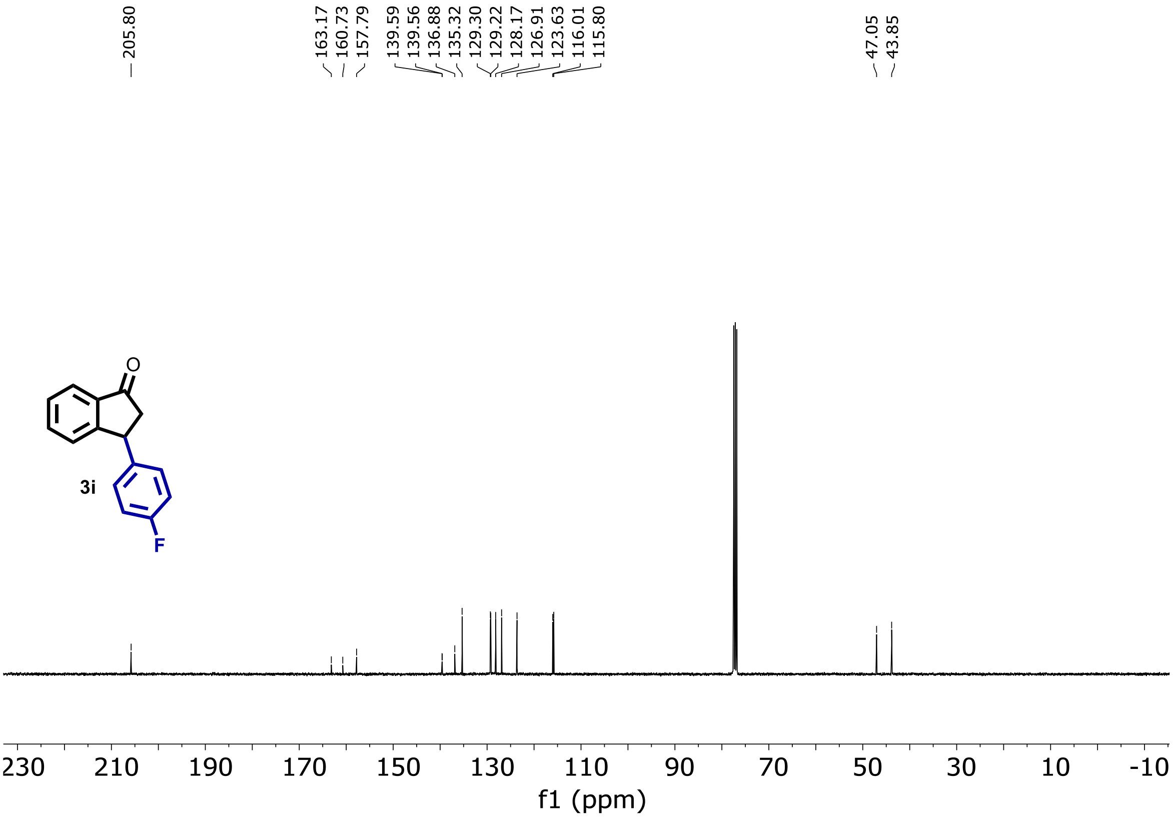

3-(4-(trifluoromethyl)phenyl)-2,3-dihydro-1H-inden-1-one (3j): ^{1}H NMR (400 MHz), $CDCl_3$.

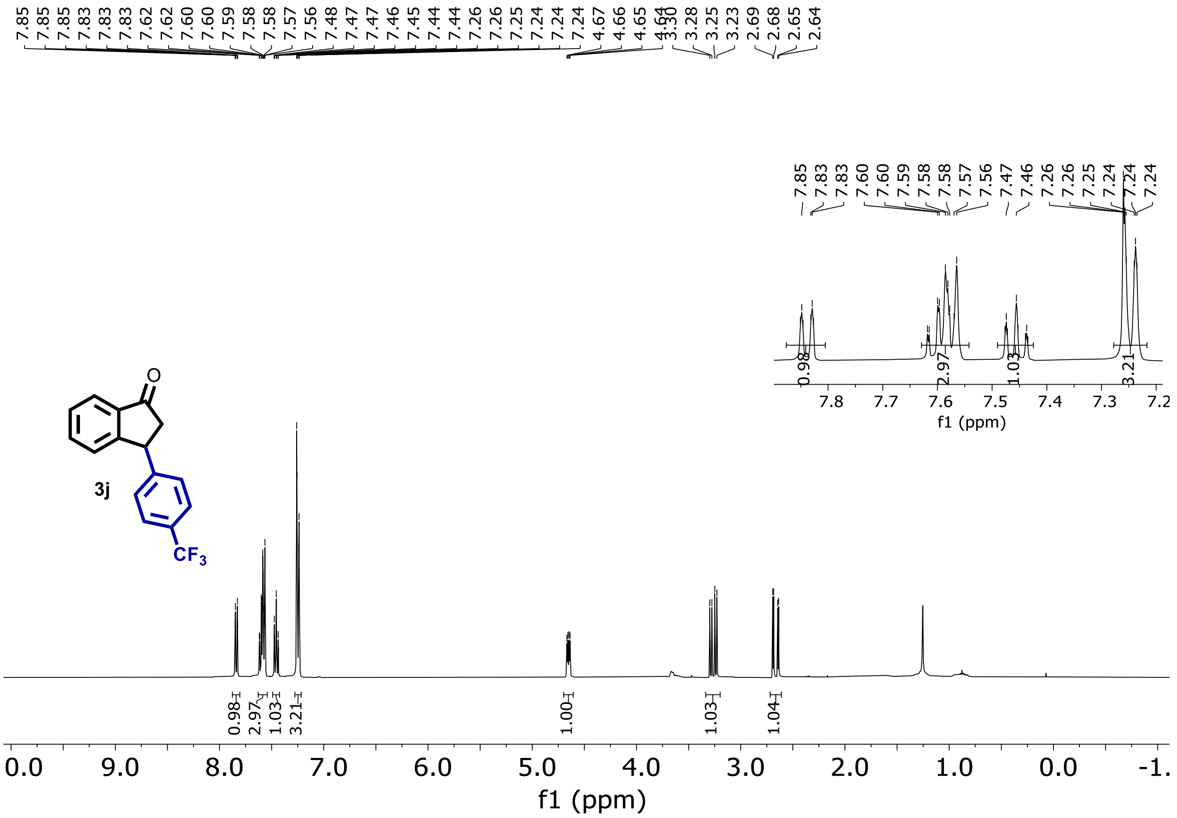

3-(4-(trifluoromethyl)phenyl)-2,3-dihydro-1H-inden-1-one (3j): ^{19}F NMR (377 MHz), $CDCl_3$

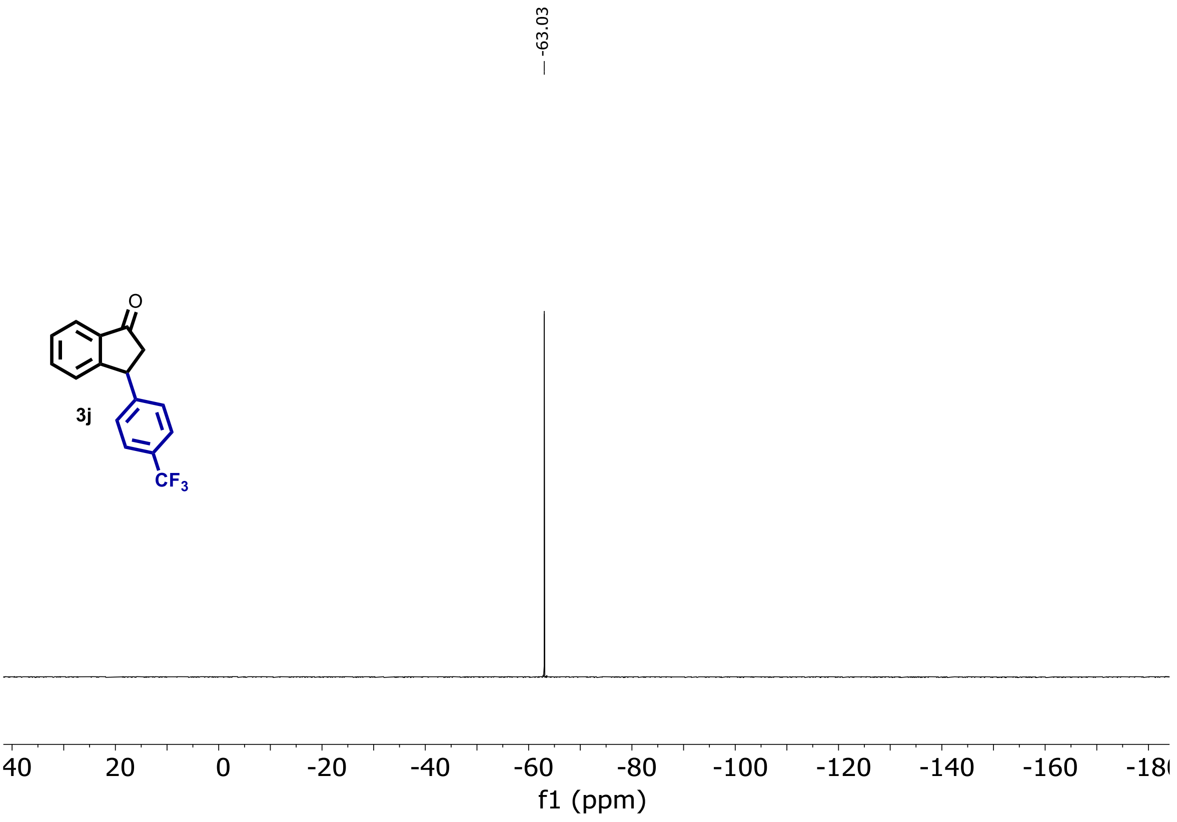

3-(4-(trifluoromethyl)phenyl)-2,3-dihydro-1H-inden-1-one (3j): $^{13}C\{^{1}H\}$ NMR (101 MHz), $CDCl_3$.

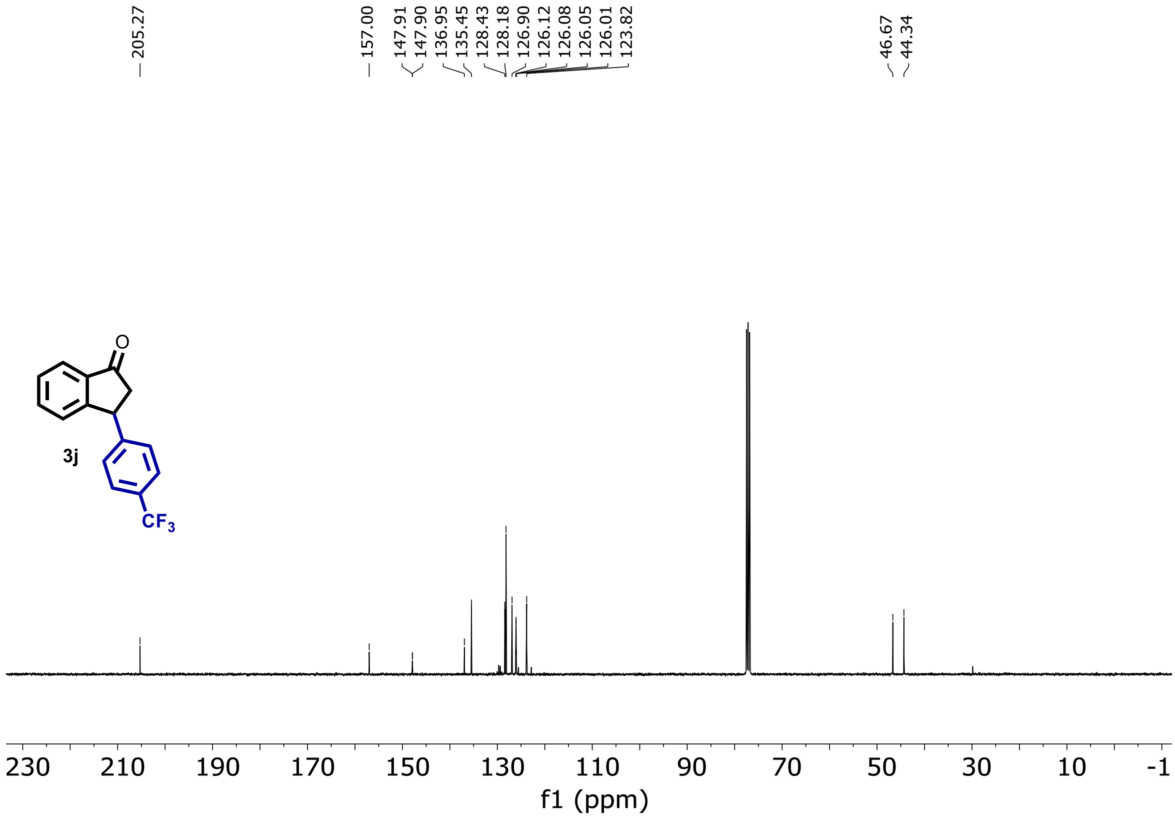

3-(3,5-difluorophenyl)-2,3-dihydro-1H-inden-1-one (3k): ^{1}H NMR (400 MHz), CDCl$_3$.

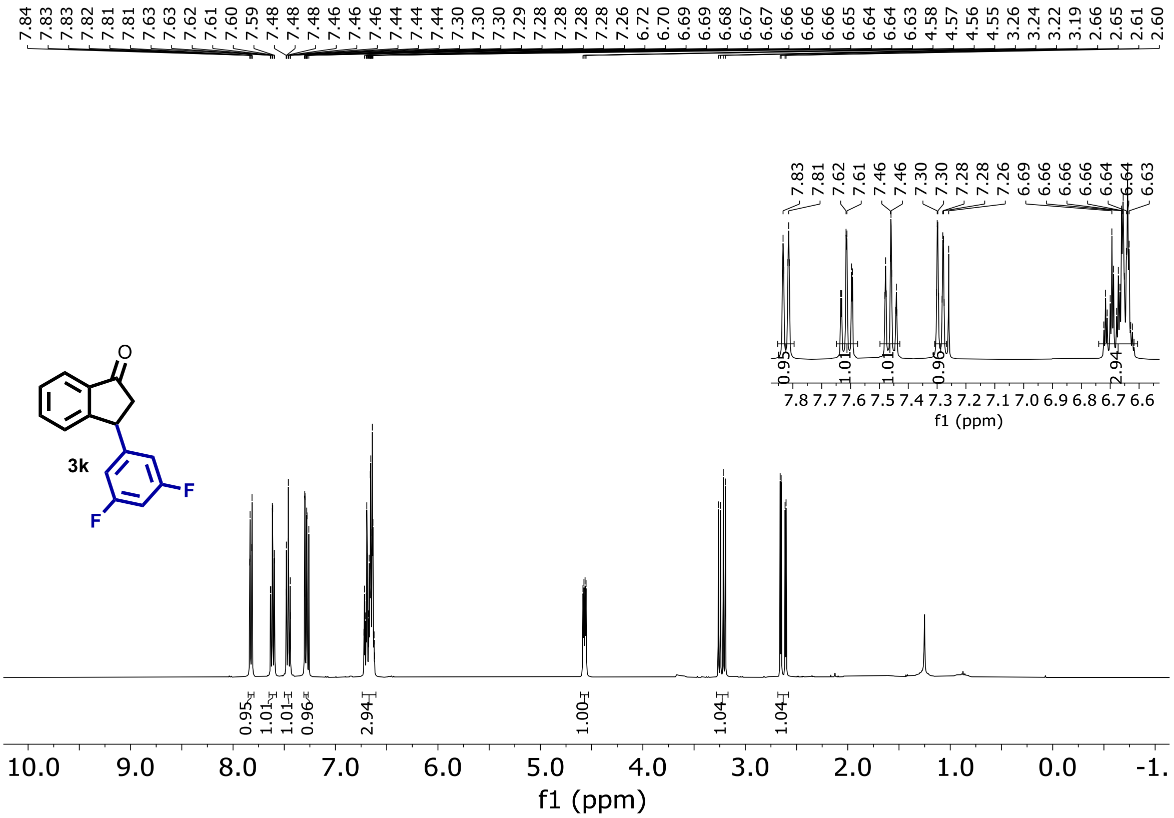

3-(3,5-difluorophenyl)-2,3-dihydro-1H-inden-1-one (3k): ^{19}F NMR (377 MHz), CDCl$_3$

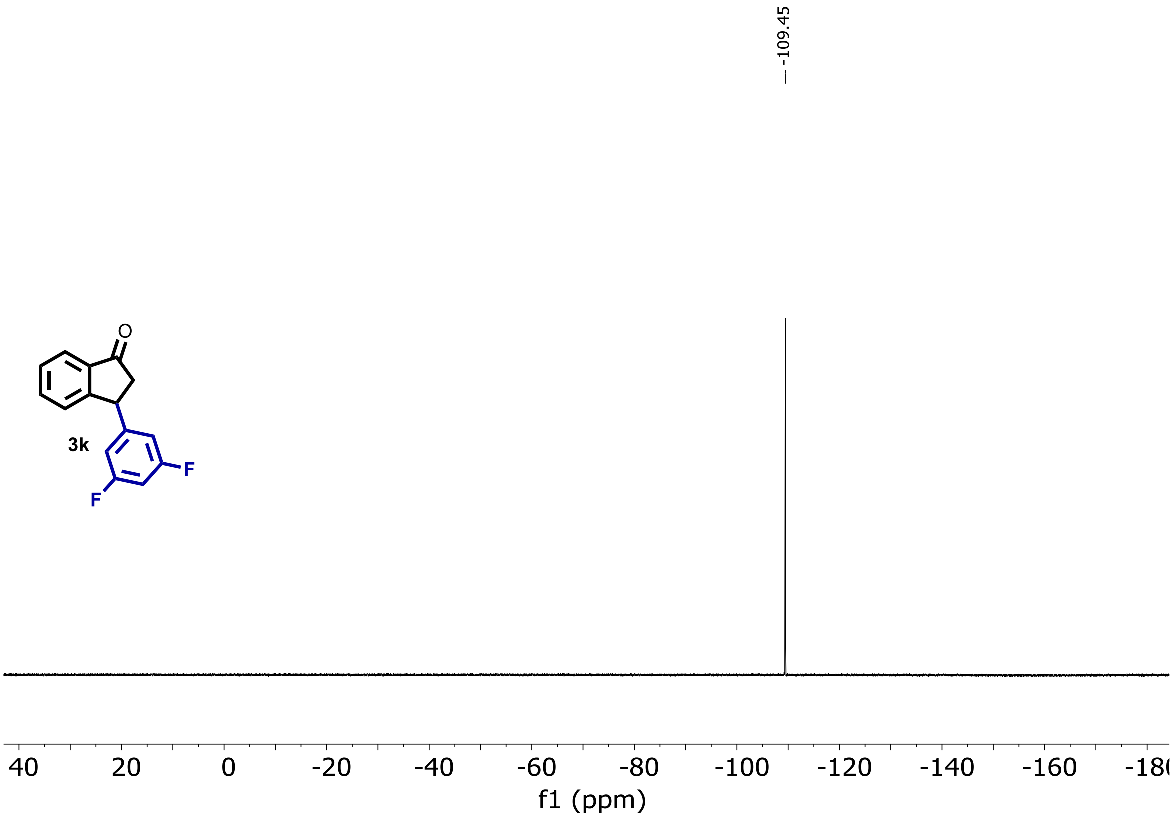

3-(3,5-difluorophenyl)-2,3-dihydro-1H-inden-1-one (3k): $^{13}C\{^{1}H\}$ NMR (101 MHz), $CDCl_3$.

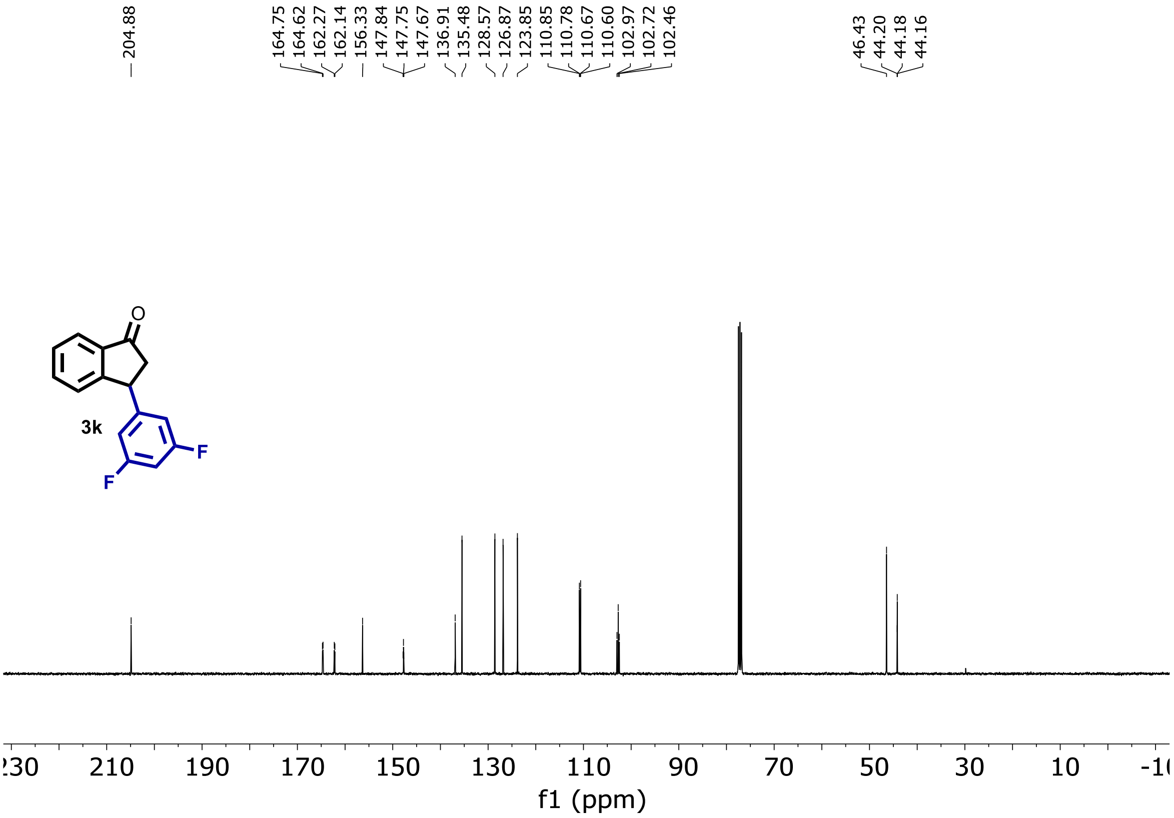

3-(3,5-bis(trifluoromethyl)phenyl)-2,3-dihydro-1H-inden-1-one (3l): ^{1}H NMR (400 MHz), $CDCl_3$.

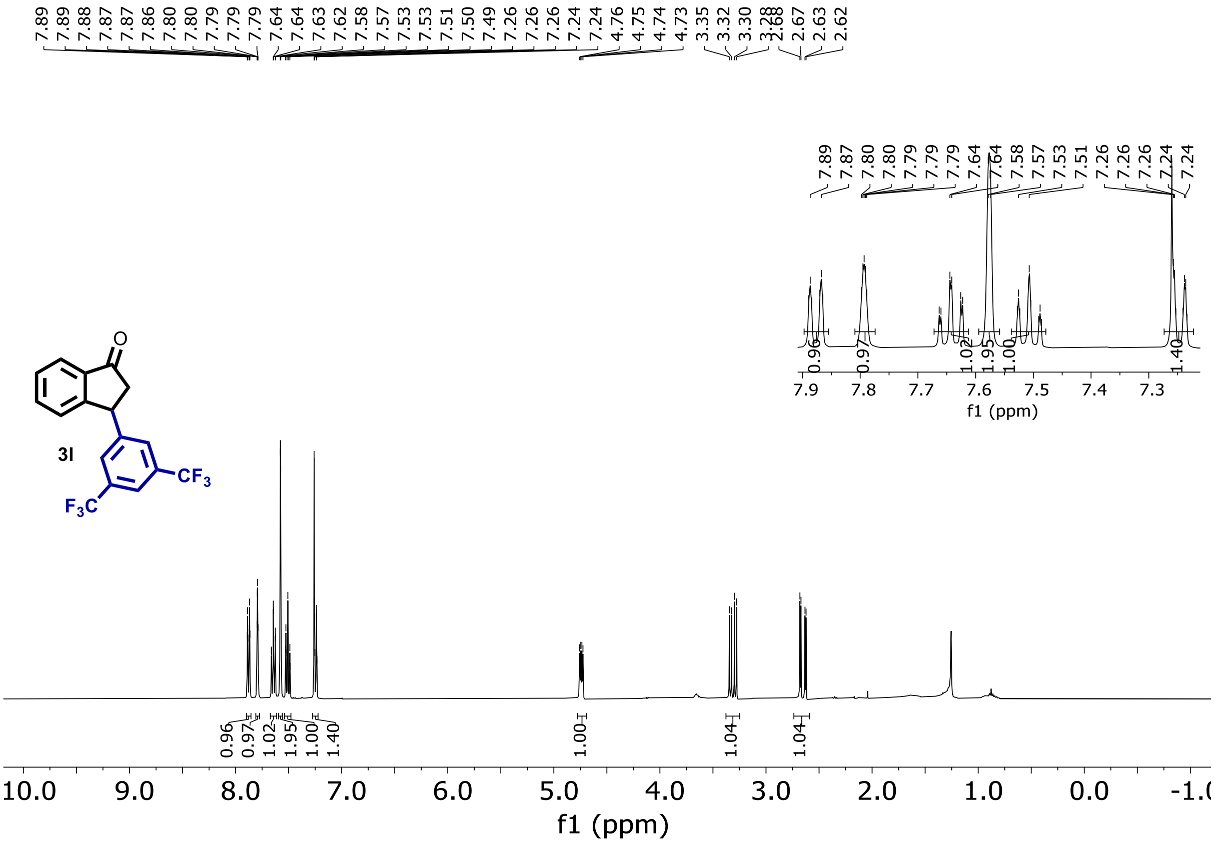

3-(3,5-bis(trifluoromethyl)phenyl)-2,3-dihydro-1H-inden-1-one (3l): ^{19}F NMR (377 MHz), $CDCl_3$

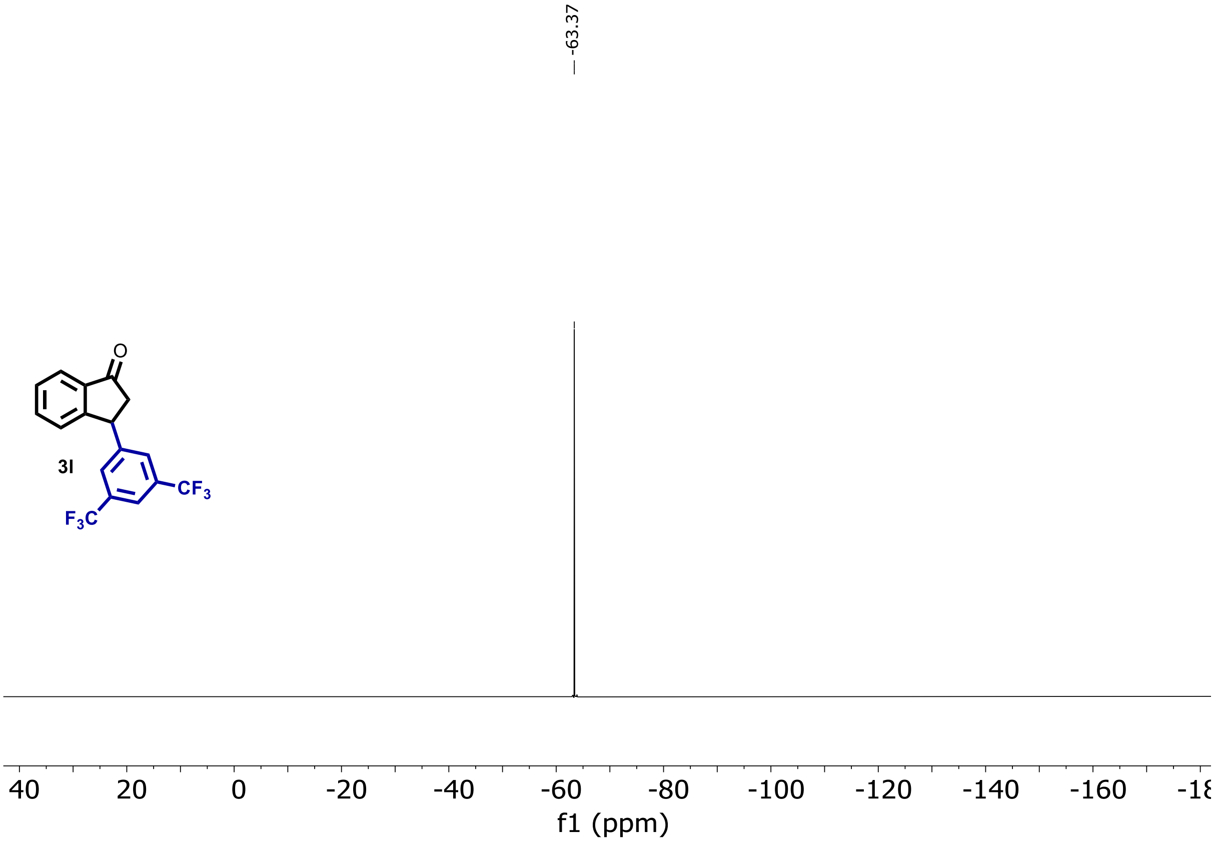

3-(3,5-bis(trifluoromethyl)phenyl)-2,3-dihydro-1H-inden-1-one (3l): $^{13}C\{^{1}H\}$ NMR (101 MHz), $CDCl_3$.

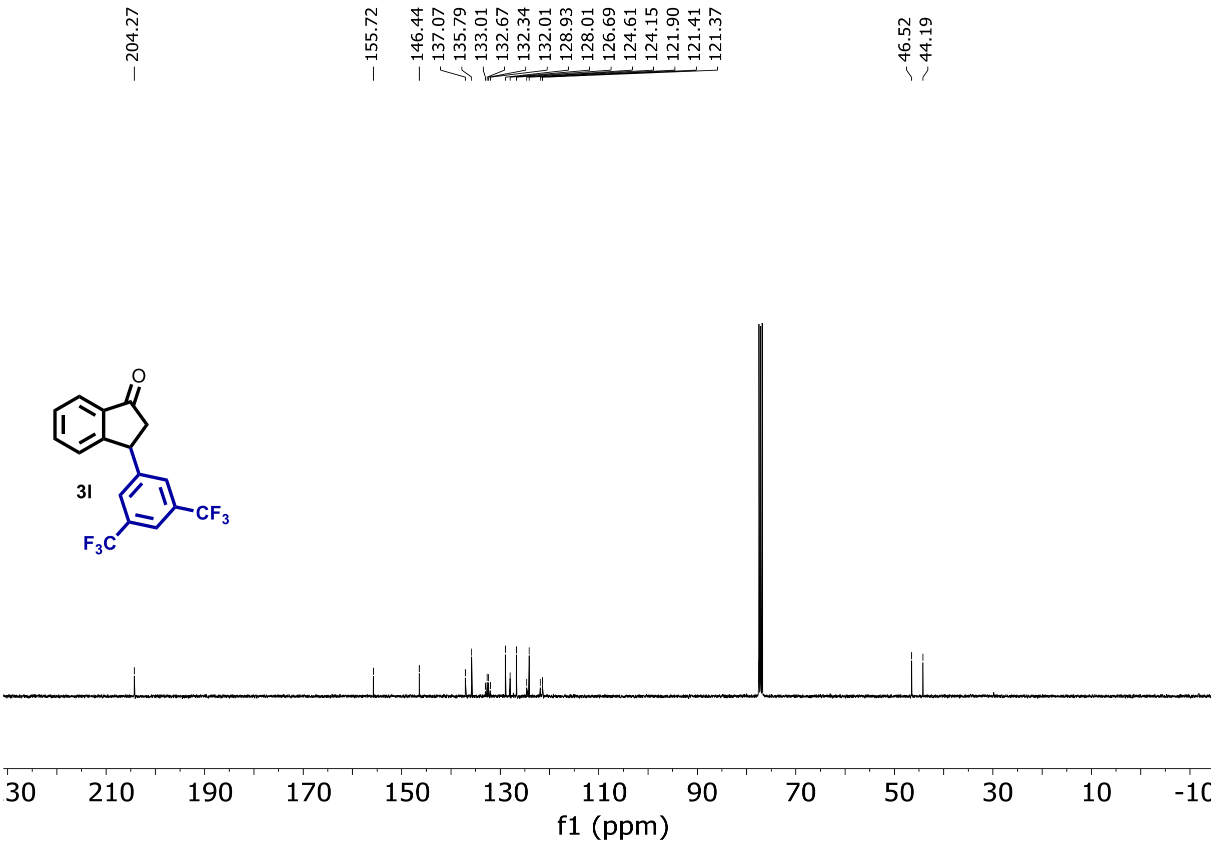

Methyl 4-(3-oxo-2,3-dihydro-1H-inden-1-yl)benzoate (3m): ^{1}H NMR (400 MHz), $CDCl_3$.

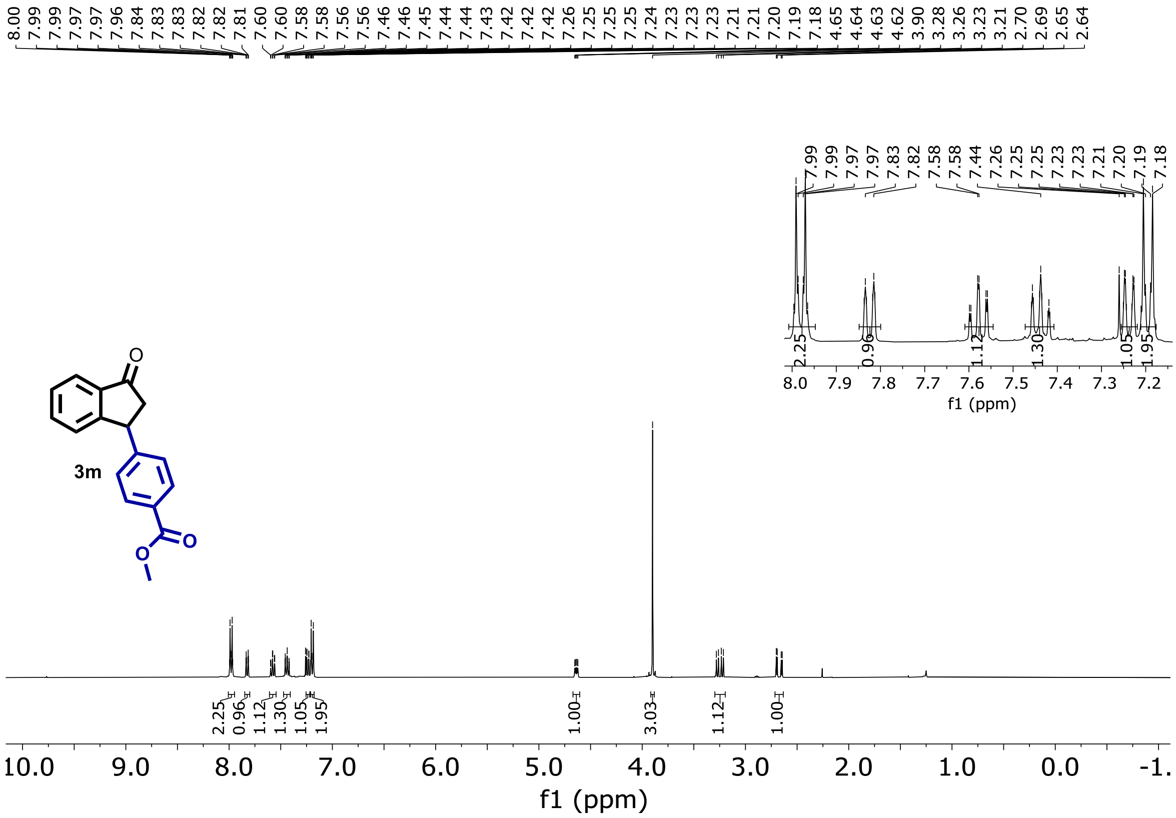

Methyl 4-(3-oxo-2,3-dihydro-1H-inden-1-yl)benzoate (3m): $^{13}C\{^{1}H\}$ NMR (101 MHz), $CDCl_3$.

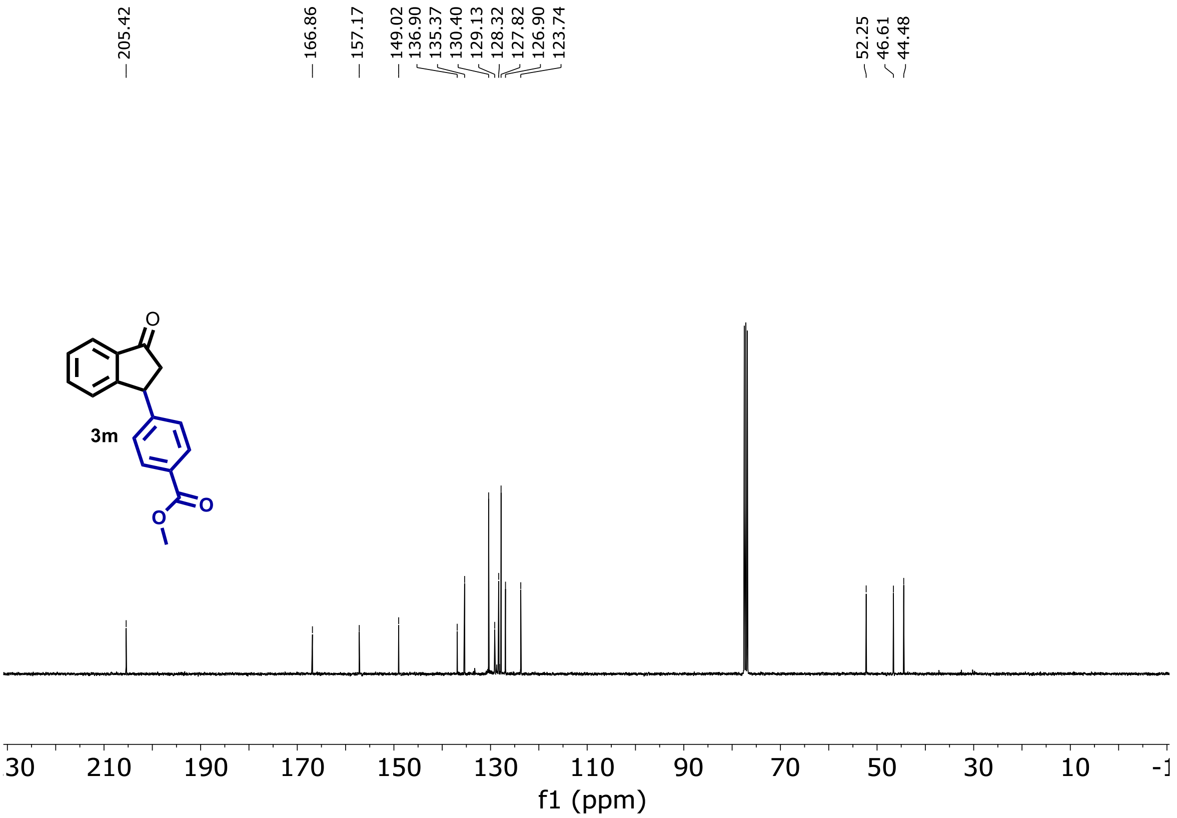

3-(4-(4,4,5,5-tetramethyl-1,3,2-dioxaborolan-2-yl)phenyl)-2,3-dihydro-1H-inden-1-one (3n): ^{1}H NMR (400 MHz), $CDCl_3$.

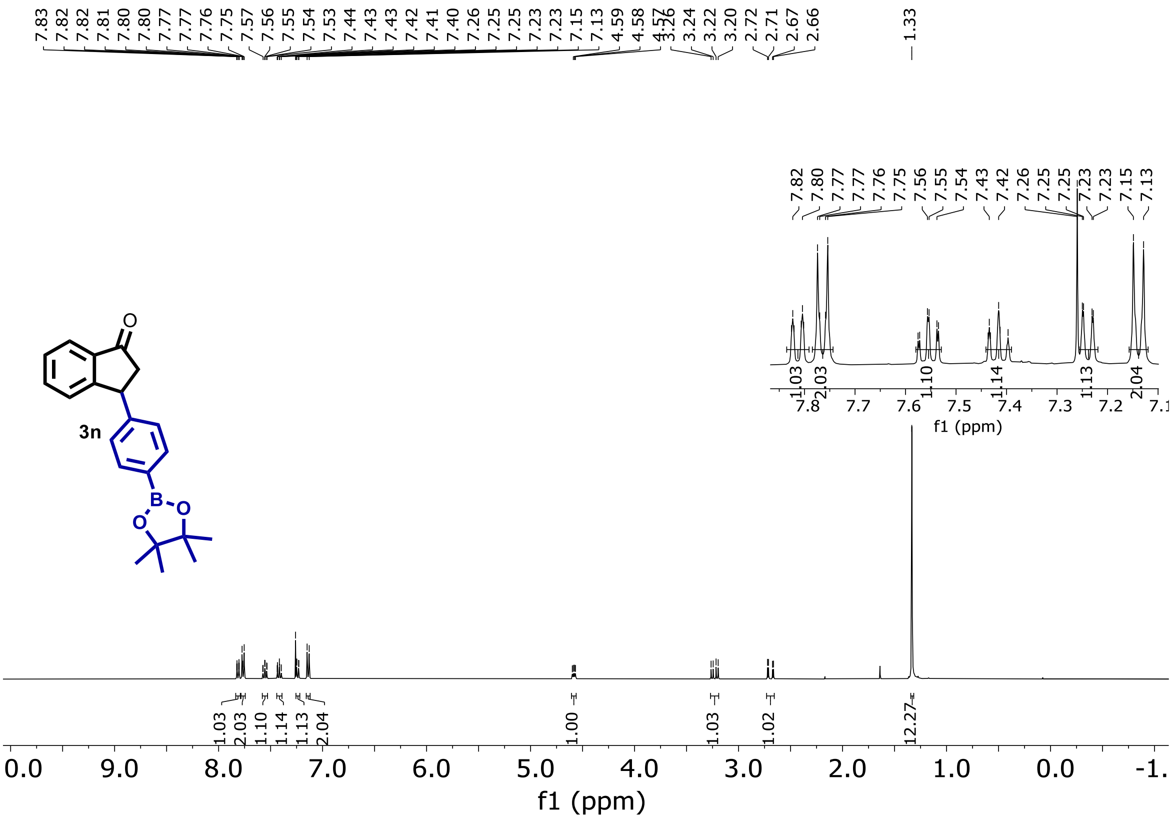

3-(4-(4,4,5,5-tetramethyl-1,3,2-dioxaborolan-2-yl)phenyl)-2,3-dihydro-1H-inden-1-one (3n): ^{11}B NMR (128 MHz), $CDCl_3$.

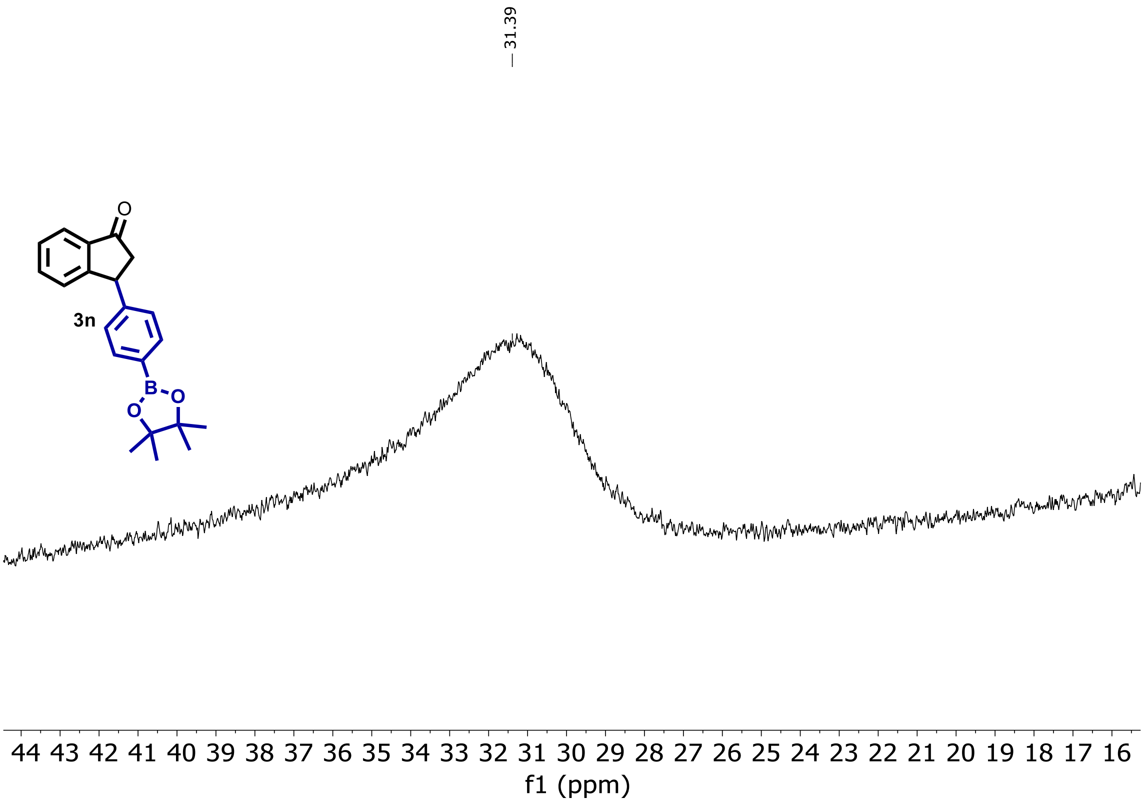

3-(4-(4,4,5,5-tetramethyl-1,3,2-dioxaborolan-2-yl)phenyl)-2,3-dihydro-1H-inden-1-one (3n):
$^{13}C\{^{1}H\}$ NMR (101 MHz), $CDCl_3$.

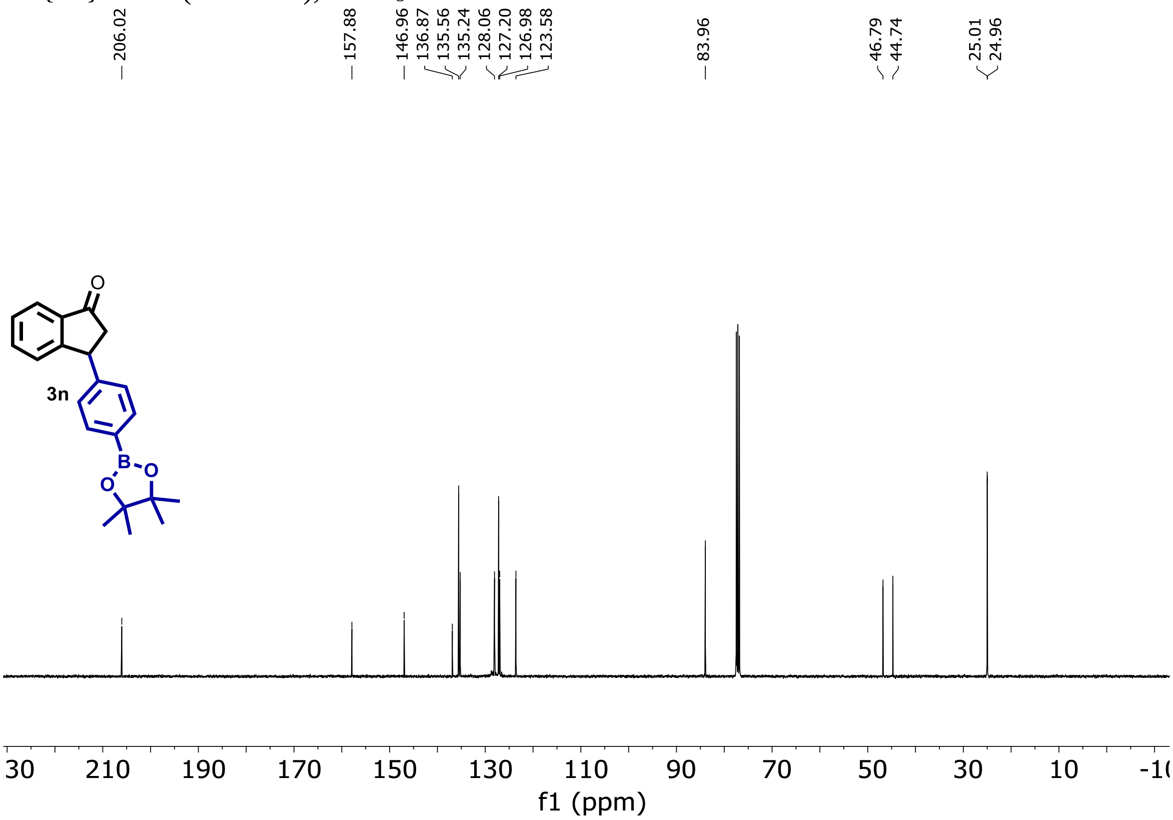

3-(pyridin-2-yl)-2,3-dihydro-1H-inden-1-one (3o): ^{1}H NMR (400 MHz), $CDCl_3$.

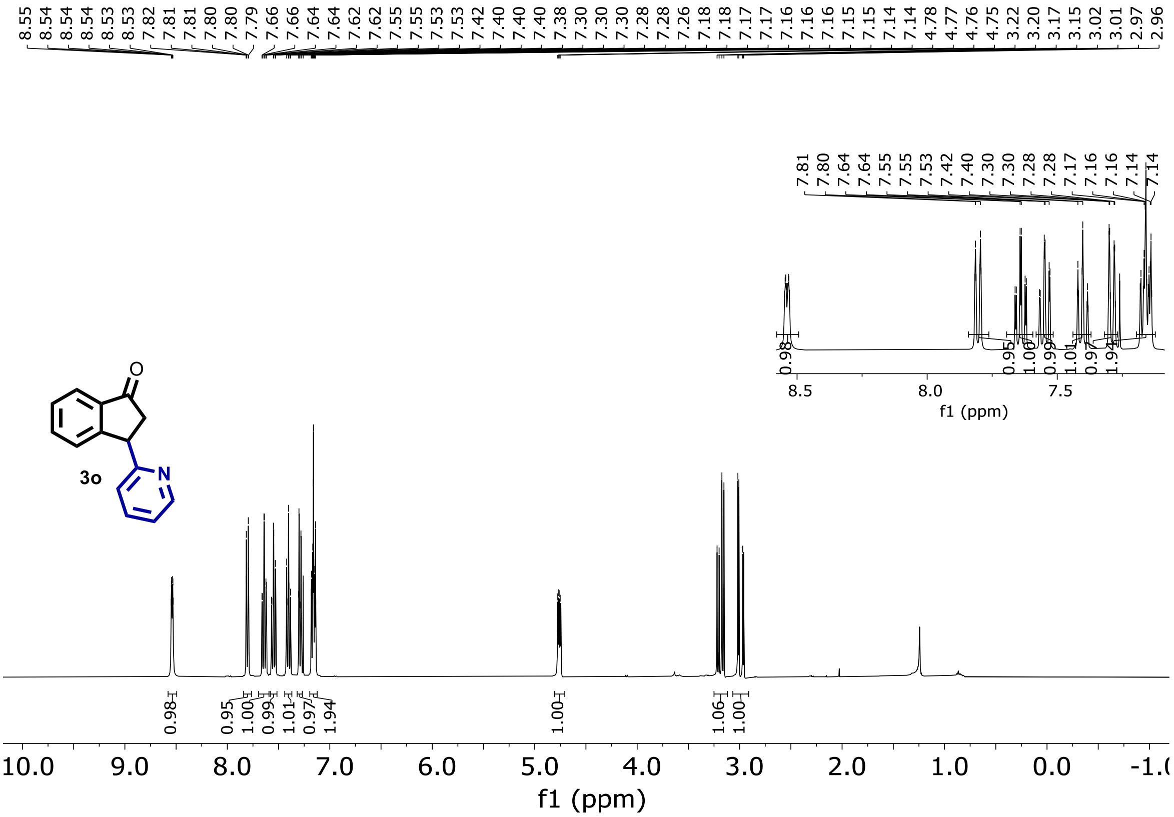

3-(pyridin-2-yl)-2,3-dihydro-1H-inden-1-one (3o): ^{13}C{^{1}H} NMR (101 MHz), $CDCl_3$.

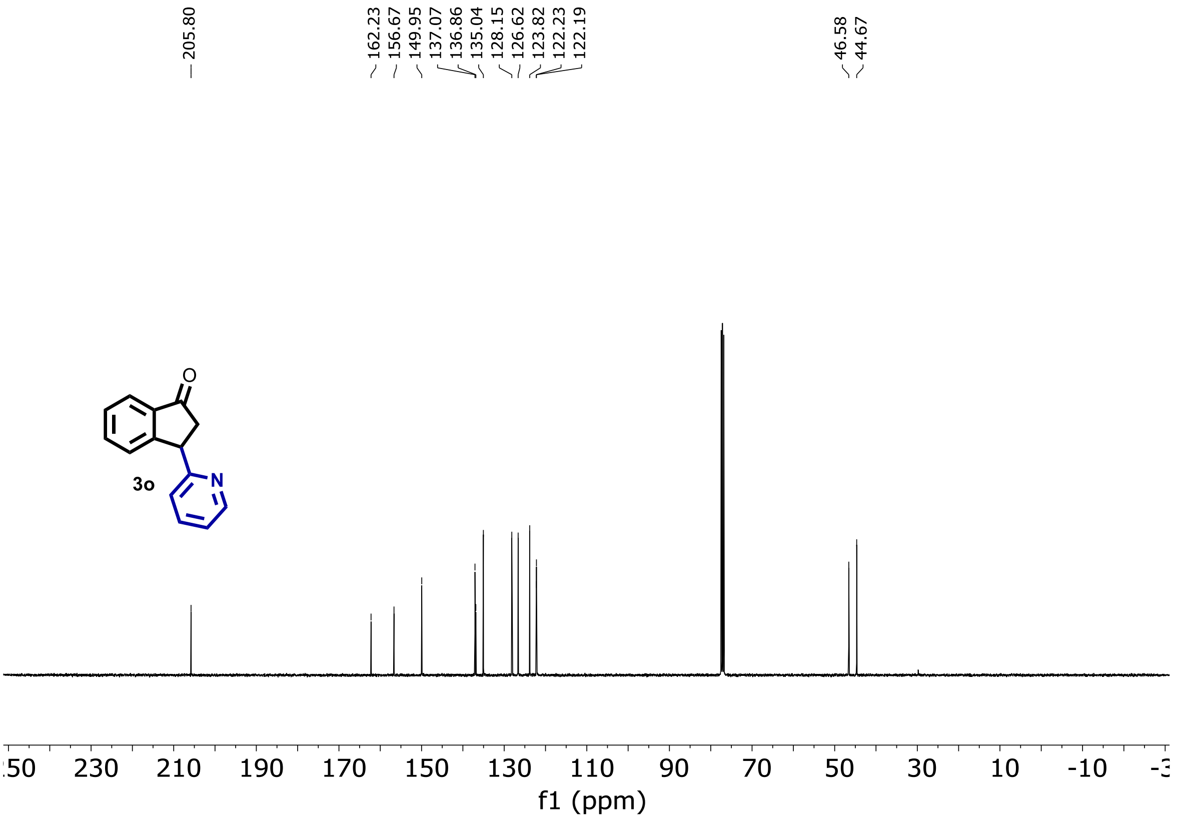

7-methoxy-3-phenyl-2,3-dihydro-1H-inden-1-one (3p): ^{1}H NMR (400 MHz), $CDCl_3$.

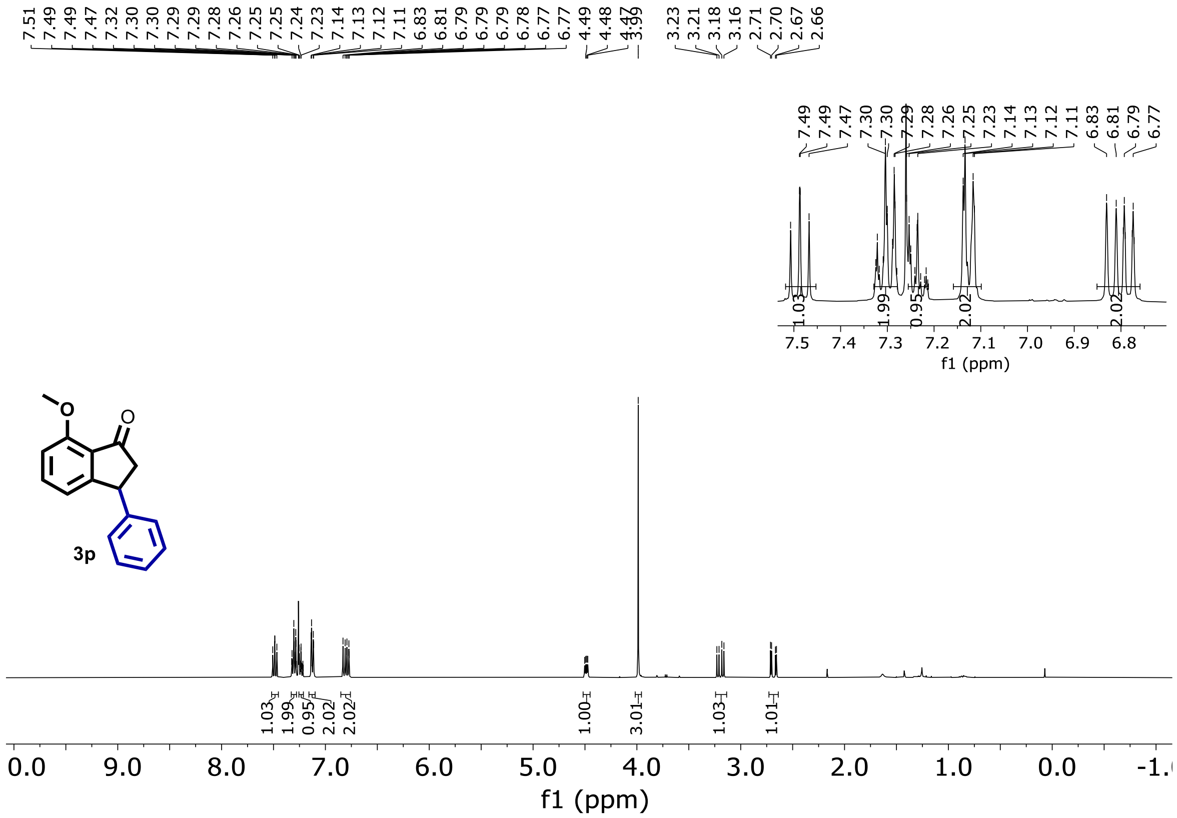

7-methoxy-3-phenyl-2,3-dihydro-1H-inden-1-one (3p): ^{13}C{^{1}H} NMR (101 MHz), $CDCl_3$.

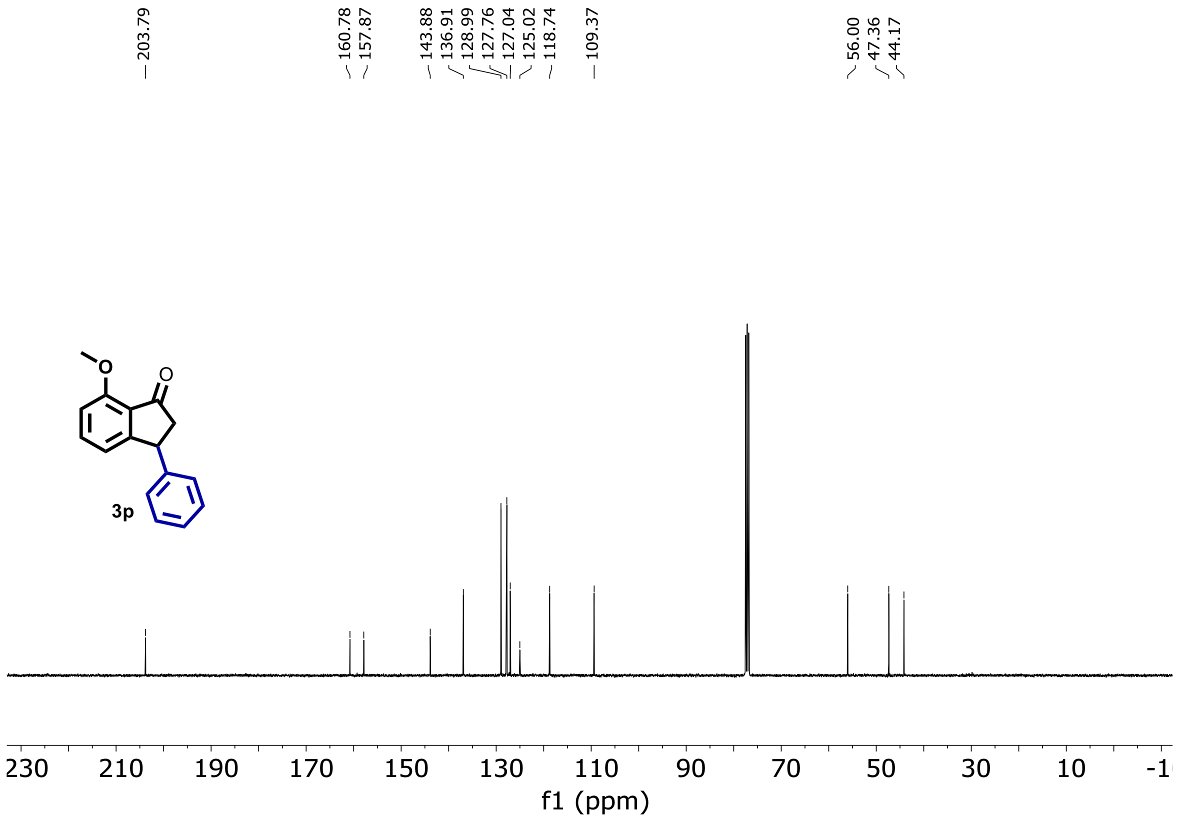

5-methoxy-3-phenyl-2,3-dihydro-1H-inden-1-one (3q): ^{1}H NMR (400 MHz), $CDCl_3$.

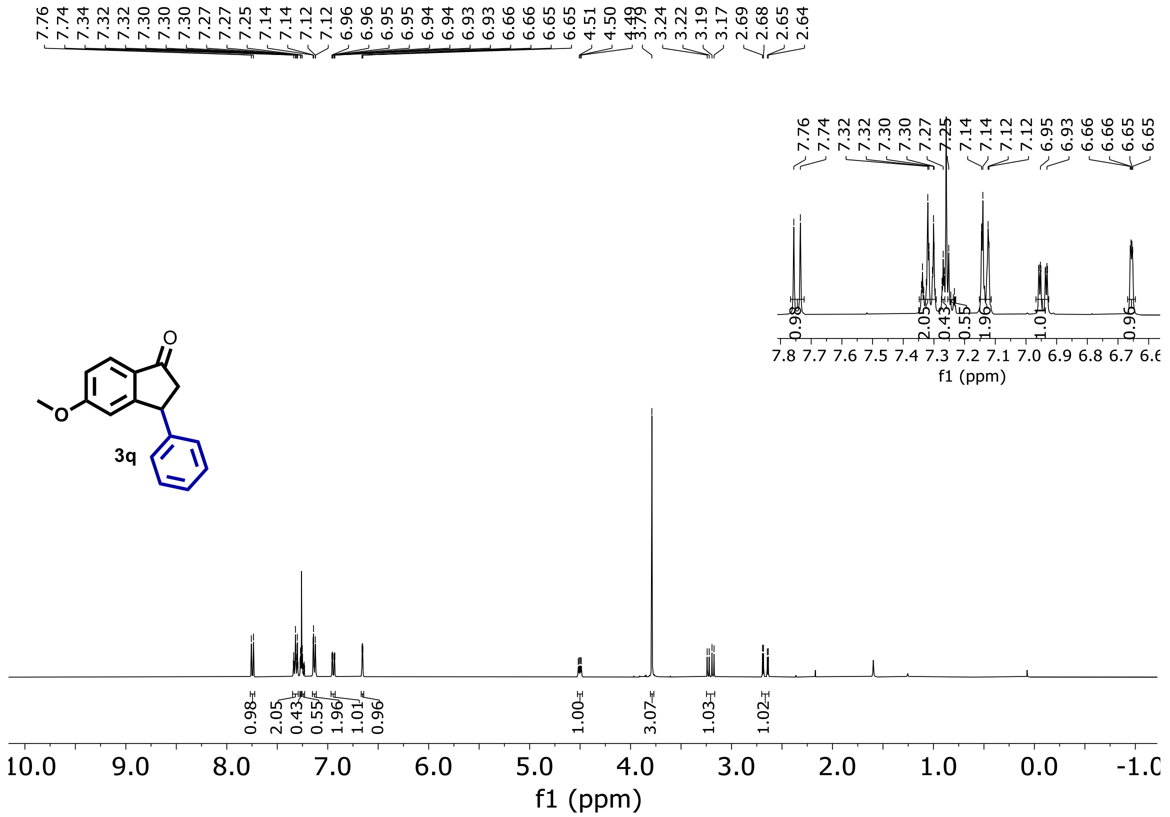

5-methoxy-3-phenyl-2,3-dihydro-1H-inden-1-one (3q): ^{13}C{^{1}H} NMR (101 MHz), $CDCl_3$.

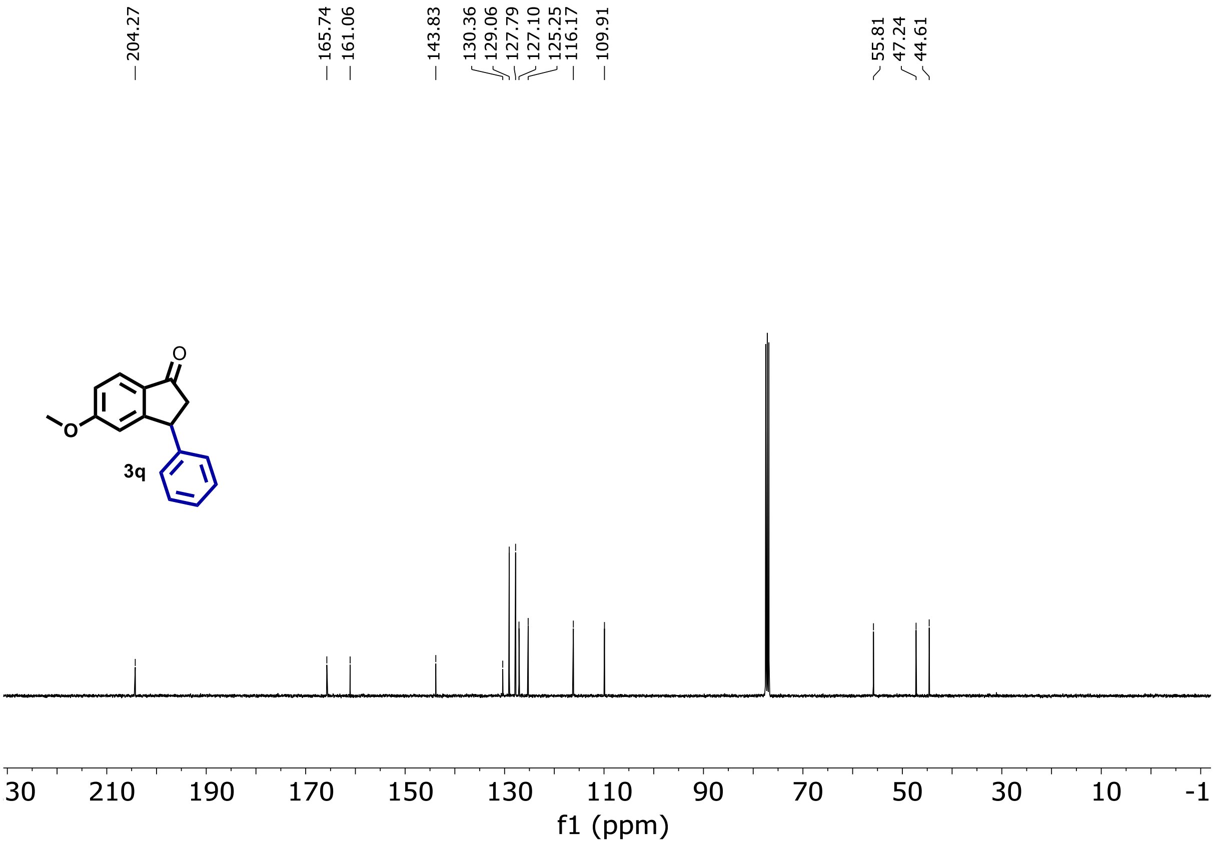

5-isopropoxy-3-phenyl-2,3-dihydro-1H-inden-1-one (3r): ^{1}H NMR (400 MHz), $CDCl_3$.

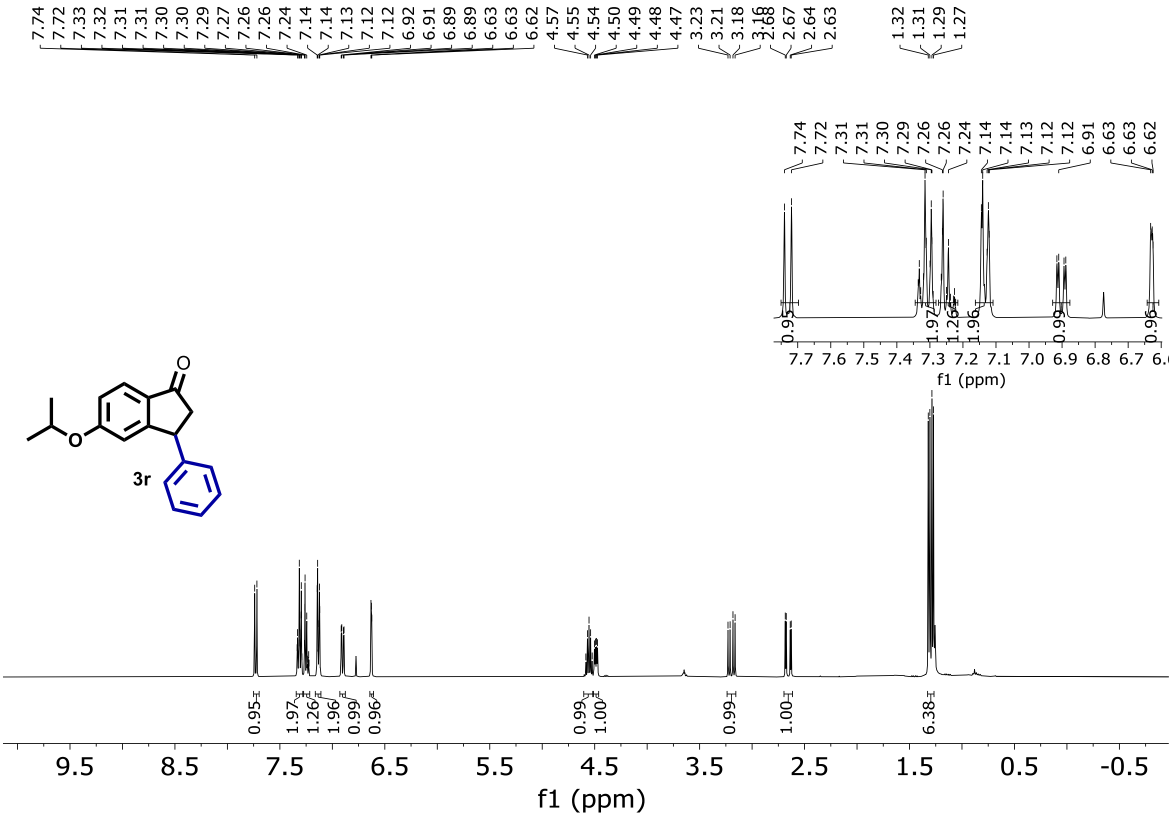

5-isopropoxy-3-phenyl-2,3-dihydro-1H-inden-1-one (3r): $^{13}C\{^{1}H\}$ NMR (101 MHz), $CDCl_3$.

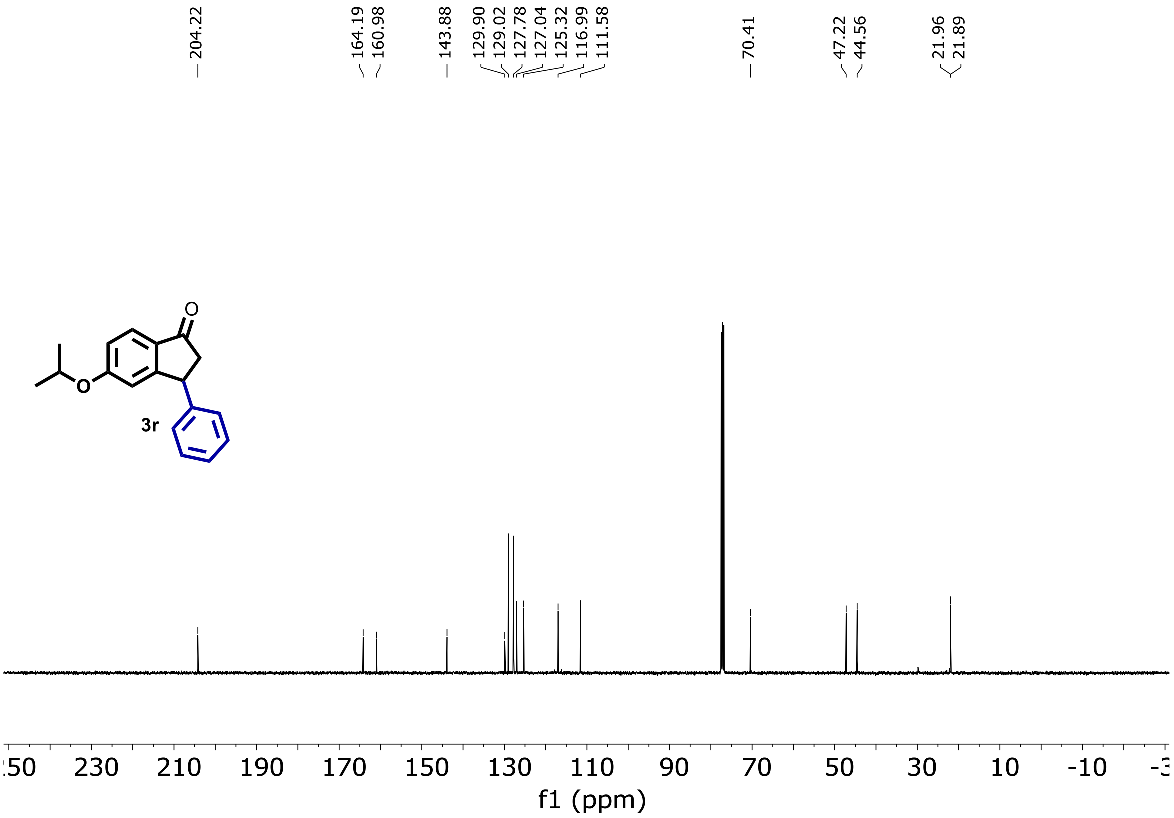

5-(tert-butyl)-3-phenyl-2,3-dihydro-1H-inden-1-one (3s): ^{1}H NMR (400 MHz), $CDCl_3$.

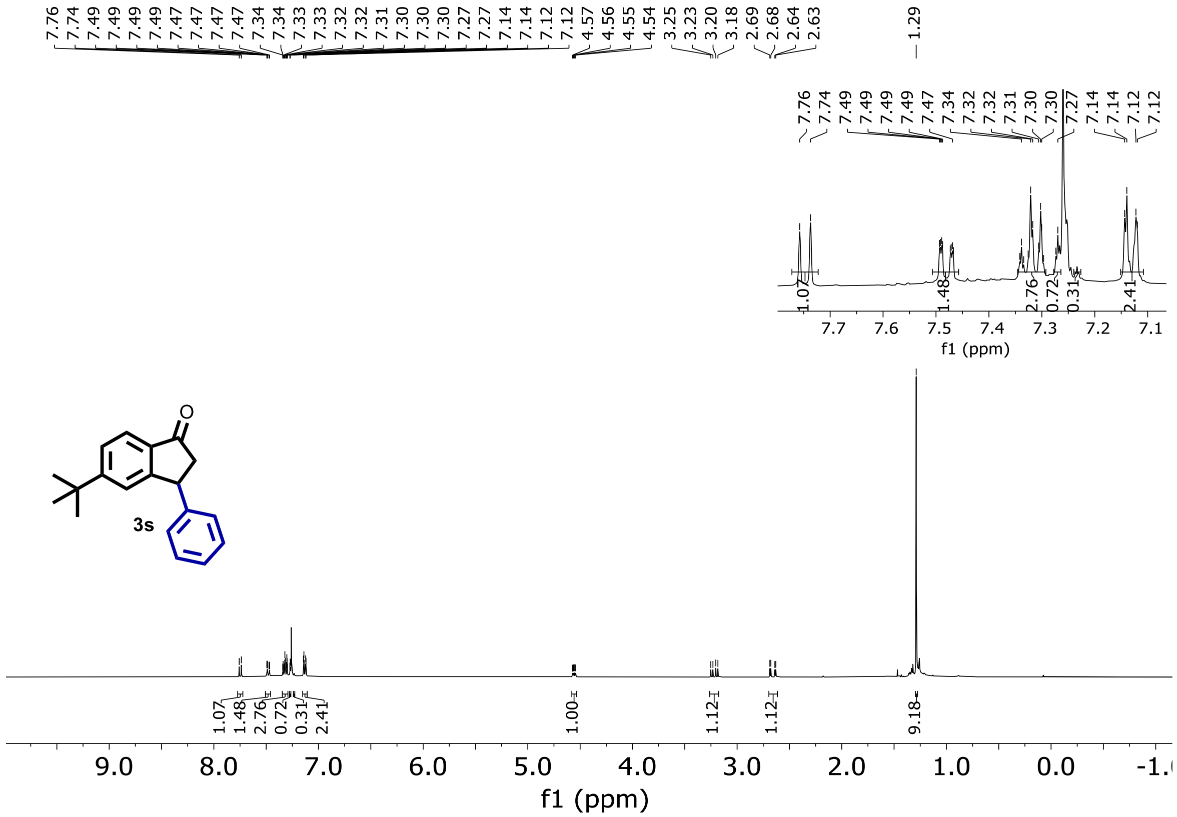

5-(tert-butyl)-3-phenyl-2,3-dihydro-1H-inden-1-one (3s): $^{13}C\{^{1}H\}$ NMR (101 MHz), $CDCl_3$.

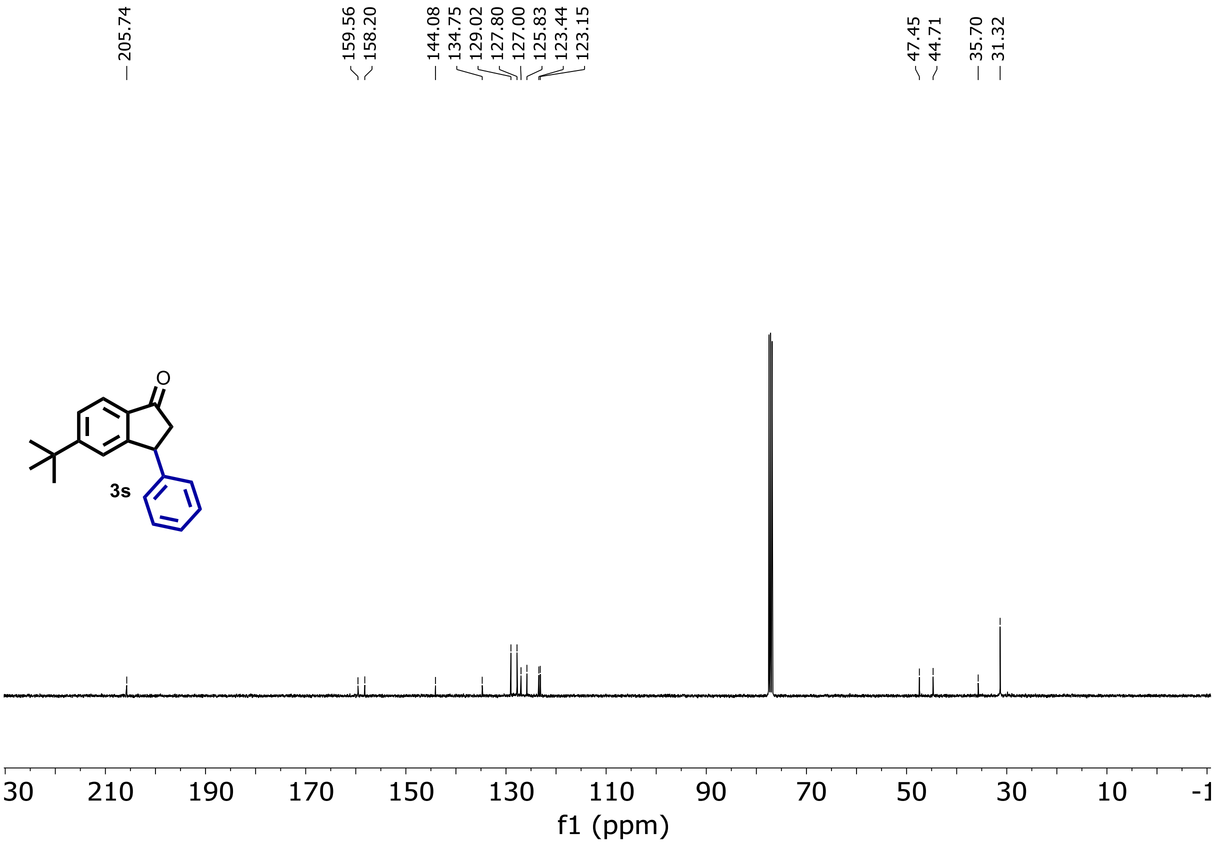

5,7-dimethyl-3-phenyl-2,3-dihydro-1H-inden-1-one (3t): ^{1}H NMR (400 MHz), $CDCl_3$.

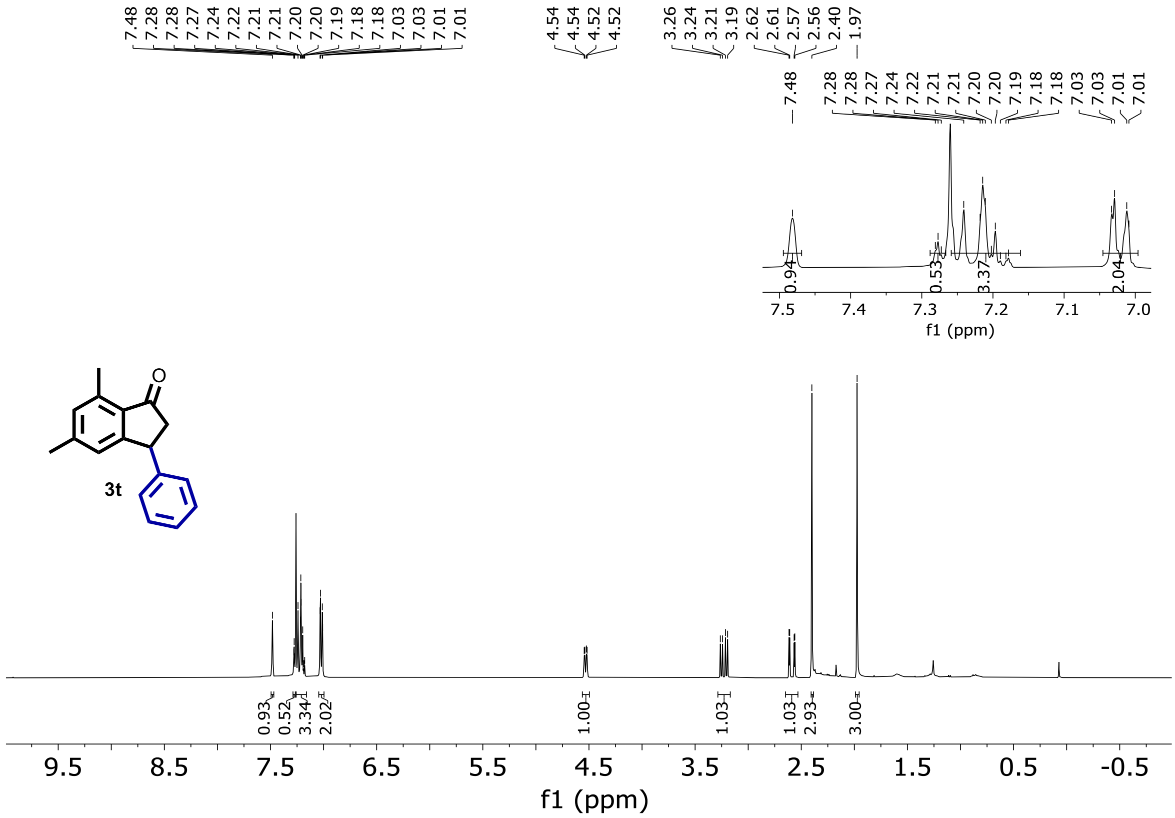

5,7-dimethyl-3-phenyl-2,3-dihydro-1H-inden-1-one (3t): ^{13}C{^{1}H} NMR (101 MHz), $CDCl_3$.

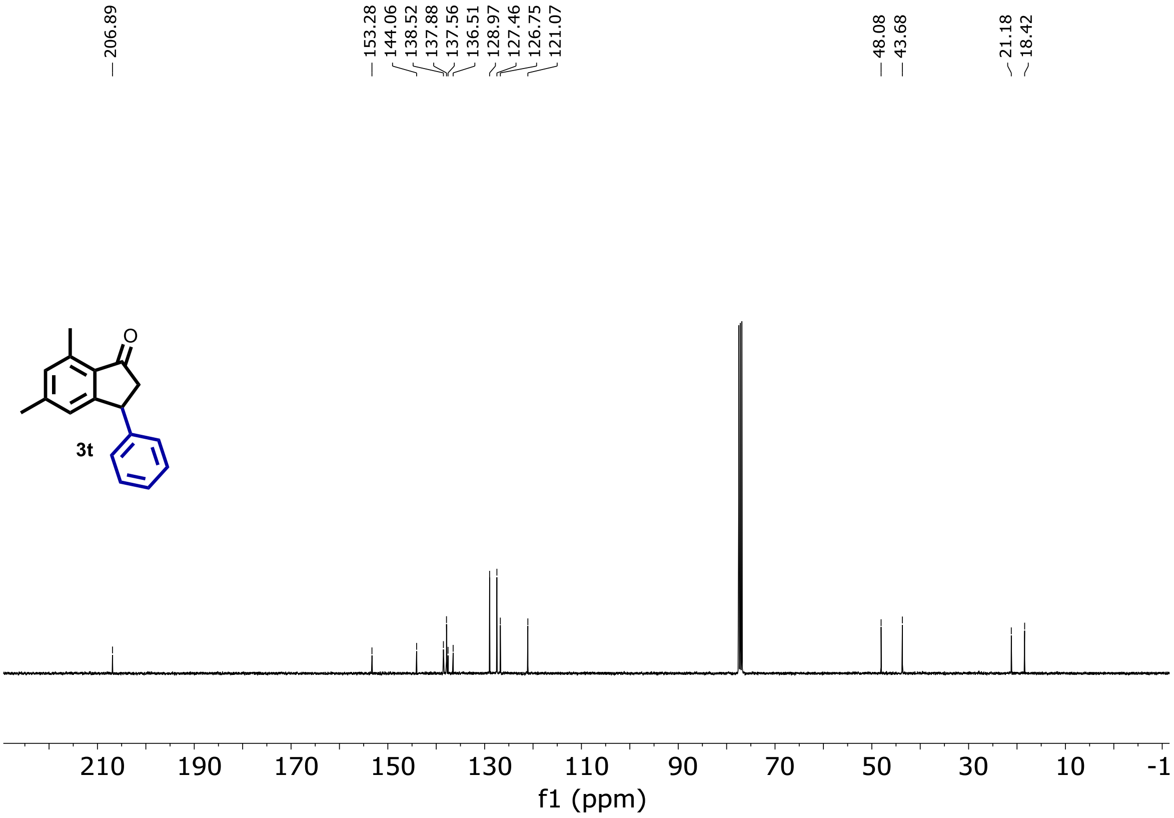

5-methoxy-7-methyl-3-phenyl-2,3-dihydro-1H-inden-1-one (3u): ^{1}H NMR (400 MHz), $CDCl_3$.

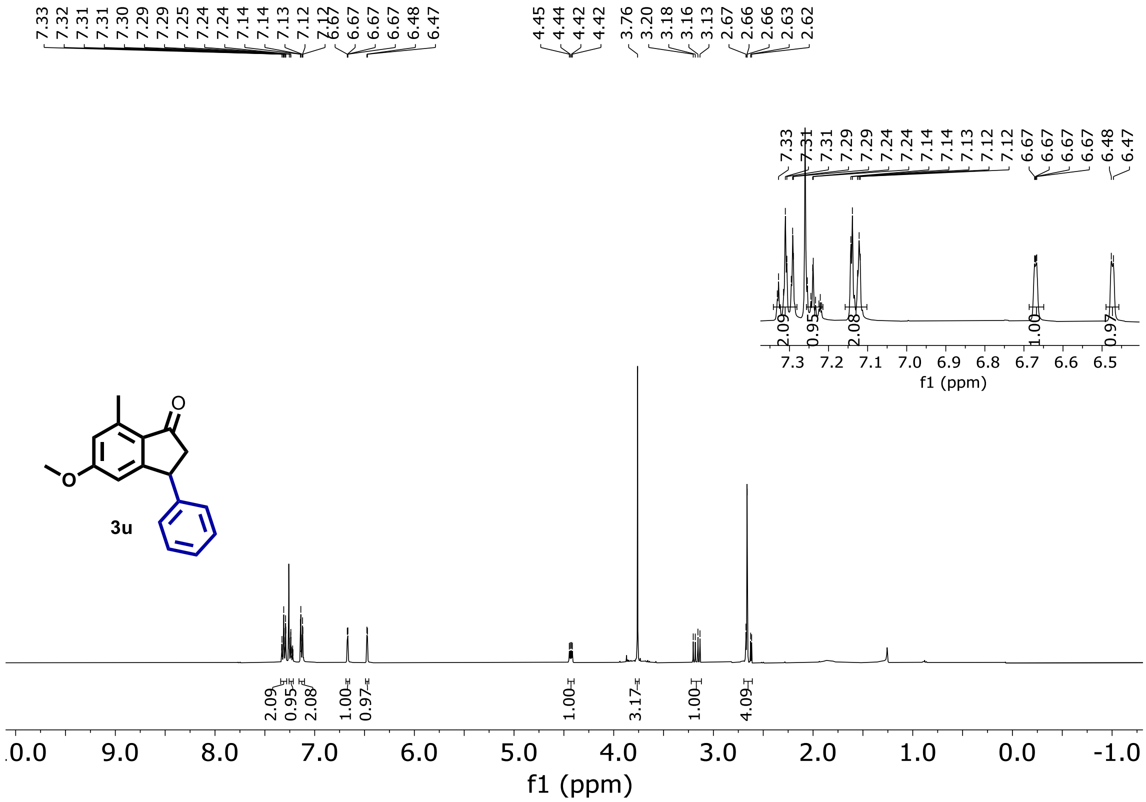

5-methoxy-7-methyl-3-phenyl-2,3-dihydro-1H-inden-1-one (3u): $^{13}C\{^{1}H\}$ NMR (101 MHz), $CDCl_3$.

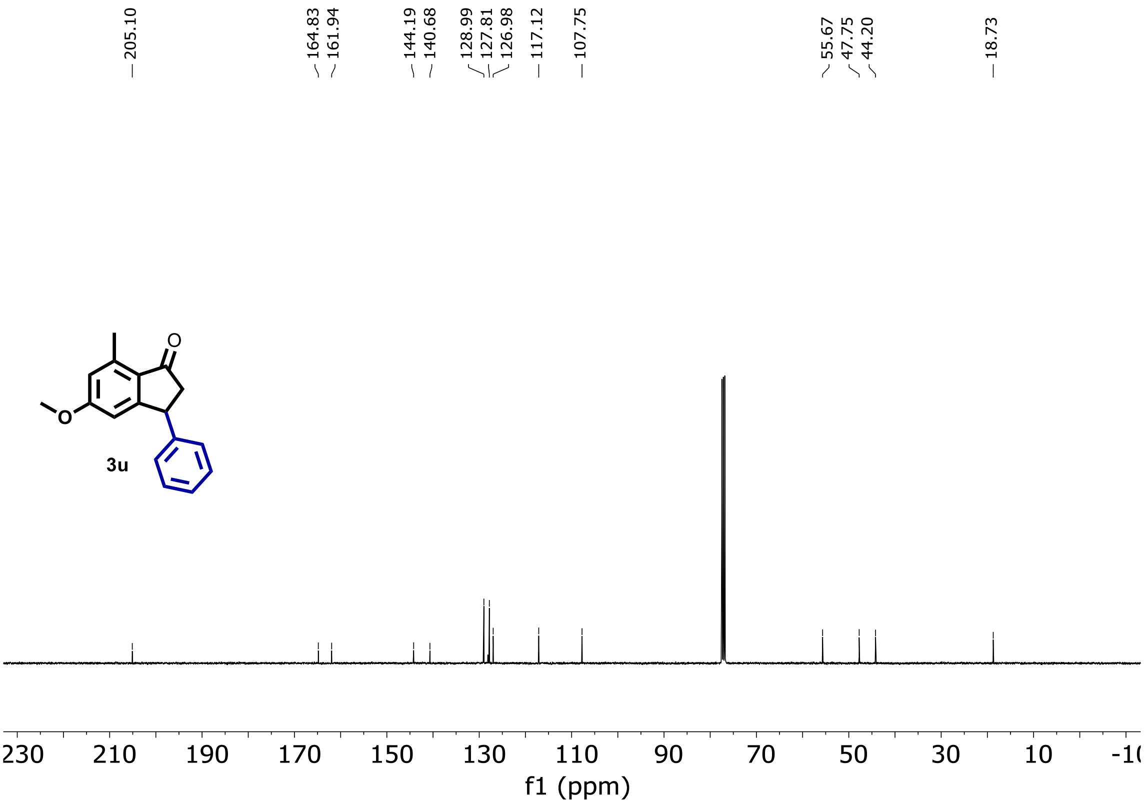

7-chloro-3-phenyl-2,3-dihydro-1H-inden-1-one (3v): ^{1}H NMR (400 MHz), $CDCl_3$.

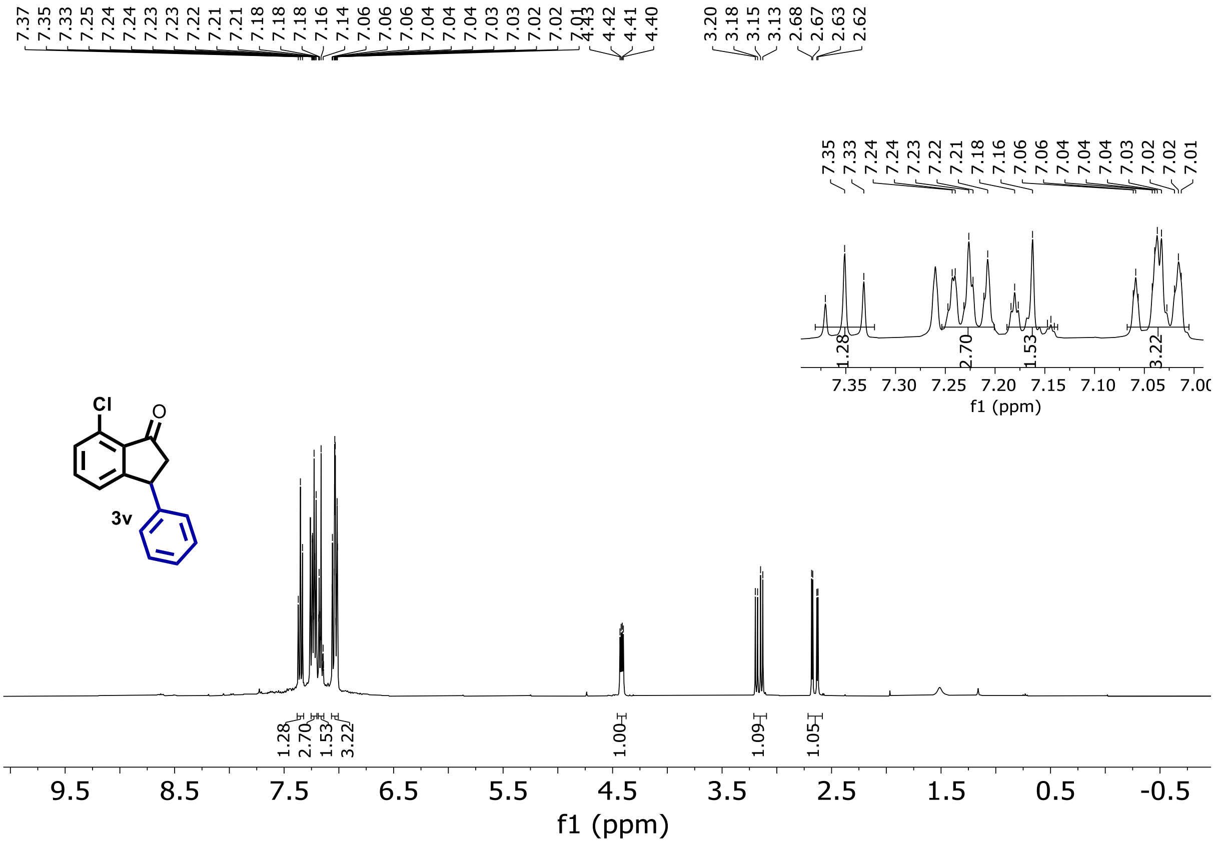

7-chloro-3-phenyl-2,3-dihydro-1H-inden-1-one (3v): ^{13}C{^{1}H} NMR (101 MHz), $CDCl_3$.

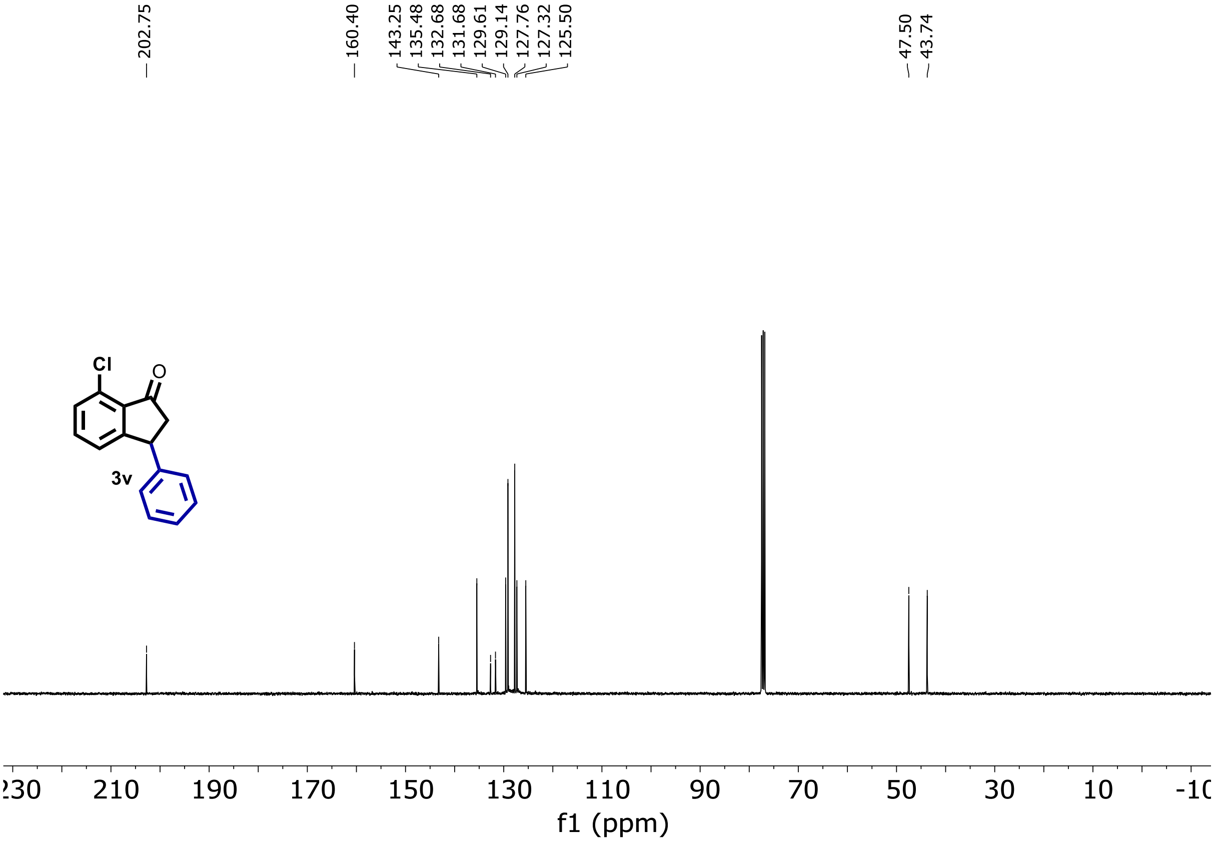

5-chloro-3-phenyl-2,3-dihydro-1H-inden-1-one (3w): ^{1}H NMR (400 MHz), $CDCl_3$.

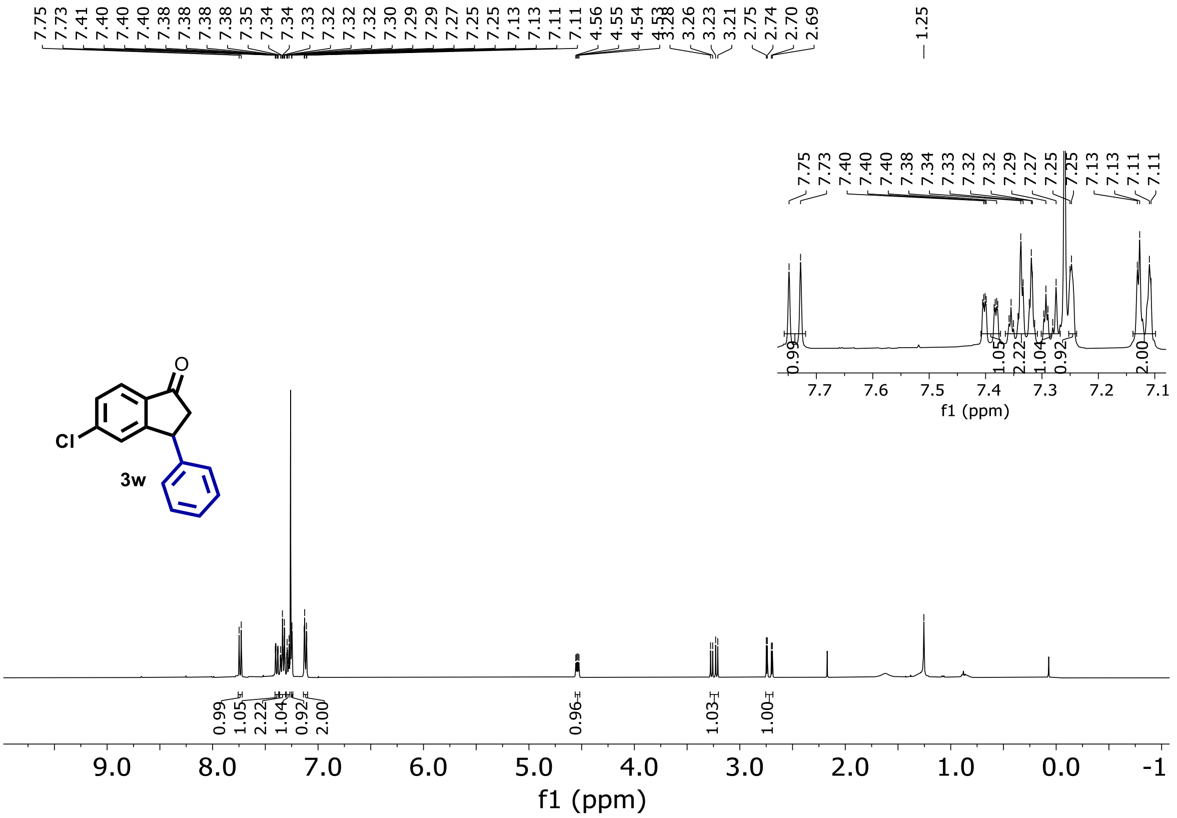

5-chloro-3-phenyl-2,3-dihydro-1H-inden-1-one (3w): ^{13}C{^{1}H} NMR (101 MHz), $CDCl_3$.

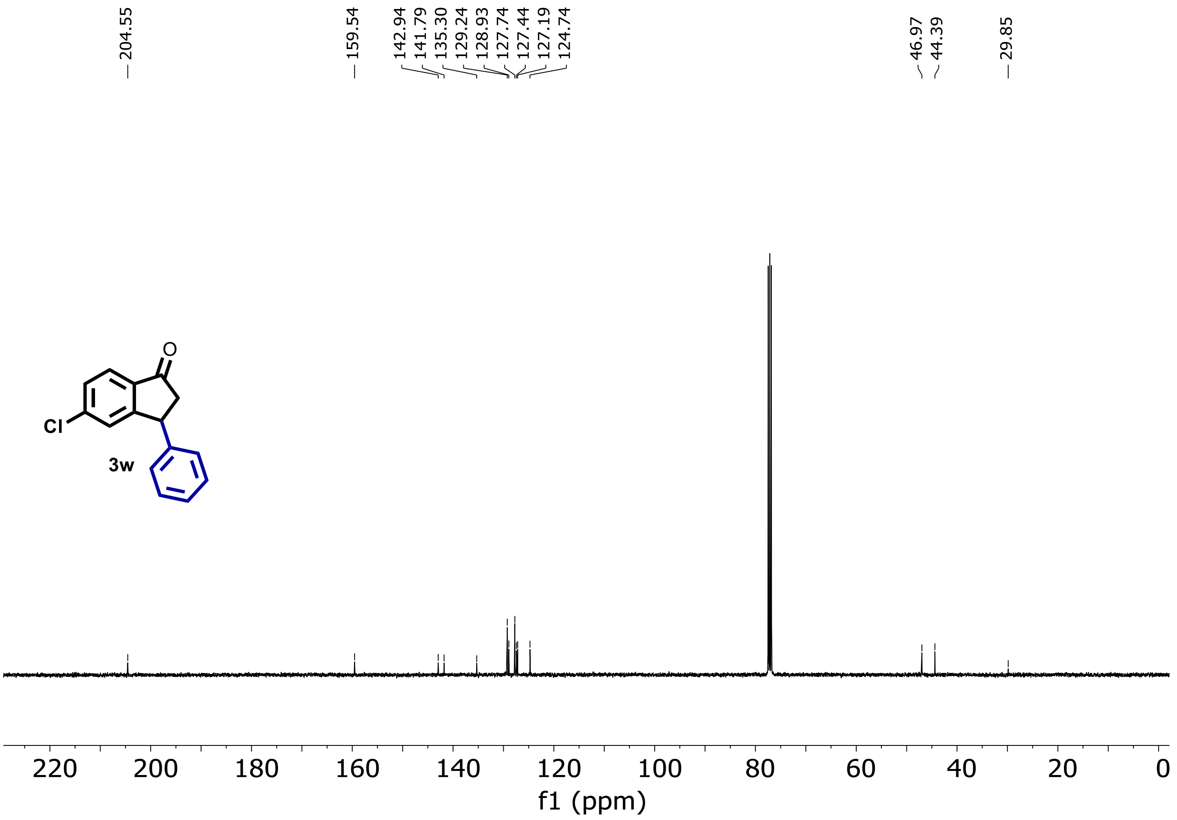

5-fluoro-3-phenyl-2,3-dihydro-1H-inden-1-one (3x): ^{1}H NMR (400 MHz), CDCl$_3$.

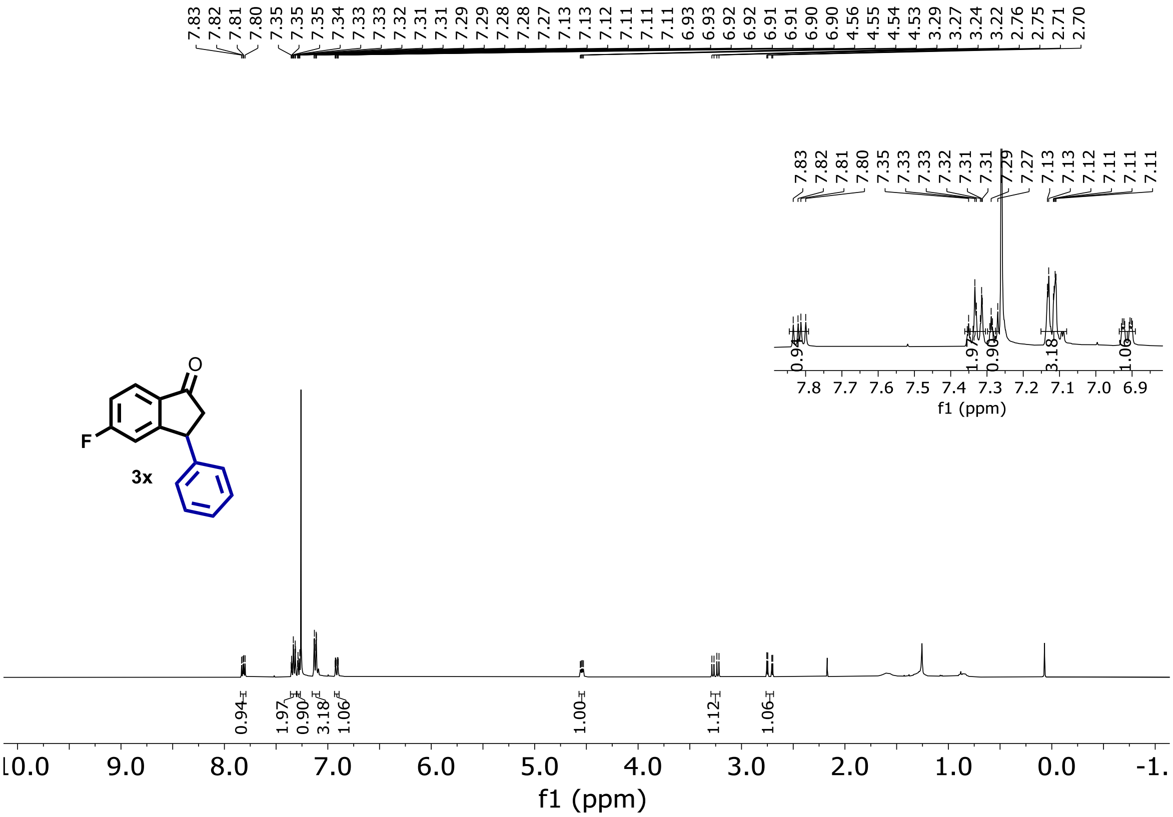

5-fluoro-3-phenyl-2,3-dihydro-1H-inden-1-one (3x): ^{19}F NMR (377 MHz), CDCl$_3$.

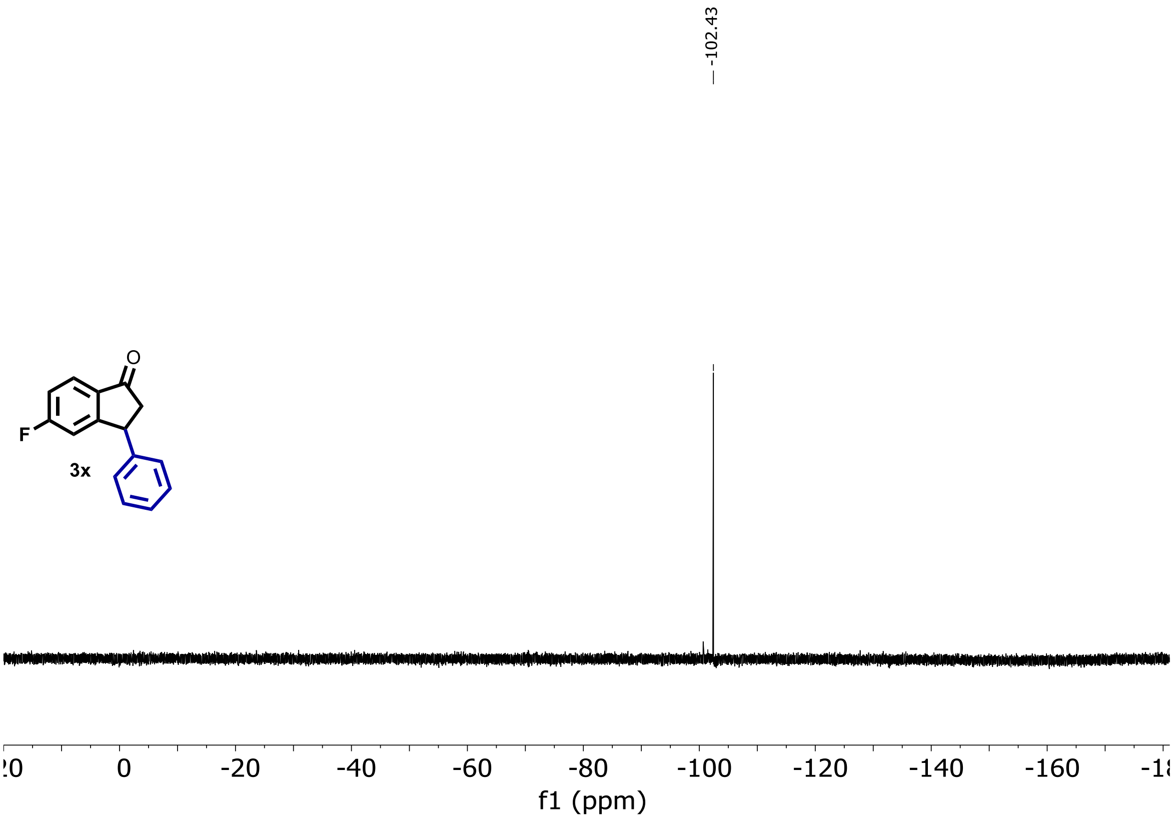

5-fluoro-3-phenyl-2,3-dihydro-1H-inden-1-one (3x): $^{13}C\{^1H\}$ NMR (101 MHz), $CDCl_3$.

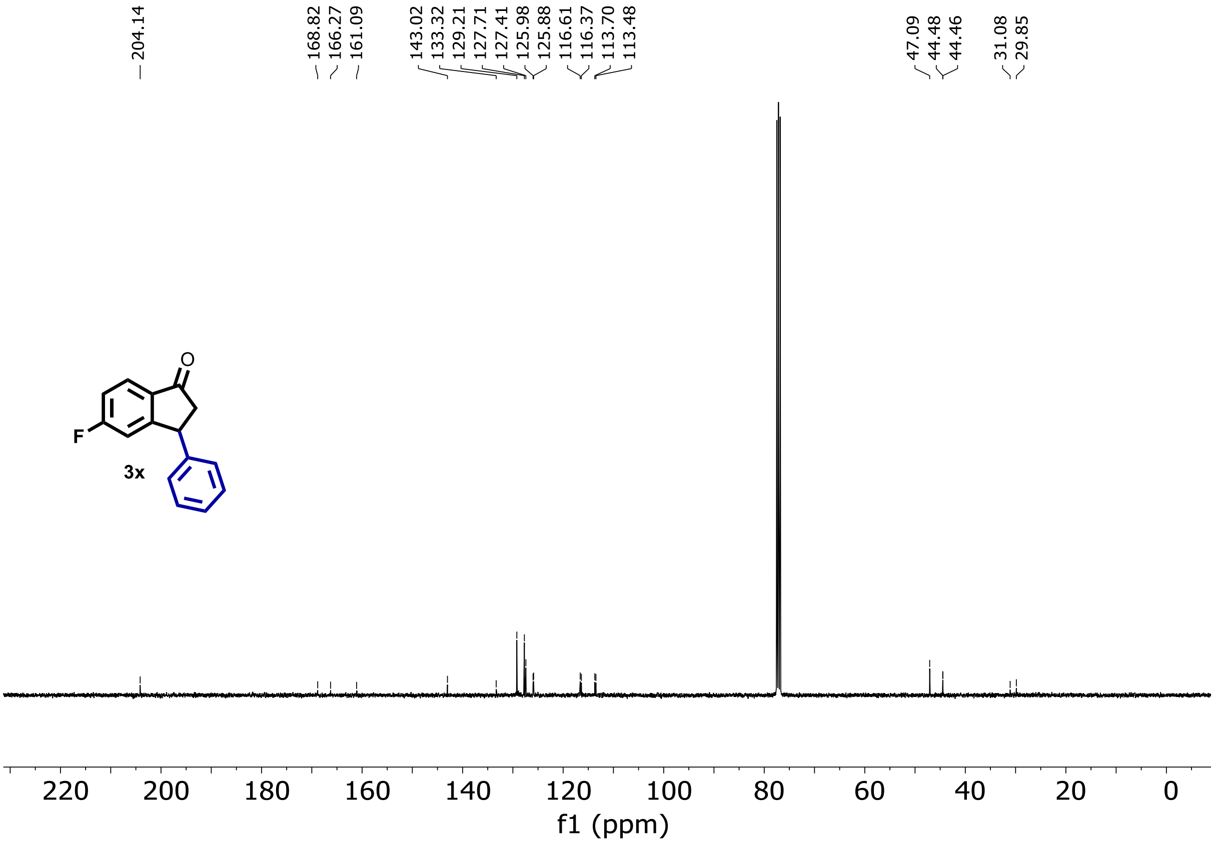

5-(tert-butyl)-3-(4-(tert-butyl)phenyl)-2,3-dihydro-1H-inden-1-one (3y): ^{1}H NMR (400 MHz), $CDCl_3$.

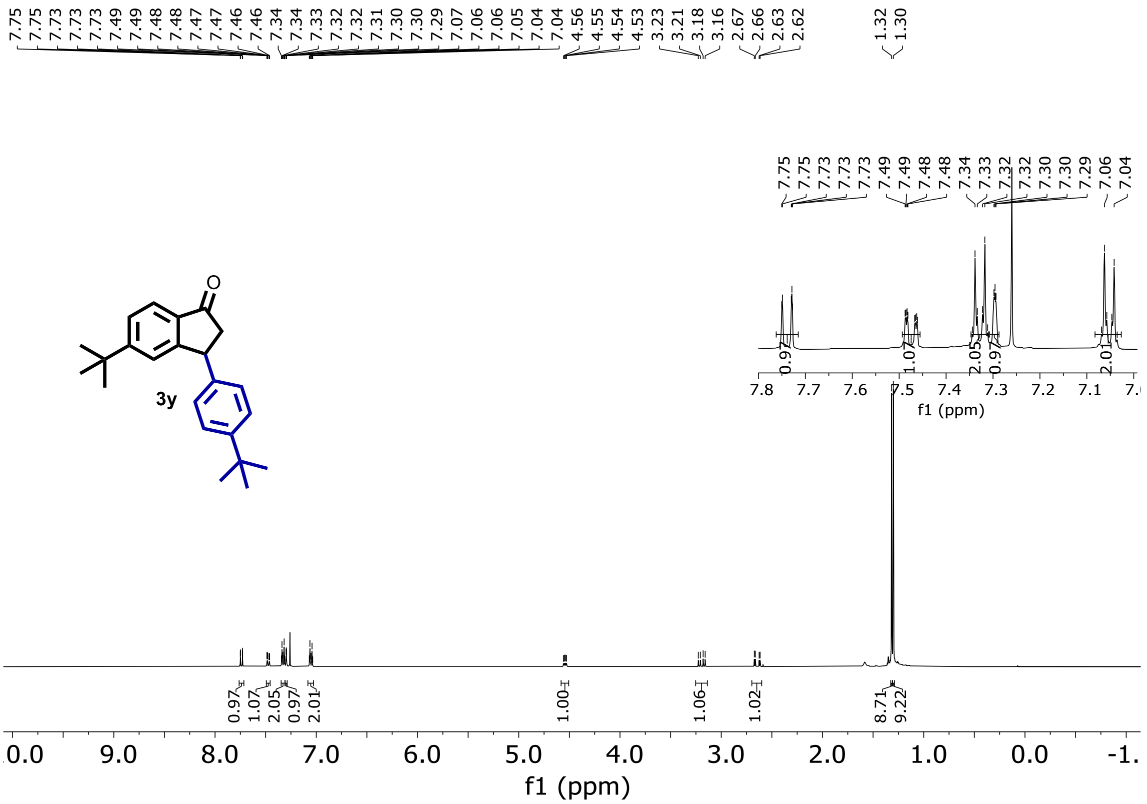

5-(tert-butyl)-3-(4-(tert-butyl)phenyl)-2,3-dihydro-1H-inden-1-one (3y): $^{13}C\{^{1}H\}$ NMR (101 MHz), $CDCl_3$.

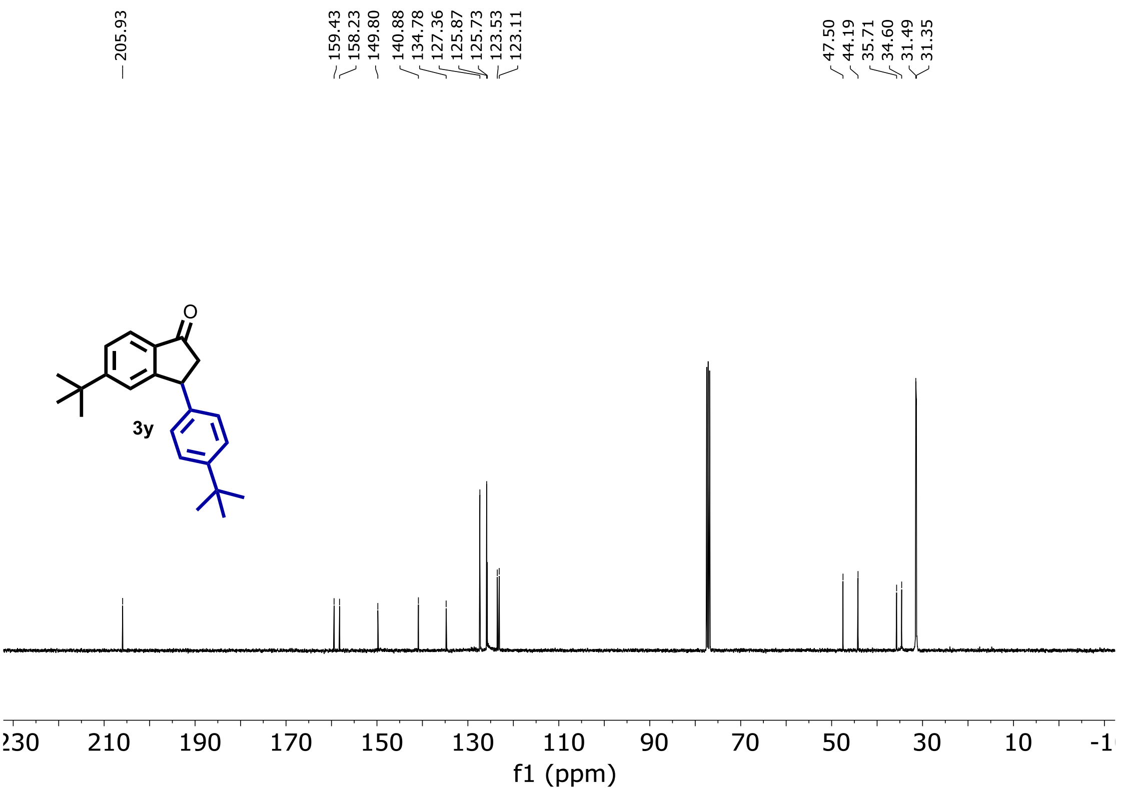

3-(4-(tert-butyl)phenyl)-4,6-dimethyl-2,3-dihydro-1H-inden-1-one (3z): ^{1}H NMR (400 MHz), $CDCl_3$.

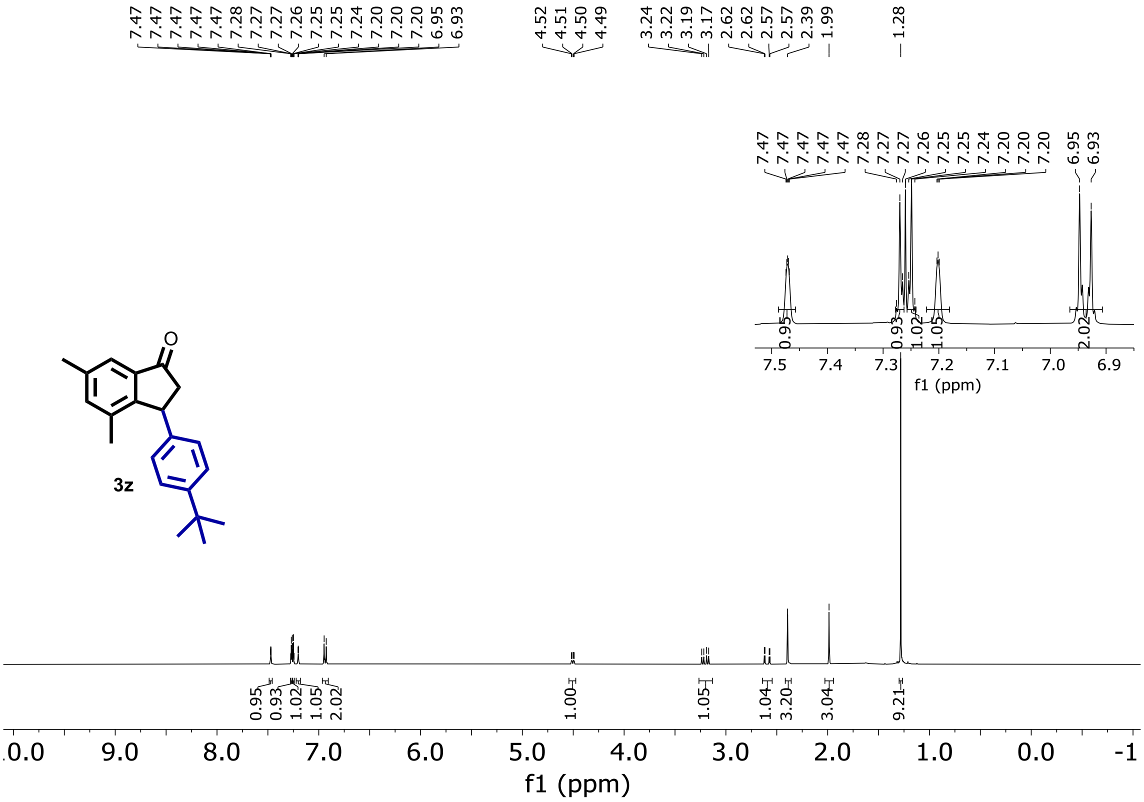

3-(4-(tert-butyl)phenyl)-4,6-dimethyl-2,3-dihydro-1H-inden-1-one (3z): ^{13}C{^{1}H} NMR (101 MHz), $CDCl_3$.

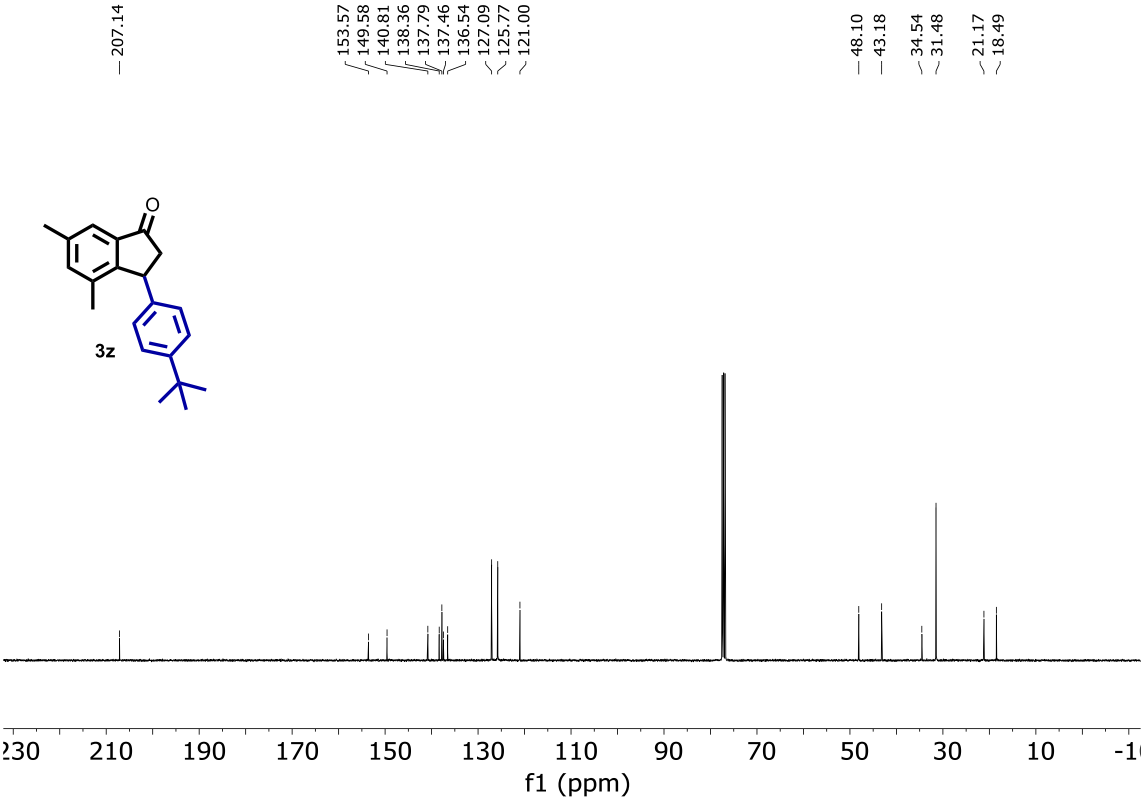

5-isopropoxy-3-(3-methoxyphenyl)-2,3-dihydro-1H-inden-1-one (3aa): ^{1}H NMR (400 MHz), CDCl$_3$.

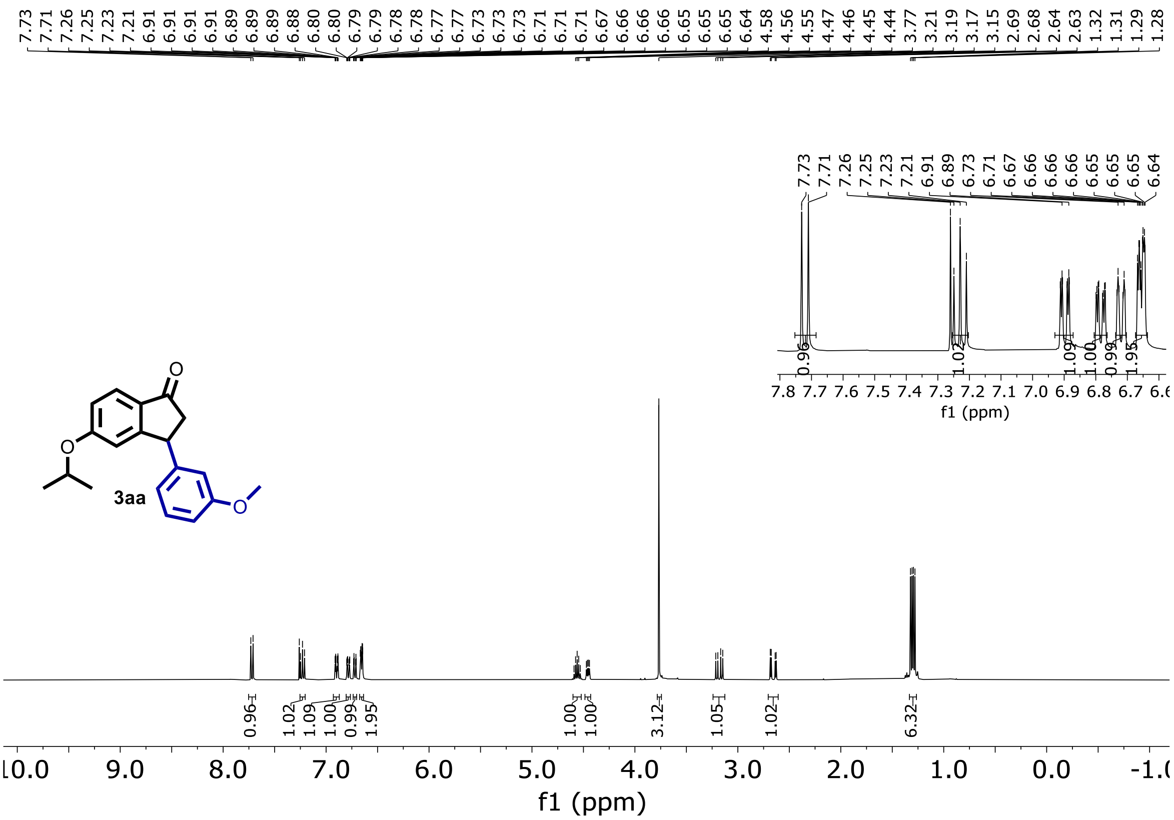

5-isopropoxy-3-(3-methoxyphenyl)-2,3-dihydro-1H-inden-1-one (3aa): ^{13}C{^{1}H} NMR (101 MHz), CDCl$_3$.

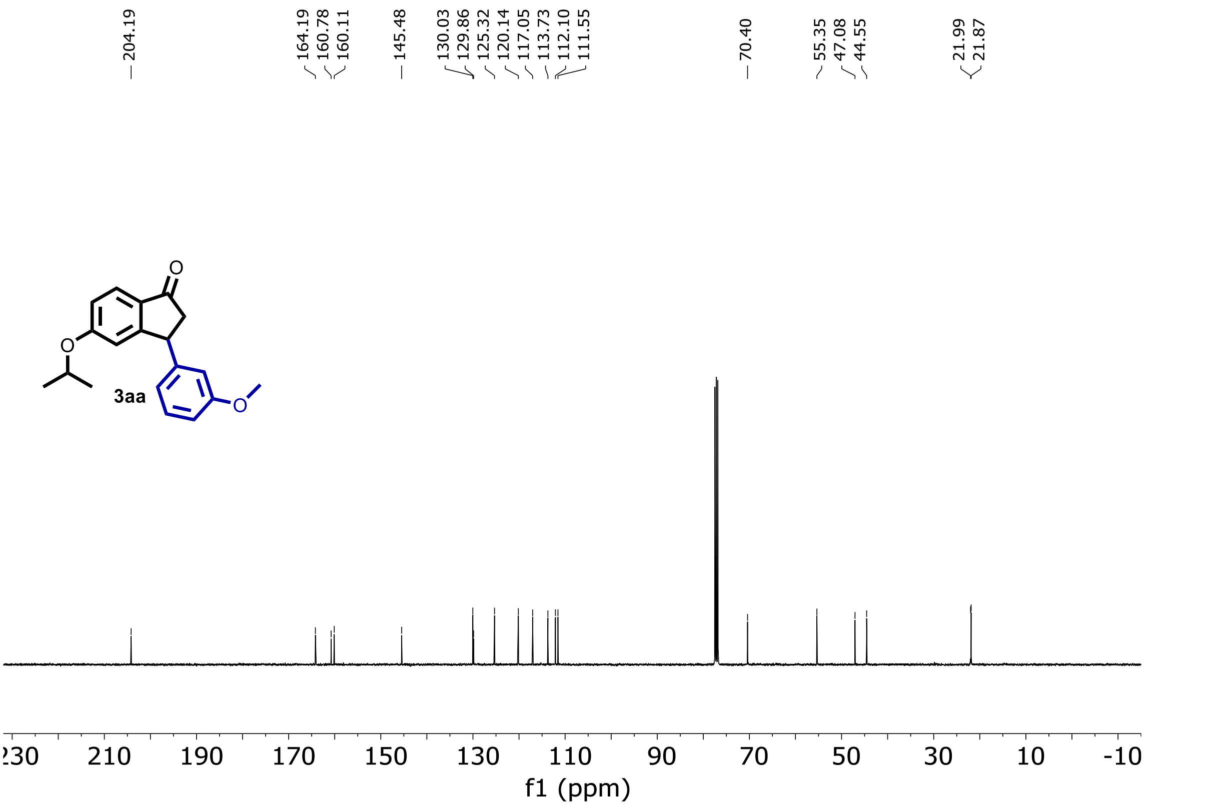

3-(4-(tert-butyl)phenyl)-5-fluoro-2,3-dihydro-1H-inden-1-one (3ab): ^{1}H NMR (400 MHz), $CDCl_3$.

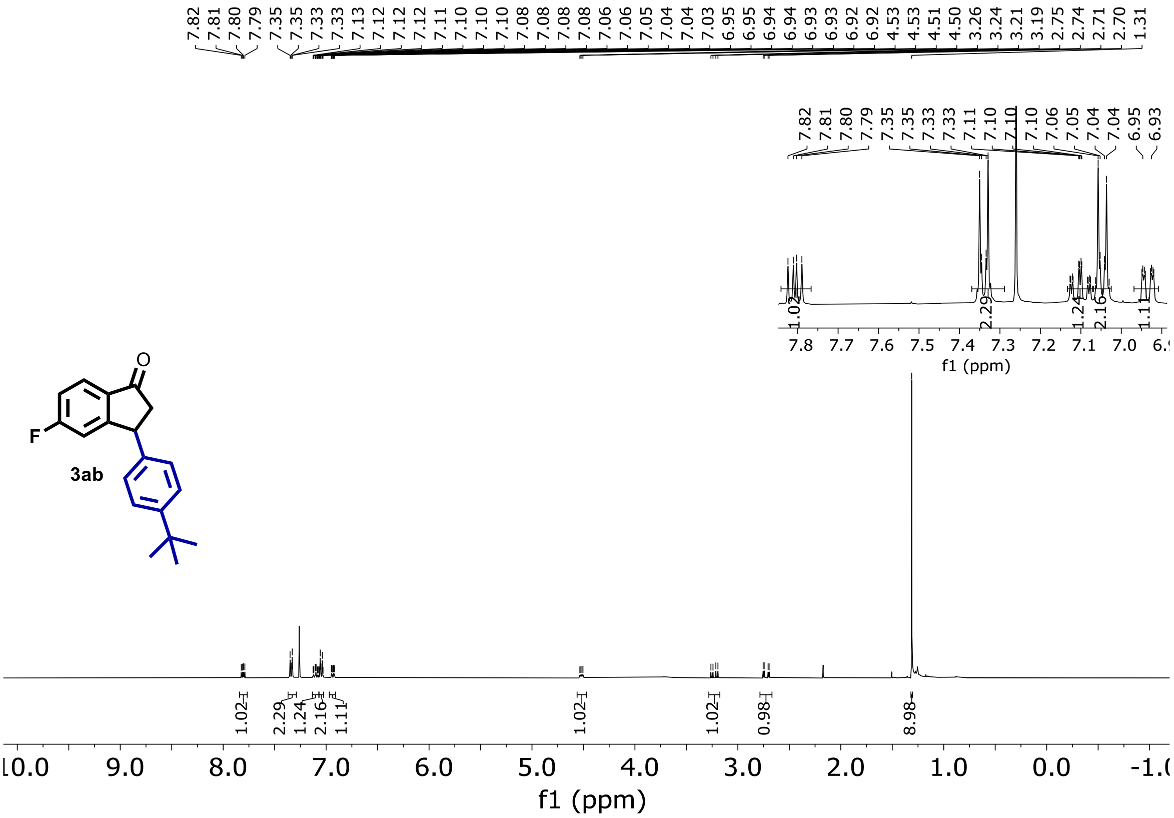

3-(4-(tert-butyl)phenyl)-5-fluoro-2,3-dihydro-1H-inden-1-one (3ab): ^{19}F NMR (377 MHz), $CDCl_3$.

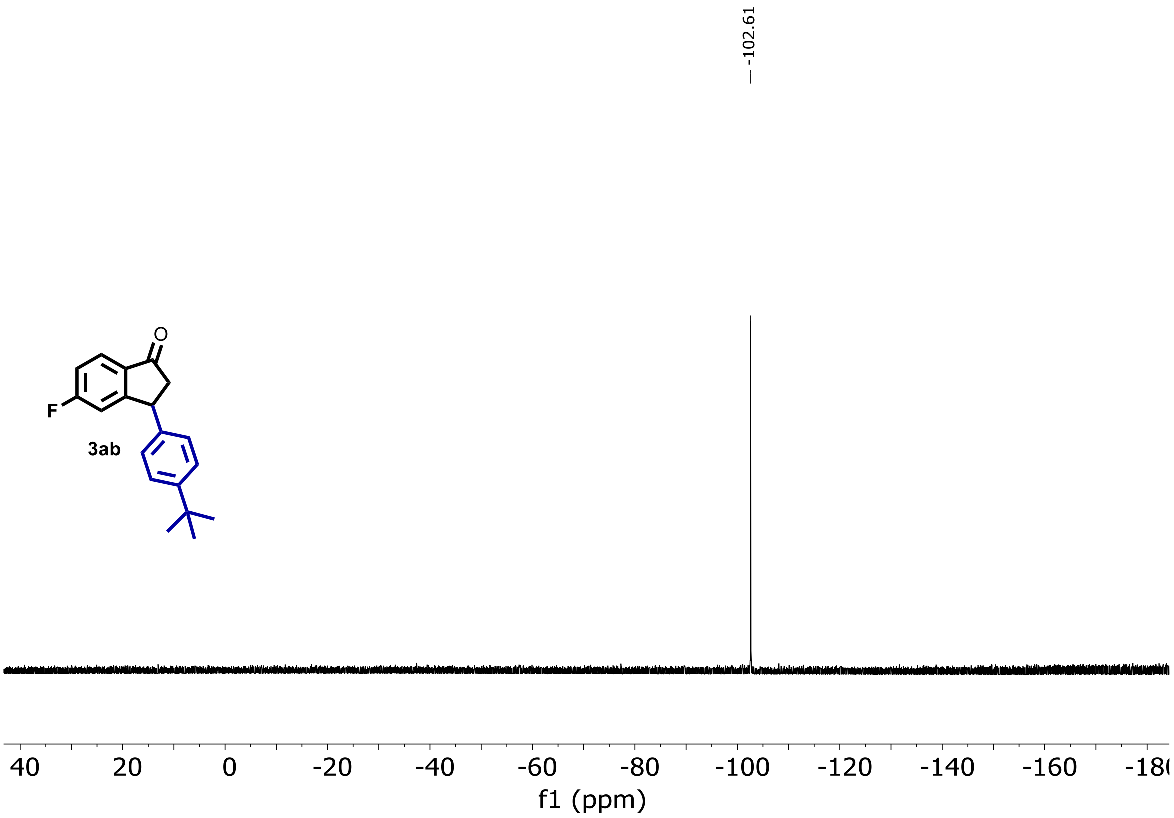

3-(4-(tert-butyl)phenyl)-5-fluoro-2,3-dihydro-1H-inden-1-one (3ab): $^{13}C\{^{1}H\}$ NMR (101 MHz), $CDCl_3$.

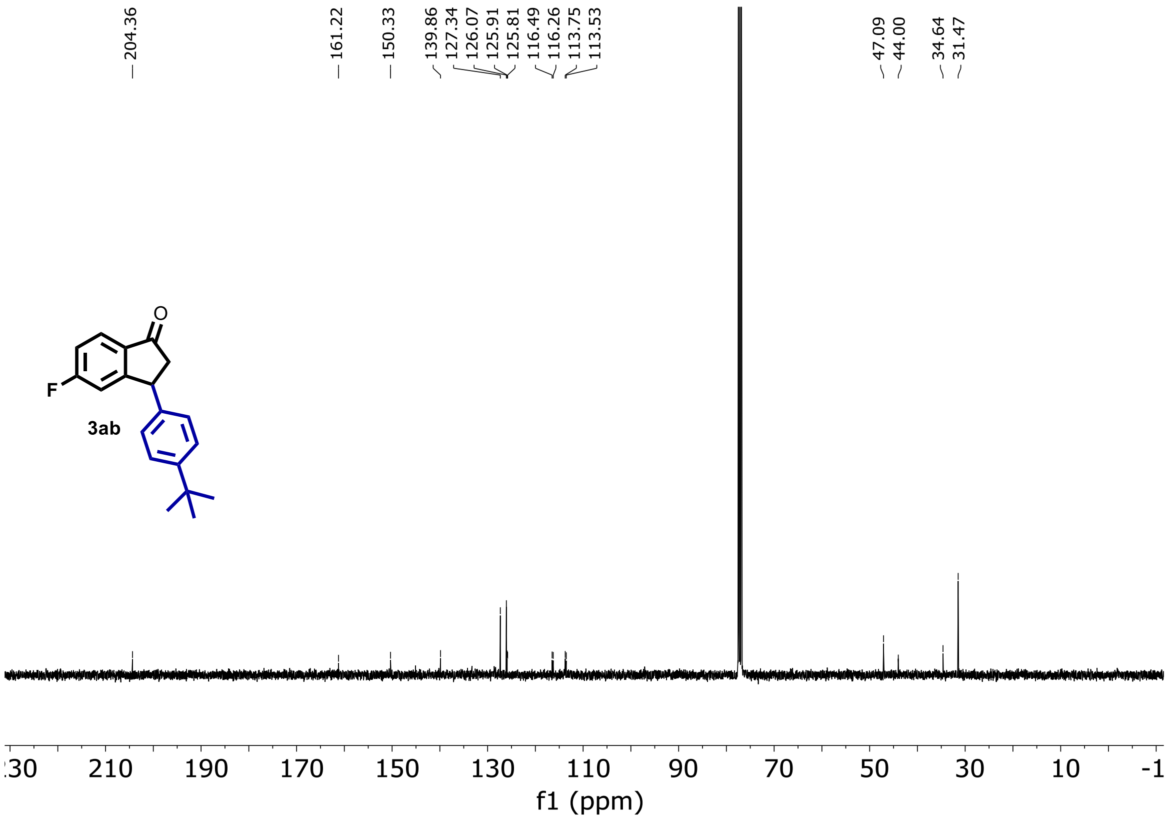

3-(3,5-difluorophenyl)-4,6-dimethyl-2,3-dihydro-1H-inden-1-one (3ac): ^{1}H NMR (400 MHz), $CDCl_3$.

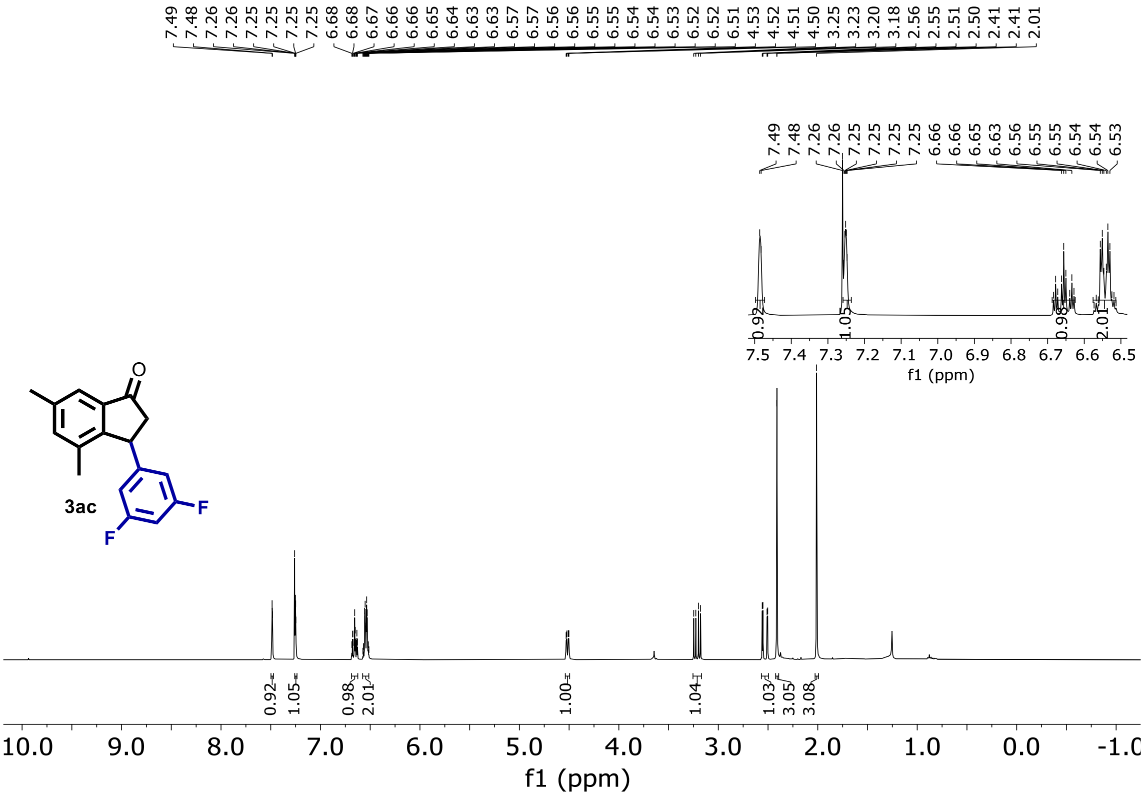

3-(3,5-difluorophenyl)-4,6-dimethyl-2,3-dihydro-1H-inden-1-one (3ac): ^{19}F NMR (377 MHz), $CDCl_3$.

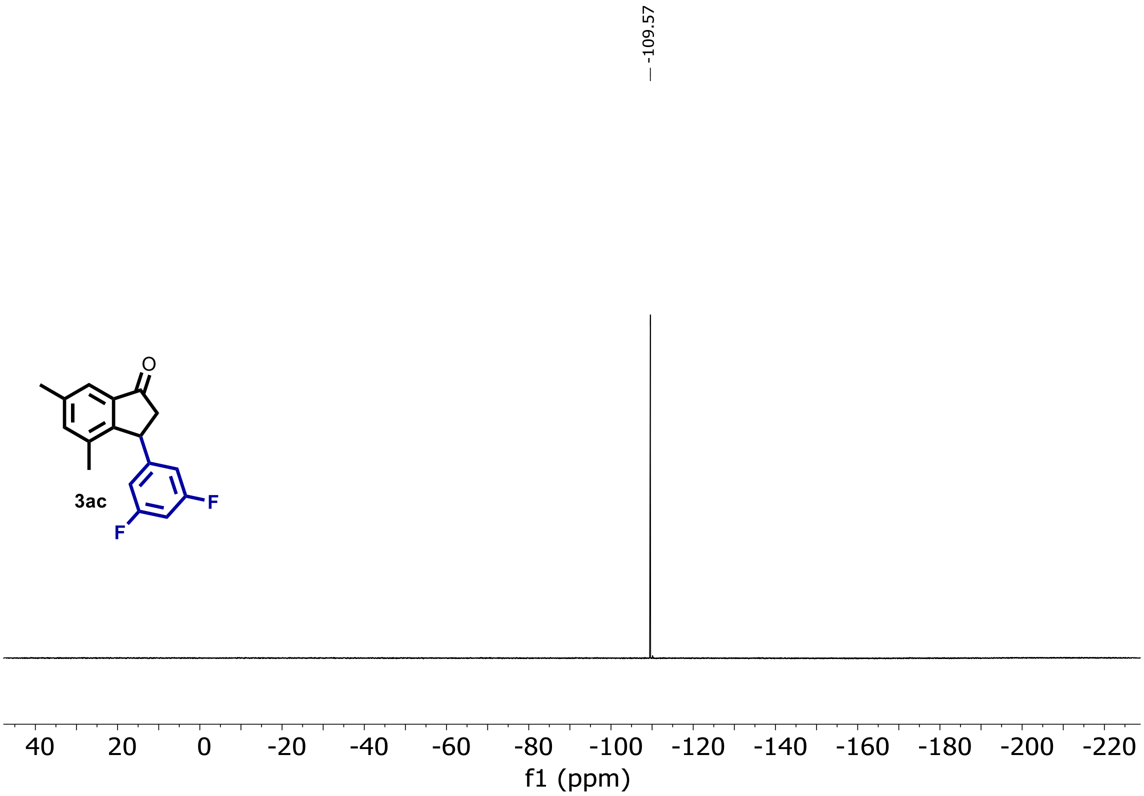

3-(3,5-difluorophenyl)-4,6-dimethyl-2,3-dihydro-1H-inden-1-one (3ac): $^{13}C\{^{1}H\}$ NMR (101 MHz), $CDCl_3$.

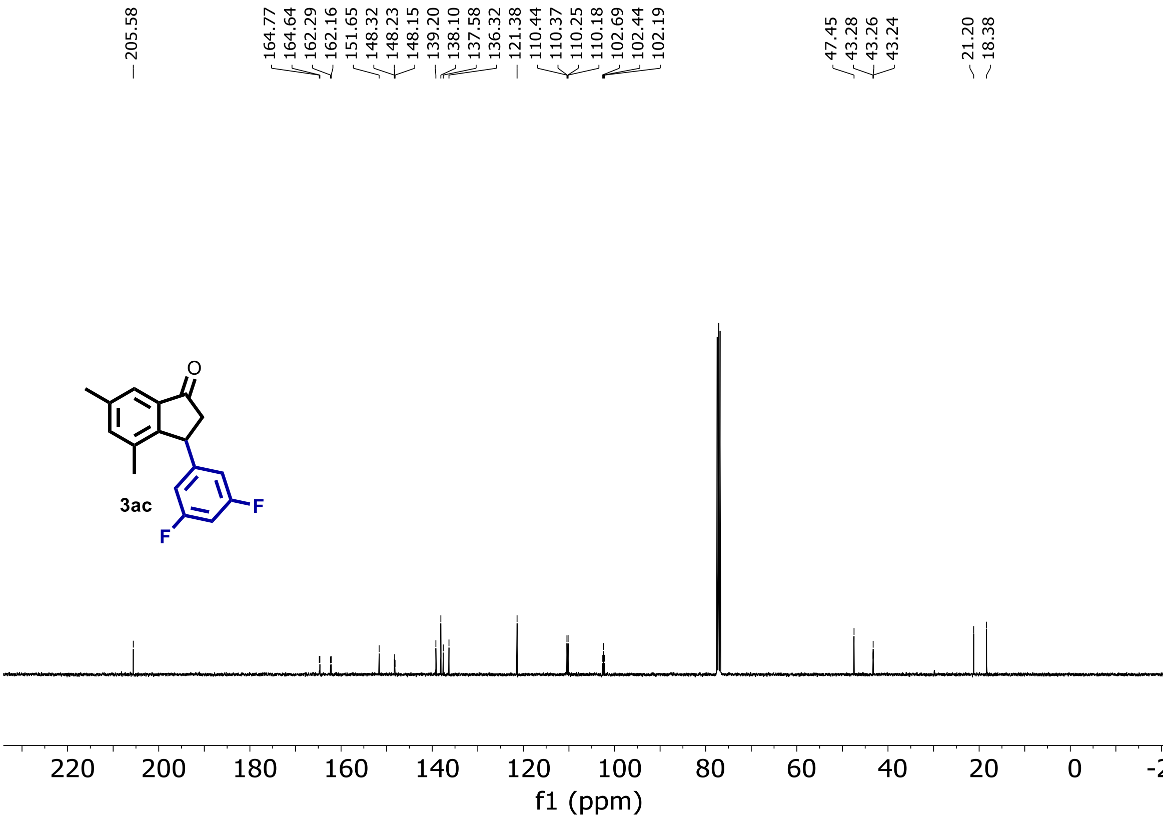

5-(tert-butyl)-3-(4-fluorophenyl)-2,3-dihydro-1H-inden-1-one (3ad): ^{1}H NMR (400 MHz), $CDCl_3$.

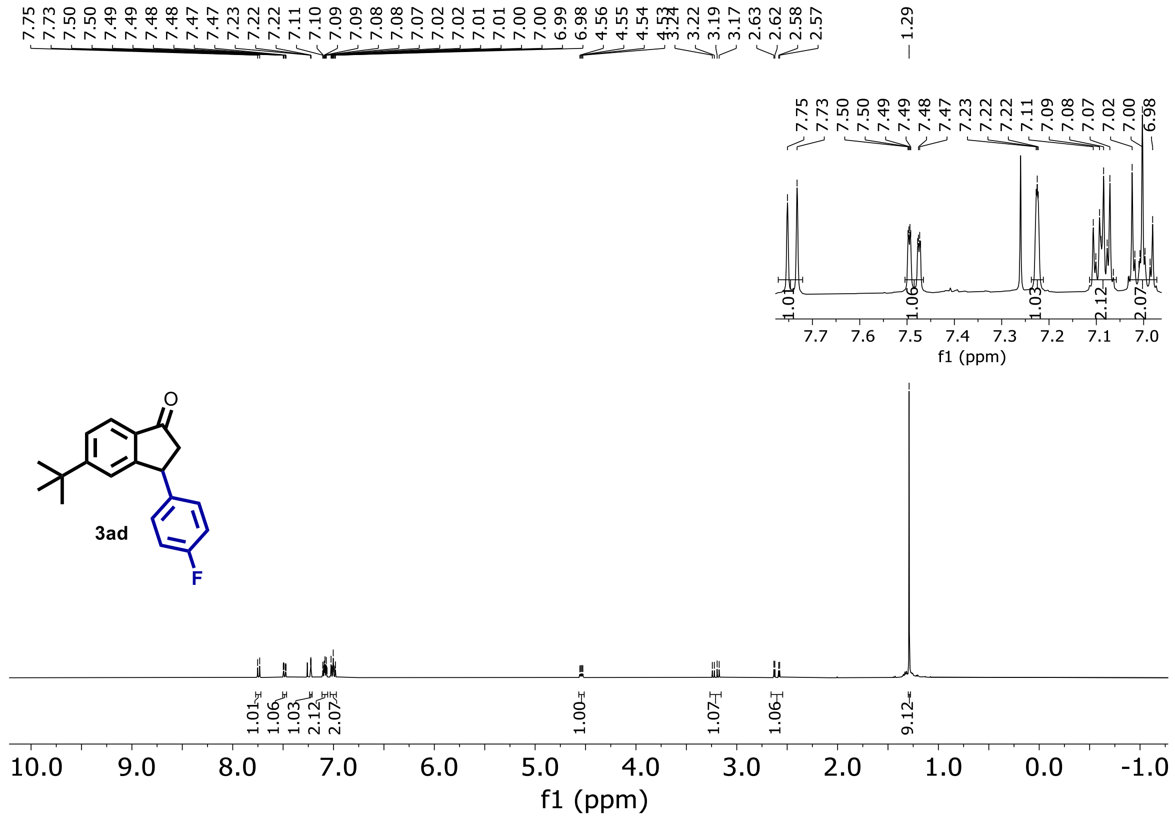

5-(tert-butyl)-3-(4-fluorophenyl)-2,3-dihydro-1H-inden-1-one (3ad): ^{19}F NMR (377 MHz), $CDCl_3$

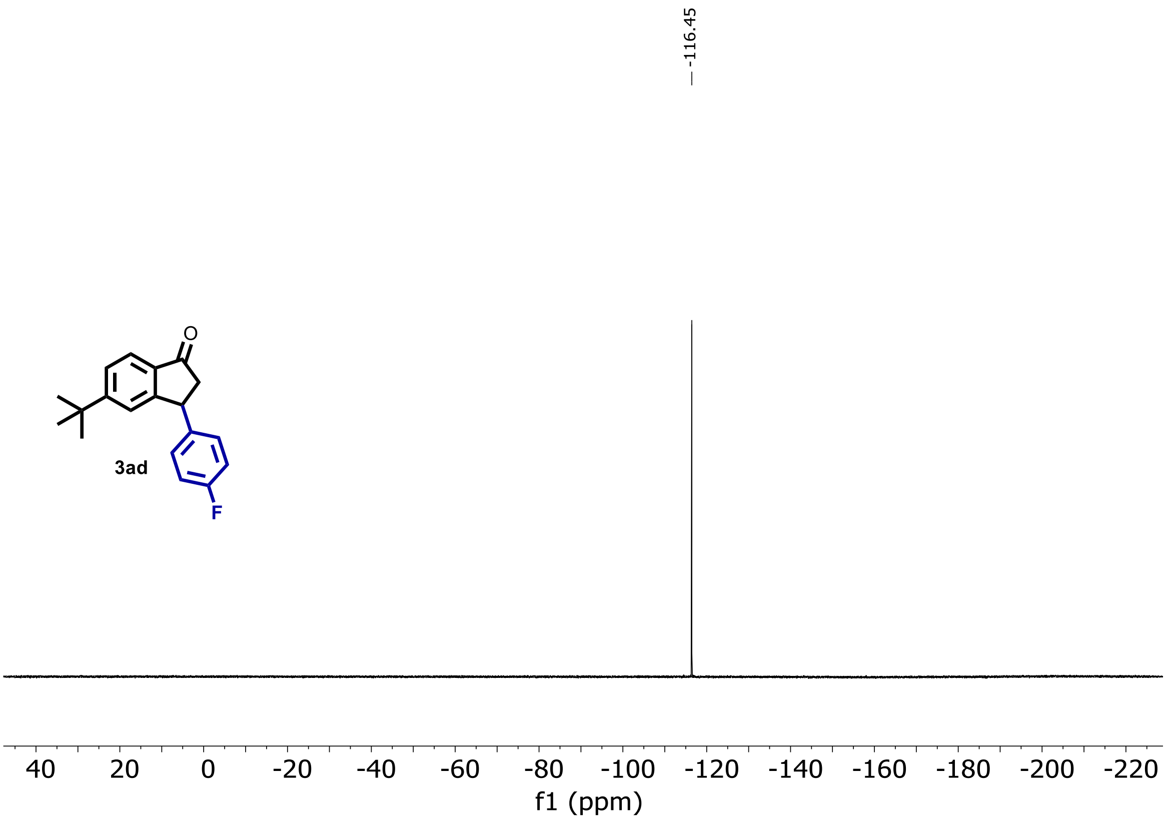

5-(tert-butyl)-3-(4-fluorophenyl)-2,3-dihydro-1H-inden-1-one (3ad): $^{13}C\{^{1}H\}$ NMR (101 MHz), $CDCl_3$.

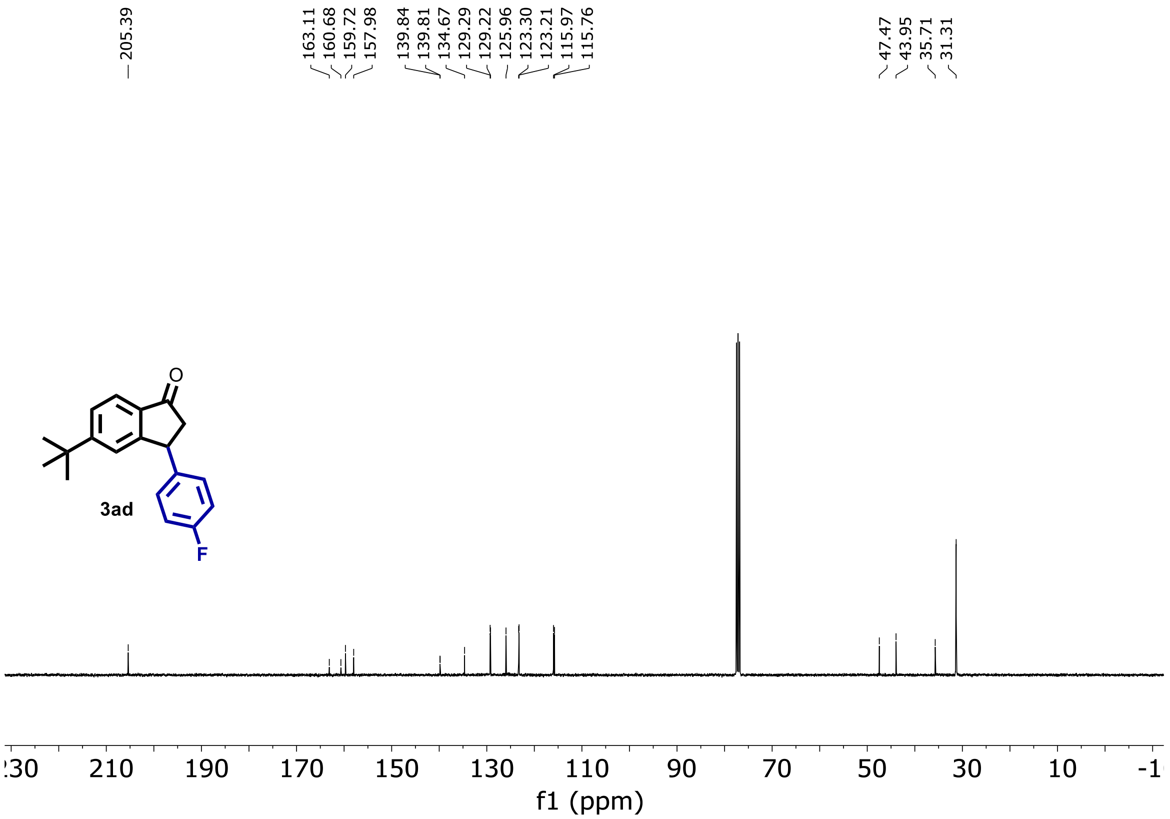

7-methoxy-3-(4-(4,4,5,5-tetramethyl-1,3,2-dioxaborolan-2-yl)phenyl)-2,3-dihydro-1H-inden-1-one (3ae): ^{1}H NMR (400 MHz), CDCl$_3$.

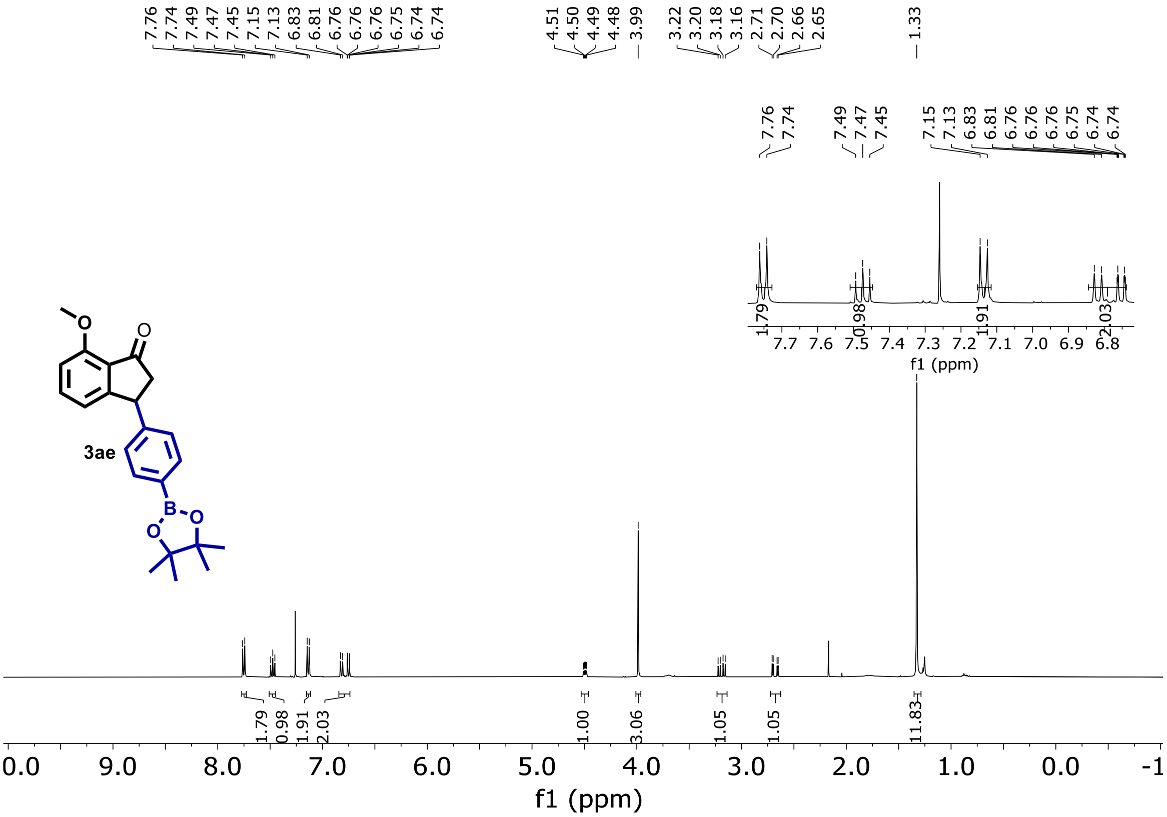

7-methoxy-3-(4-(4,4,5,5-tetramethyl-1,3,2-dioxaborolan-2-yl)phenyl)-2,3-dihydro-1H-inden-1-one (3ae): ^{11}B NMR (128 MHz), CDCl$_3$.

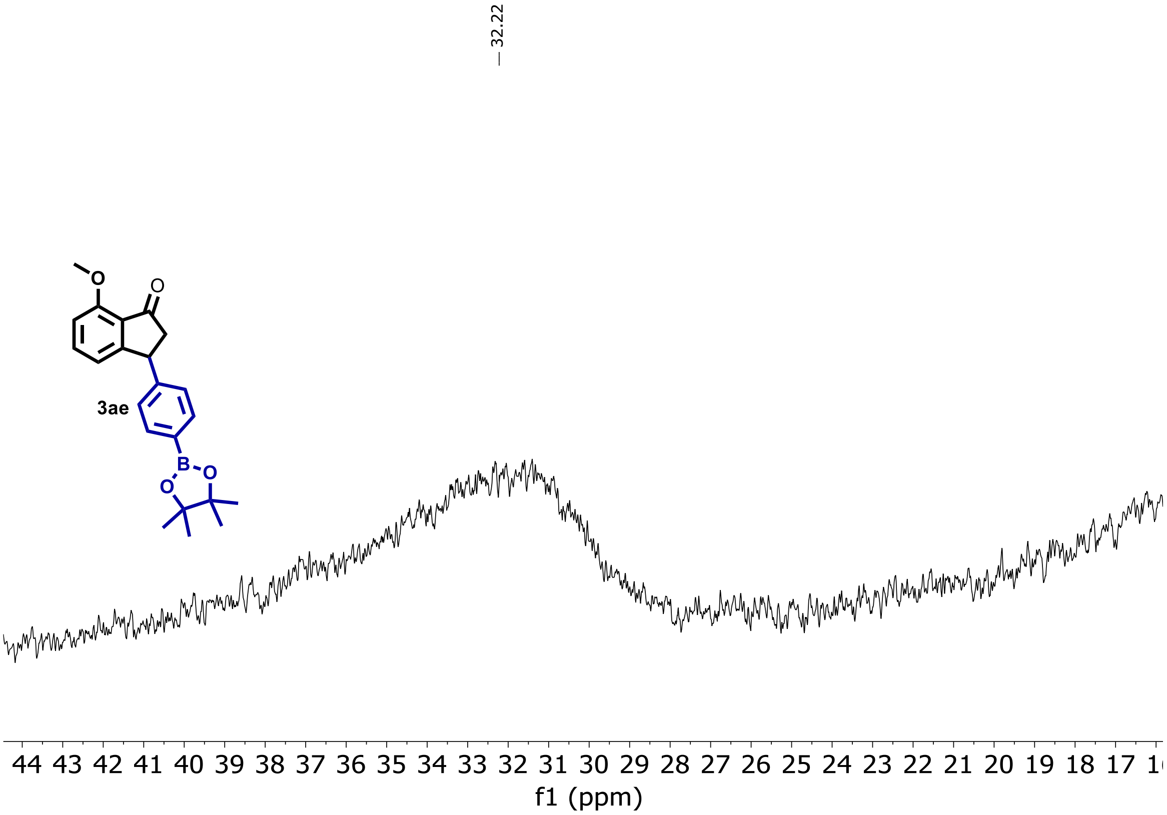

7-methoxy-3-(4-(4,4,5,5-tetramethyl-1,3,2-dioxaborolan-2-yl)phenyl)-2,3-dihydro-1H-inden-1-one (3ae): $^{13}C\{^{1}H\}$ NMR (101 MHz), $CDCl_3$.

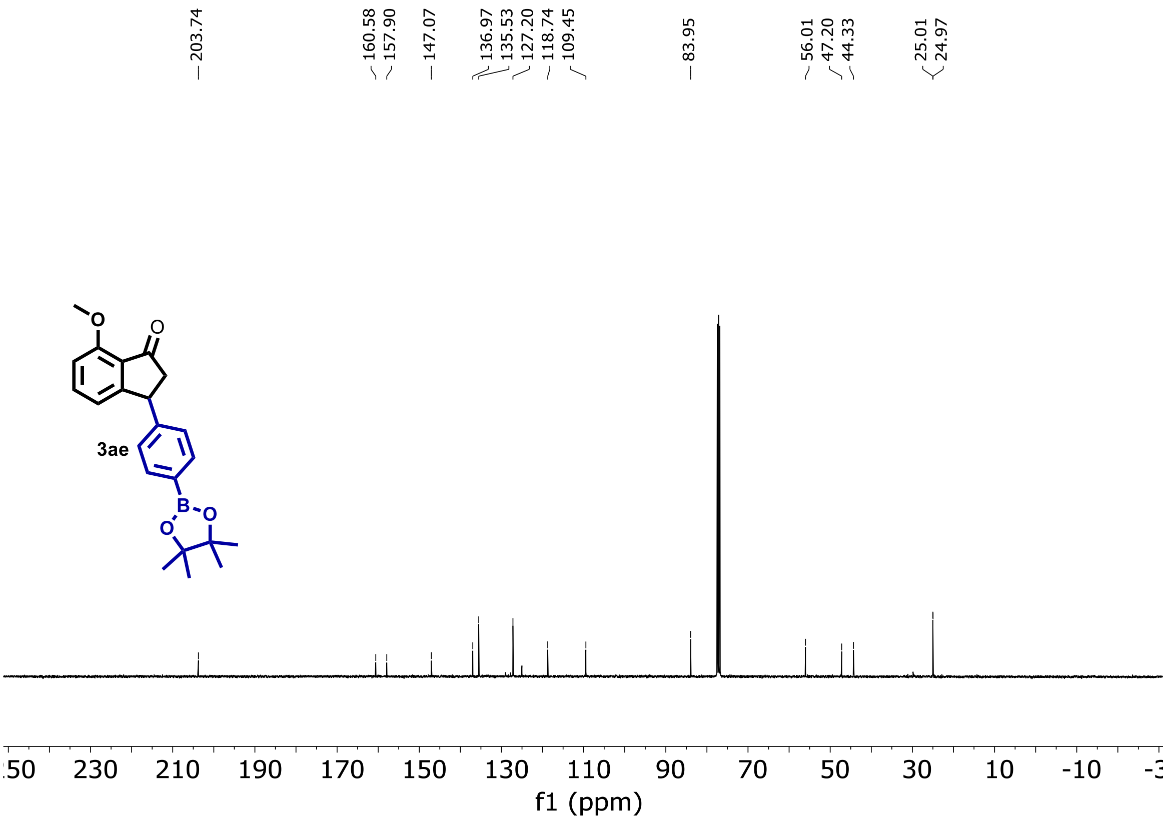

Supplement: Supplementary file 1 [file jo5c01749_si_001.pdf]
